# Supplementary material for: Risks of specific congenital anomalies in offspring of women with diabetes: A systematic review and meta-analysis of population-based studies including over 80 million births
Source: PLoS Med. 2022 Feb 1;19(2):e1003900. doi: 10.1371/journal.pmed.1003900 (PMC8806075; doi:10.1371/journal.pmed.1003900)
Supplement: S1 Fig — Fig A: Risk of bias summary: Effect on congenital anomalies in offspring of women with pre-gestational diabetes. Fig B: Risk of bias summary: Effect on congenital heart defects in offspring of women with pre-gestational diabetes. Fig C: Risk of bias summary: The effect on congenital anomalies in offspring of women with gestational diabetes. Fig D: Risk of bias summary: The effect on congenital heart defects in offspring of women with gestational diabetes. Fig E: Funnel plots of the relative risks in population-based studies for pre-gestational diabetes mellitus and the risk of congenital anomalies. Fig F: Funnel plots of the relative risks in population-based studies for gestational diabetes mellitus and the risk of congenital anomalies. Fig G: Forest plot of the relative risks in population-based studies for maternal pre-gestational diabetes and the risk of any type of congenital heart defect—G1: heterotaxia; G2: conotruncal defects; G3: truncus arteriosus; G4: transposition of great vessels; G5: tetralogy of Fallot; G6: atrioventricular septal defects; G7: anomalous pulmonary venous return; G8: left ventricular outflow tract defect; G9: coarctation of aorta; G10: hypoplastic left heart; G11: right ventricular outflow tract defect; G12: pulmonary artery anomalies; G13: pulmonary valve stenosis; G14: septal defects; G15: ventricular septal defects; G16: atrial septal defects; G17: ventricular septal defect and atrial septal defect; G18: single ventricle. Fig H: Forest plot of the relative risks in population-based studies for maternal pre-gestational diabetes and the risk of other type-specific congenital anomalies—H1: nervous system defects; H2: neural tube defects; H3: anencephaly; H4: encephalocele; H5: spina bifida; H6: hydrocephaly; H7: holoprosencephaly; H8: eye, ear, face, and neck defects; H9: orofacial clefts; H10: cleft palate. H11: cleft lip with or without cleft palate; H12: digestive system defects; H13: diaphragmatic hernia; H14: abdominal wall defects [file pmed.1003900.s001.pdf]

S1 Fig

This supporting information formed part of the original submission and has been peer reviewed.
We post it as supplied by the authors.

Supplement to: Tie-Ning Zhang, Xin-Mei Huang, Xin-Yi Zhao, Wei Wang, Ri Wen, Shan-Yan Gao.
Risks of specific congenital anomalies in offspring of women with diabetes: A systematic review and meta-analysis of population-based studies including over 80 million births

Risks of specific congenital anomalies in offspring of women with diabetes: A systematic review and meta-analysis of population-based studies including over 80 million birth
Supporting information

Contents

Fig A Risk of bias summary: effect on congenital anomalies in offspring with pre-gestational diabetes……………………………………………………………………………...…pg. 1
Fig B Risk of bias summary: effect on congenital heart defects in offspring with pre-gestational diabetes…………………………………………………………………………………pg. 2
Fig C Risk of bias summary: effect on congenital anomalies in offspring with gestational diabetes…………………………………………………………………………………pg. 3
Fig D Risk of bias summary: effect on congenital heart defects in offspring with gestational diabetes…………………………………………………………………………………pg. 4
Fig E Funnel plots of the relative risks of population-based studies on pr-egestational diabetes and the risk of congenital anomalies………………………………………………………………pg. 5
Fig F Funnel plots of the relative risks of population-based studies on gestational diabetes and the risk of congenital anomalies…………………………………………………………………pg. 6
Fig G Forest plot of the relative risks of population-based studies on maternal pre-gestational diabetes and the risk of any type of congenital heart defects..……….……….……………………pg. 7
Fig H Forest plot of the relative risks of population-based studies on maternal pre-gestational diabetes and the risk of other type-specific congenital anomalies..……….……….…………………pg. 25
Fig I Forest plot of the relative risks of population-based studies on maternal gestational diabetes and the risk of any type of congenital heart defects..……………….…….…………………...pg. 50
Fig J Forest plot of the relative risks of population-based studies on maternal gestational diabetes and the risk of other type-specific congenital anomalies..………….…….………………pg. 65
Fig K1 Forest plot of the relative risks of population-based studies on maternal Type 1 diabetes and the risk of overall congenital anomalies..…….……….…….……………………………pg. 87
Fig K2 Forest plot of the relative risks of population-based studies on maternal Type 1 diabetes and the risk of congenital heart defects..……….…………………………….…………………...pg. 88
Fig J1 Forest plot of the relative risks of population-based studies on maternal Type 2 diabetes and the risk of overall congenital anomalies..…….……….…….……………………………pg. 89
Fig J2 Forest plot of the relative risks of population-based studies on maternal Type 2 diabetes and the risk of congenital heart defects..……….…………………………….……………………pg. 90

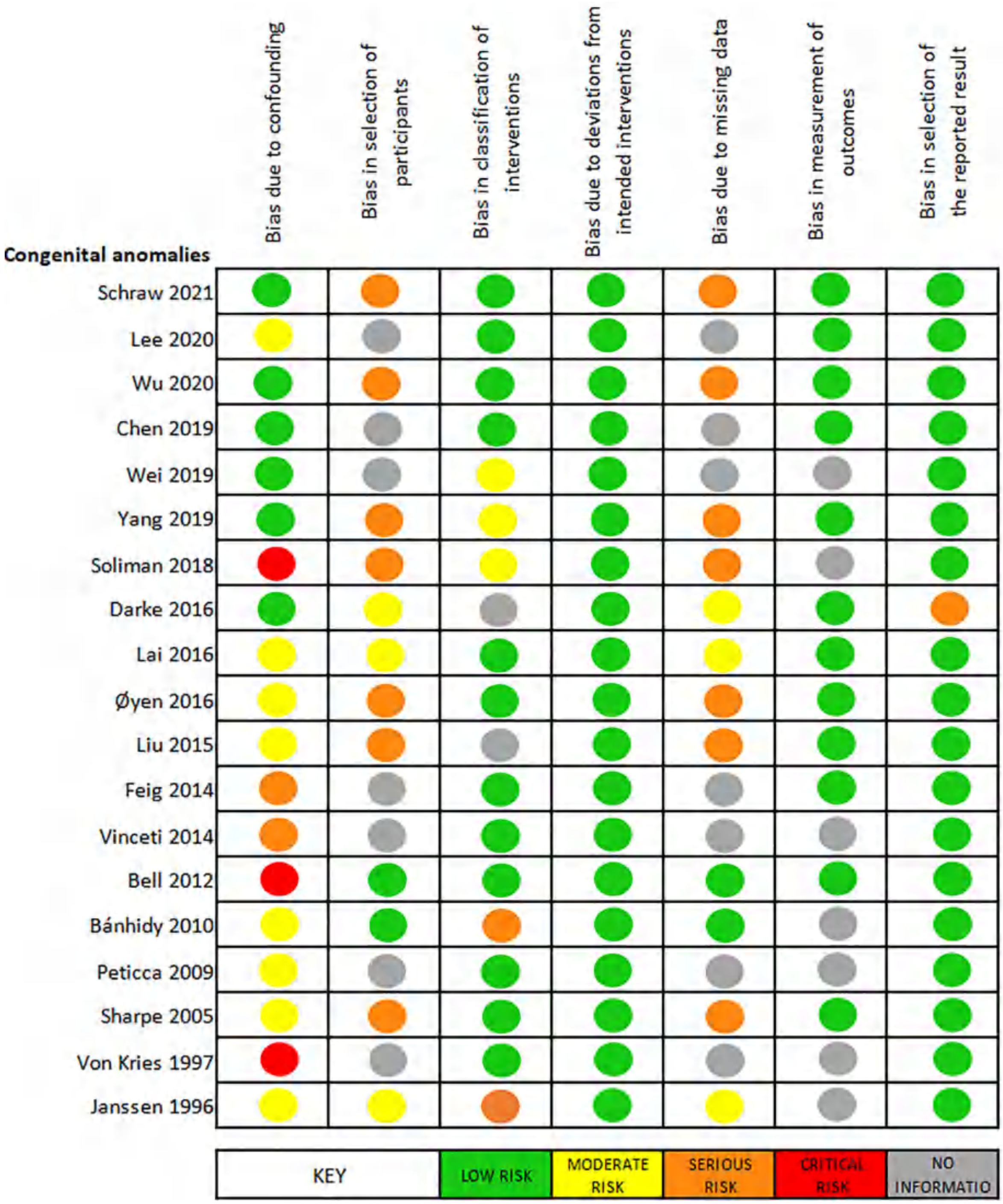

Fig A. Risk of bias summary: effect on congenital anomalies in offspring with pre-gestational diabetes

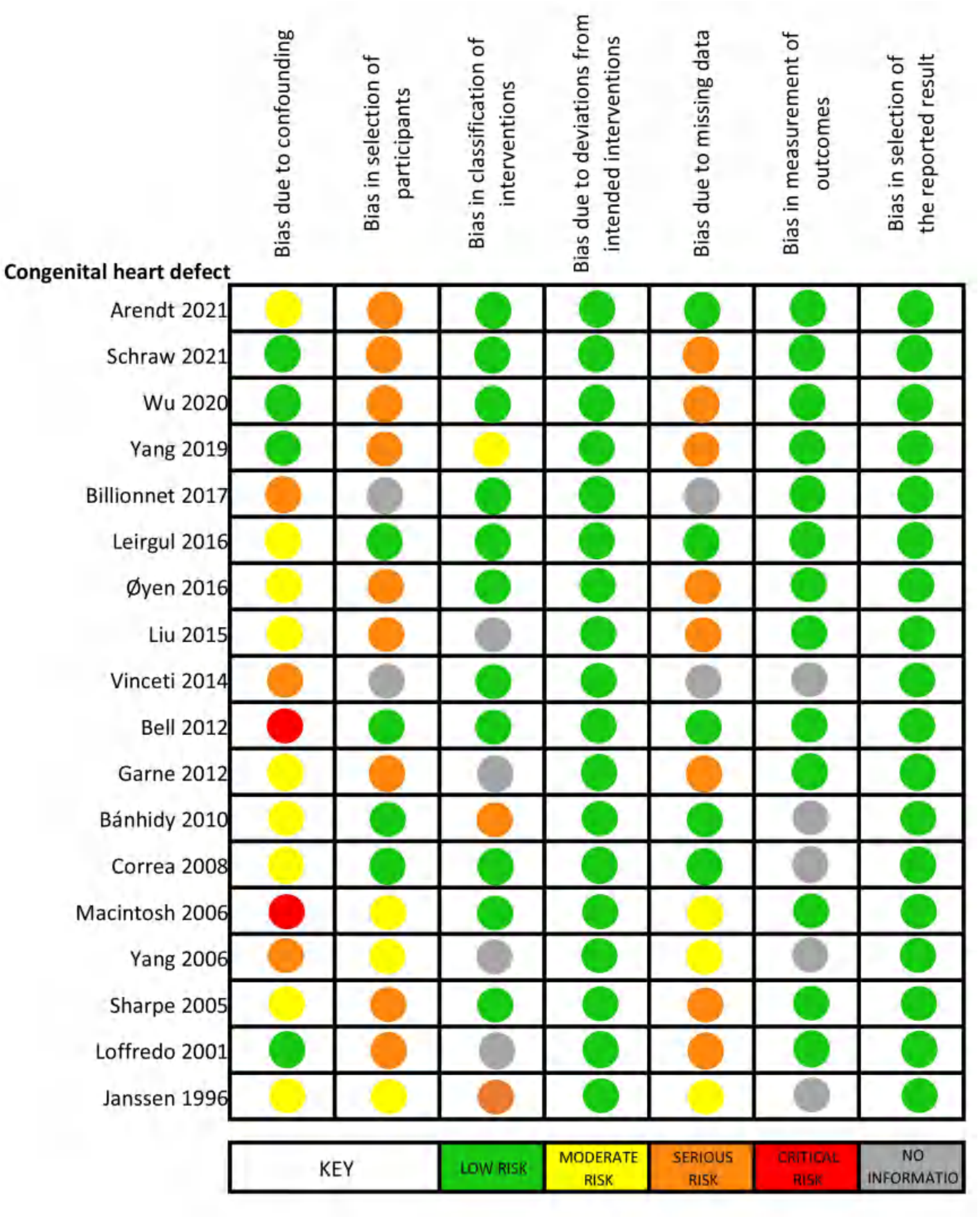

Fig B. Risk of bias summary: effect on congenital heart defects in offspring with pre-gestational diabetes

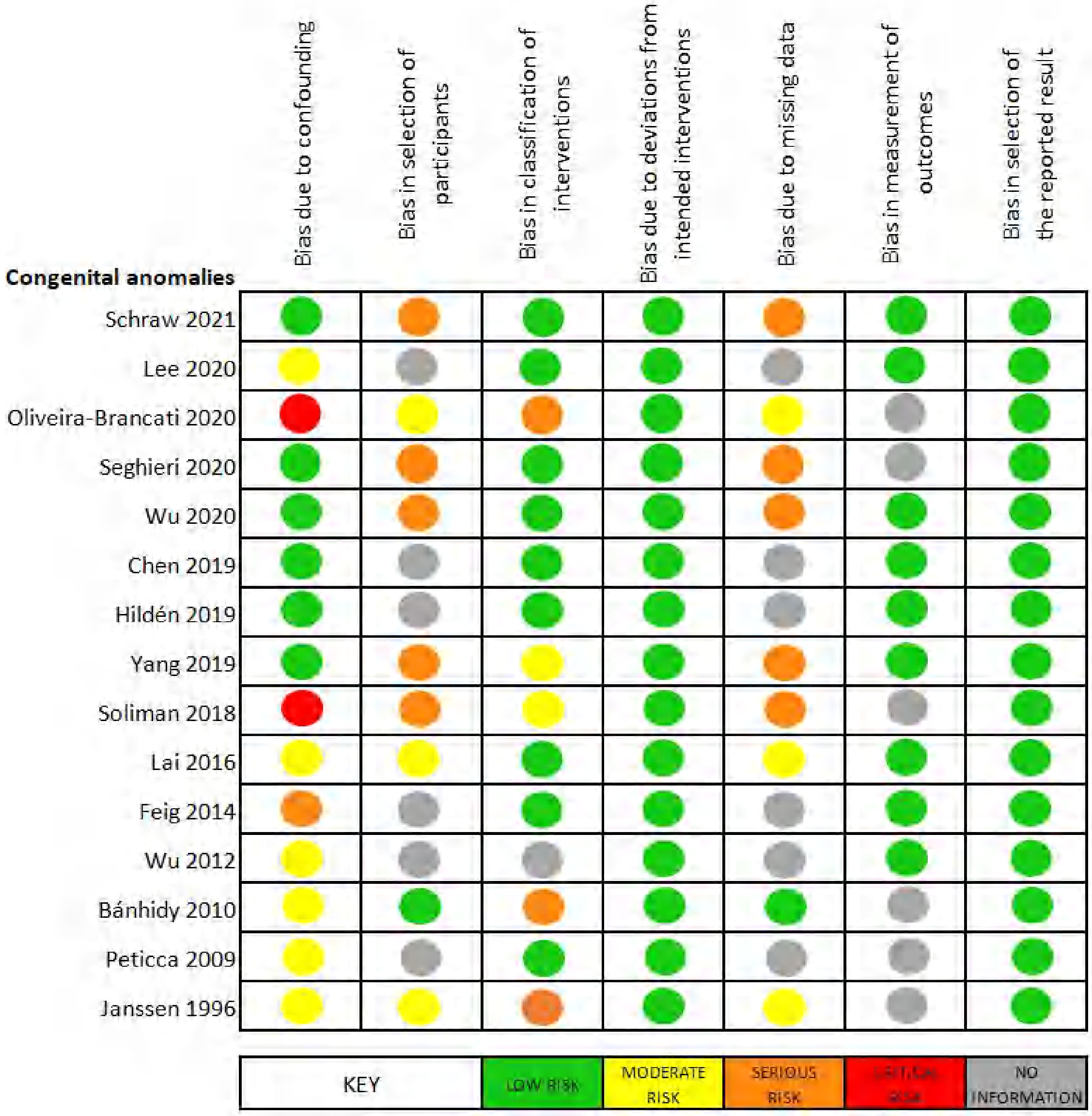

Fig C. Risk of bias summary: effect on congenital anomalies in offspring with gestational diabetes

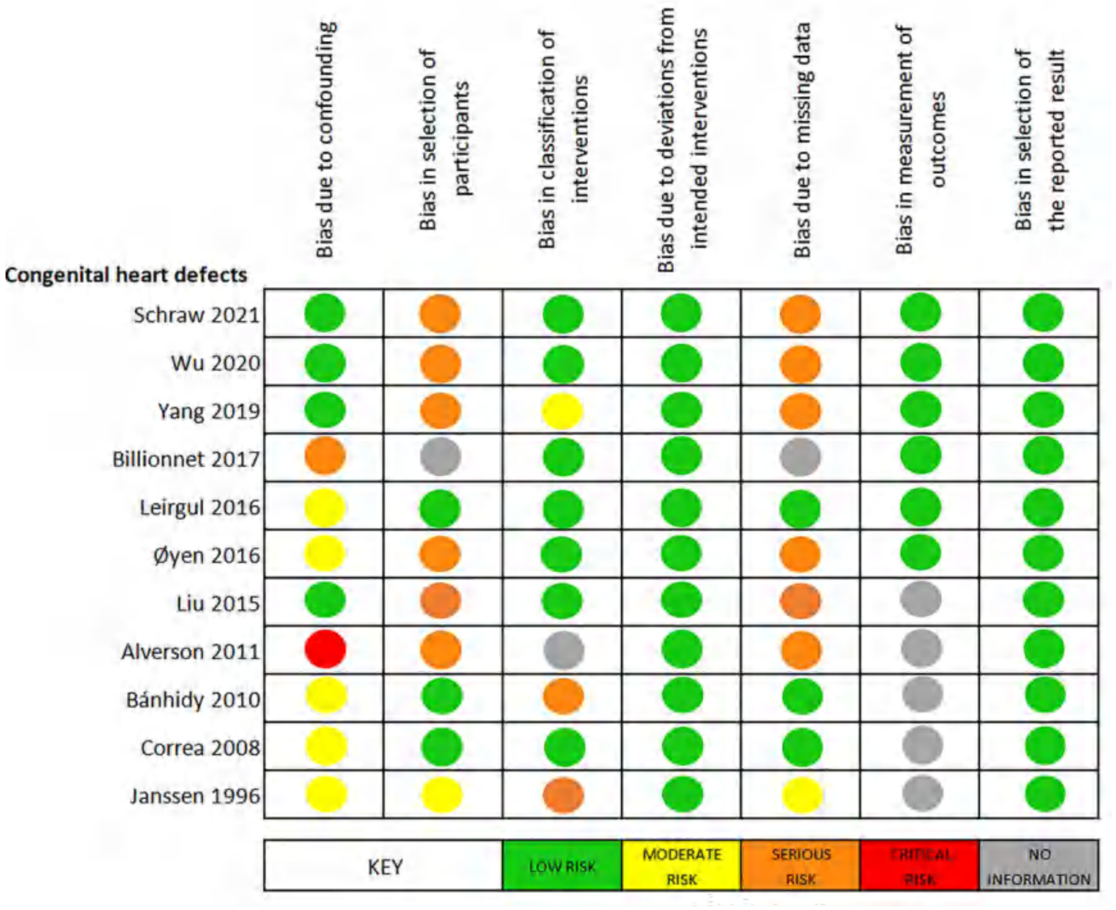

Fig D. Risk of bias summary: effect on congenital heart defects in offspring with gestational diabetes

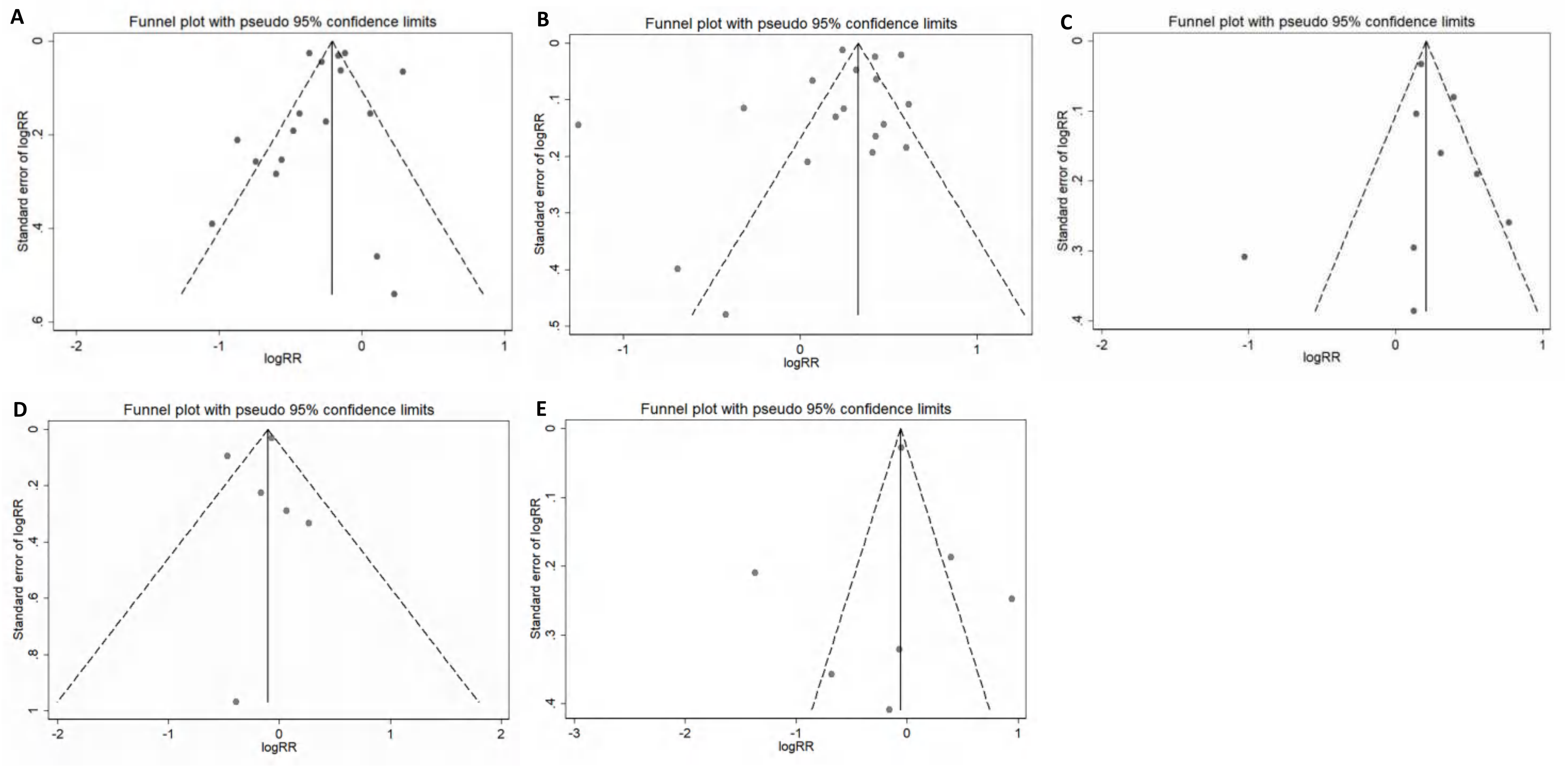

Fig E. Funnel plots of the relative risks of population-based studies on pre-gestational diabetes and the risk of congenital anomalies. A. Overall congenital anomalies (Begg's test *P* = 0.880, Egger's test *P* = 0.304). B. Congenital heart defects (Begg's test *P* = 0.596, Egger's test *P* = 0.845). C. Ventricular septal defects (Begg's test *P* = 1.000, Egger's test *P* = 0.935). D. Congenital anomalies of genitourinary system (Begg's test *P* = 0.951, Egger's test *P* = 0.094). E. Congenital anomalies of musculoskeletal system (Begg's test *P* = 0.640, Egger's test *P* = 0.525). Log RR: natural logarithm of relative risk.

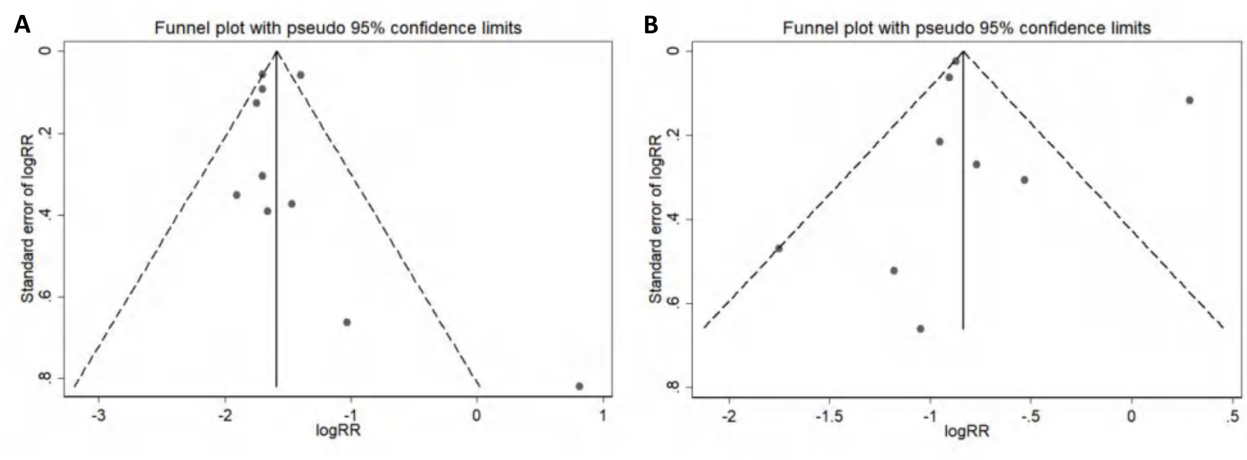

Fig F. Funnel plots of the relative risks of population-based studies on gestational diabetes mellitus and the risk of congenital anomalies. A. Overall congenital anomalies (Begg's test *P* = 0.392, Egger's test *P* = 0.323). B. Congenital heart defects (Begg's test *P* = 0.837, Egger's test *P* = 0.885). Log RR: natural logarithm of relative risk.

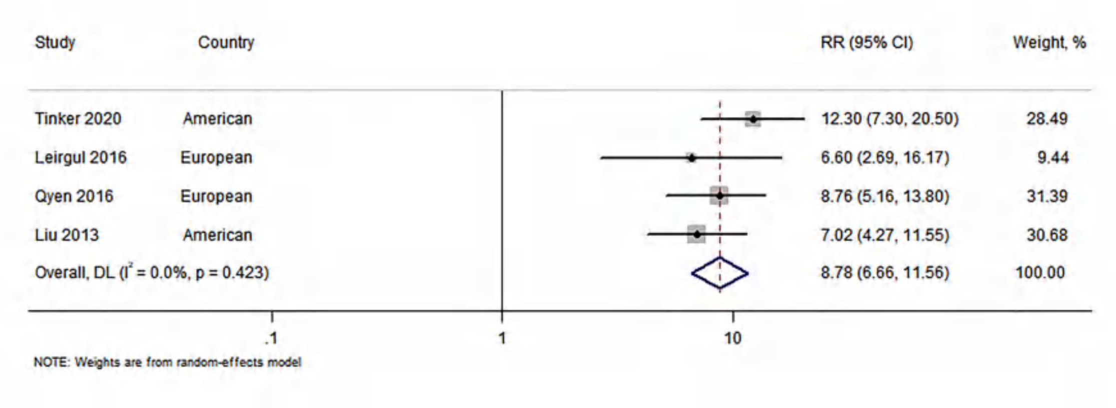

Fig G1. Forest plot of the relative risks of population-based studies on maternal pre-gestational diabetes and the risk of heterotaxia (RR = 8.78, 95% CI, 6.66 to 11.56; I^2 = 0.0%, P = 0.423). DL, DerSimonian and Laird random-effects model; RR, relative risk.

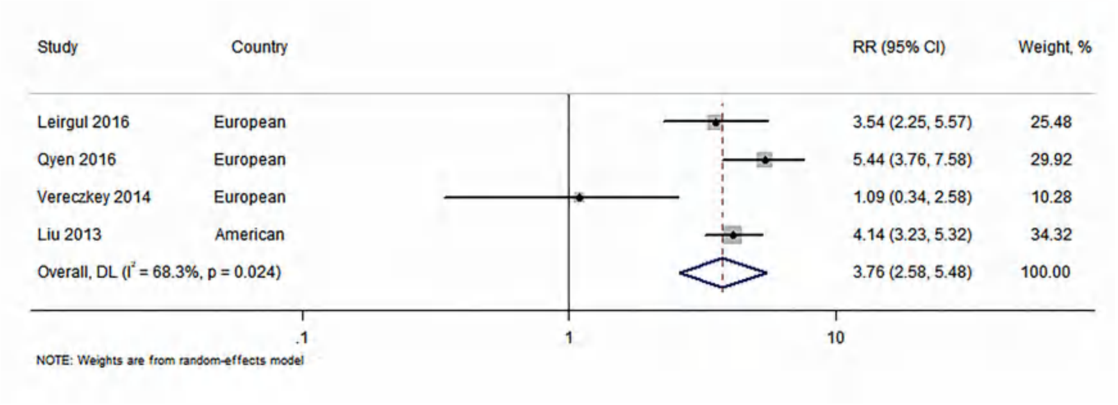

Fig G2. Forest plot of the relative risks of population-based studies on maternal pre-gestational diabetes and the risk of conotruncal defects (RR = 3.76, 95% CI, 2.58 to 5.48; I^2 = 68.3%, P = 0.024). DL, DerSimonian and Laird random-effects model; RR, relative risk.

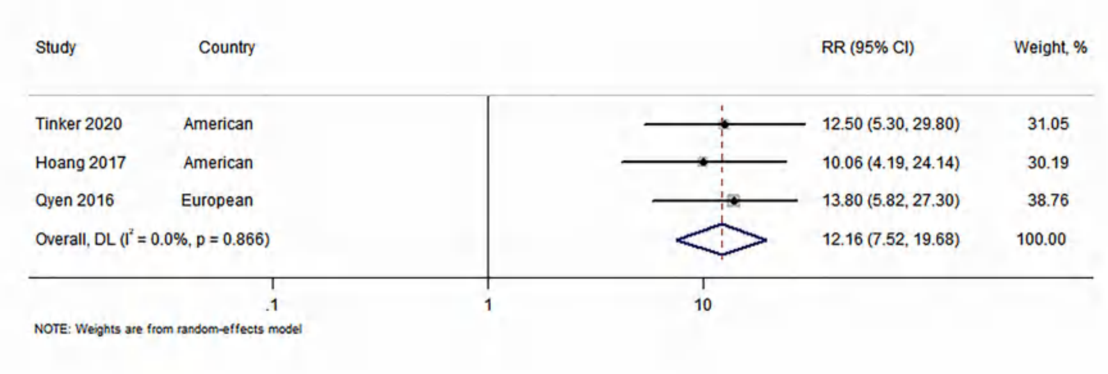

Fig G3. Forest plot of the relative risks of population-based studies on maternal pre-gestational diabetes and the risk of truncus arteriosus (RR = 12.16, 95% CI, 7.52 to 19.68; I^2 = 0.0%, P = 0.866). DL, DerSimonian and Laird random-effects model; RR, relative risk.

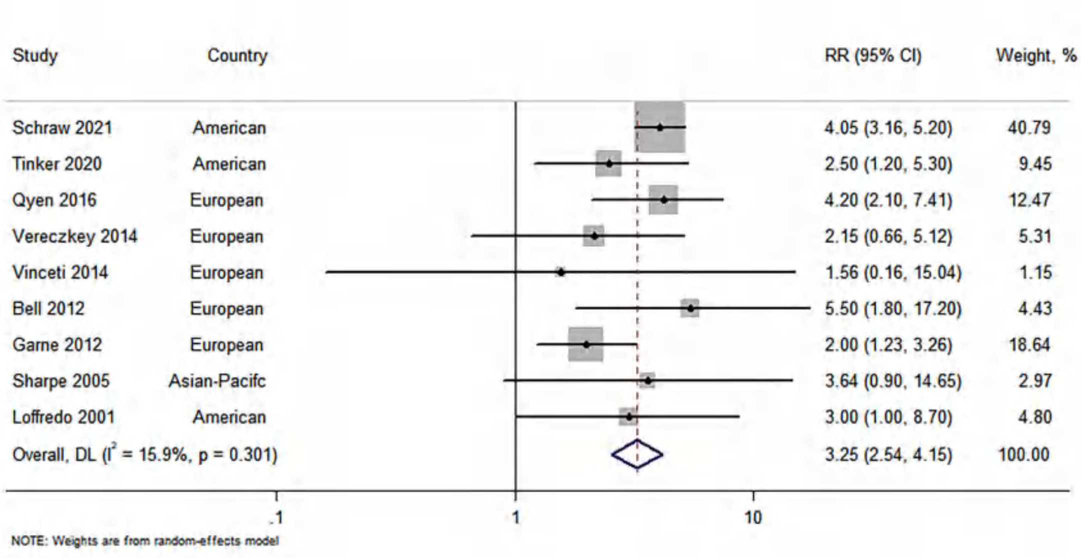

Fig G4. Forest plot of the relative risks of population-based studies on maternal pre-gestational diabetes and the risk of transposition of great vessels (RR = 3.25, 95% CI, 2.54 to 4.15; I^2 = 15.9%, P = 0.301). DL, DerSimonian and Laird random-effects model; RR, relative risk.

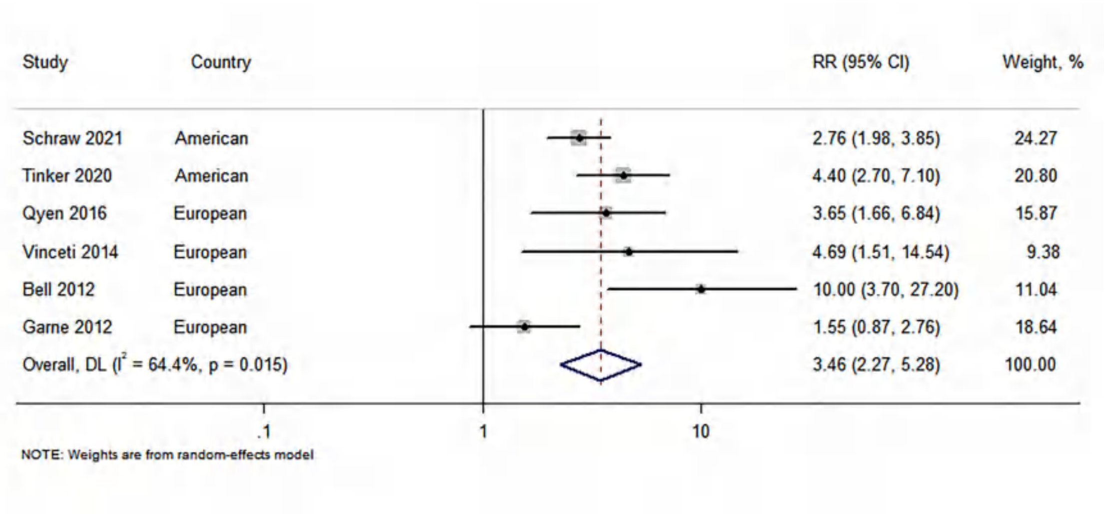

Fig G5. Forest plot of the relative risks of population-based studies on maternal pre-gestational diabetes and the risk of tetralogy of Fallot (RR = 3.46, 95% CI, 2.27 to 5.28; I^2 = 64.4%, P = 0.015). DL, DerSimonian and Laird random-effects model; RR, relative risk.

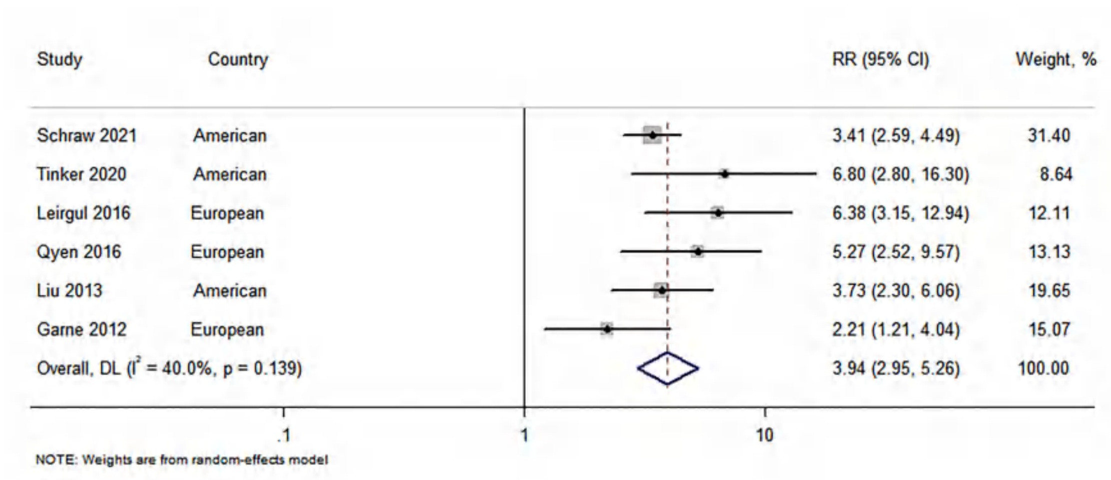

Fig G6. Forest plot of the relative risks of population-based studies on maternal pre-gestational diabetes and the risk of atrioventricular septal defects (RR = 3.94, 95% CI, 2.95 to 5.26; I^2 = 40.0%, P = 0.139). DL, DerSimonian and Laird random-effects model; RR, relative risk.

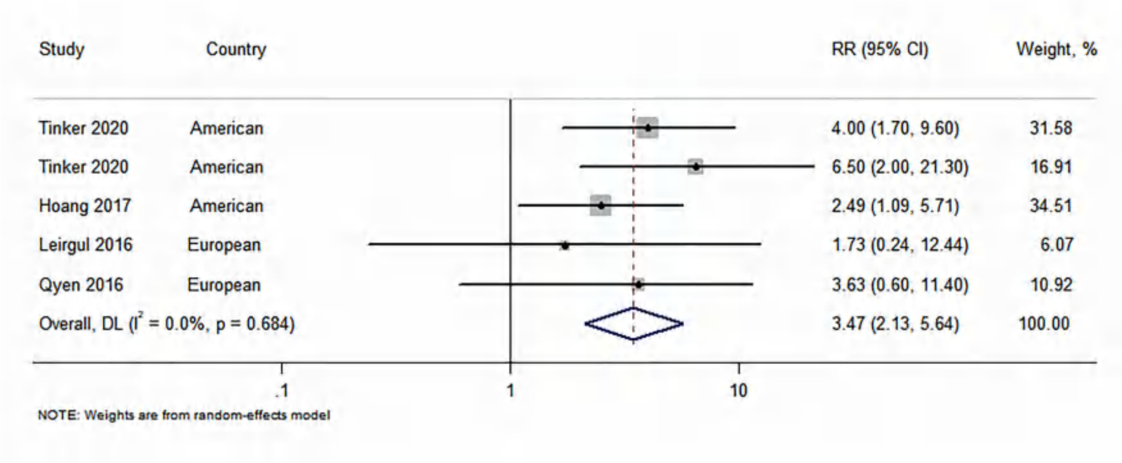

Fig G7. Forest plot of the relative risks of population-based studies on maternal pre-gestational diabetes and the risk of anomalous pulmonary venous return (RR = 3.47, 95% CI, 2.13 to 5.64; I^2 = 0.0%, P = 0.684). DL, DerSimonian and Laird random-effects model; RR, relative risk.

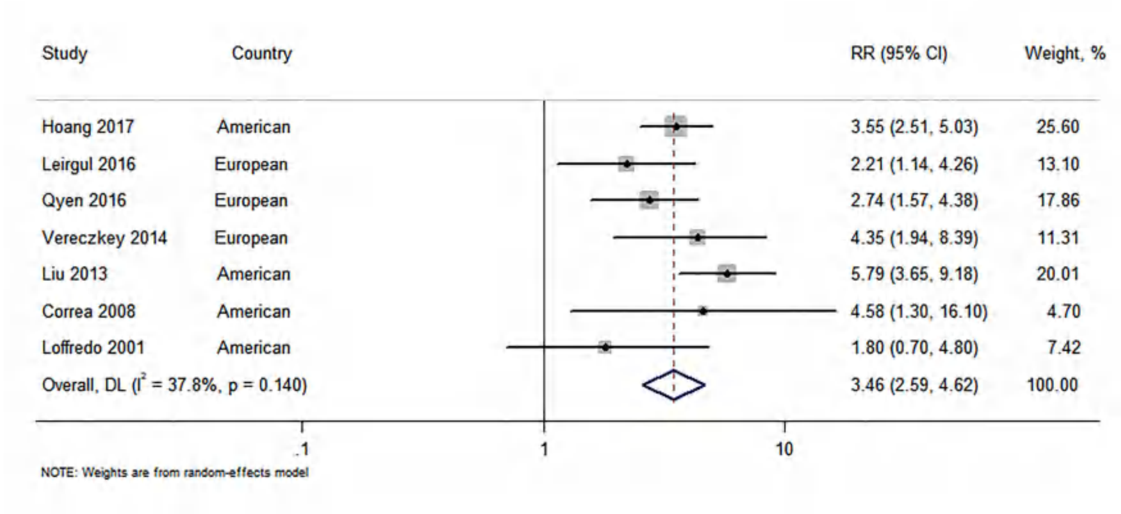

Fig G8. Forest plot of the relative risks of population-based studies on maternal pre-gestational diabetes and the risk of left ventricular outflow tract (RR = 3.46, 95% CI, 2.59 to 4.62; I^2 = 37.8%, P = 0.140). DL, DerSimonian and Laird random-effects model; RR, relative risk.

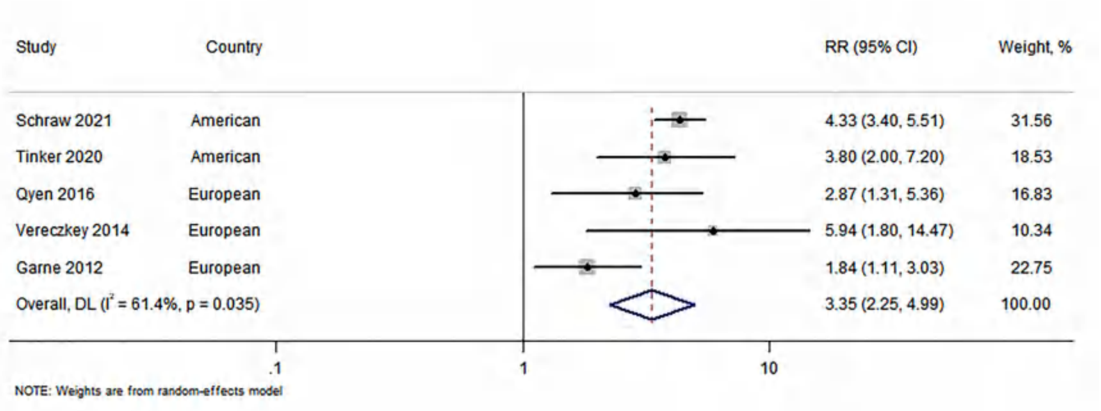

Fig G9. Forest plot of the relative risks of population-based studies on maternal pre-gestational diabetes and the risk of coarctation of aorta (RR = 3.35, 95%CI, 2.25 to 4.99; I^2 = 61.4%, P = 0.035). DL, DerSimonian and Laird random-effects model; RR, relative risk.

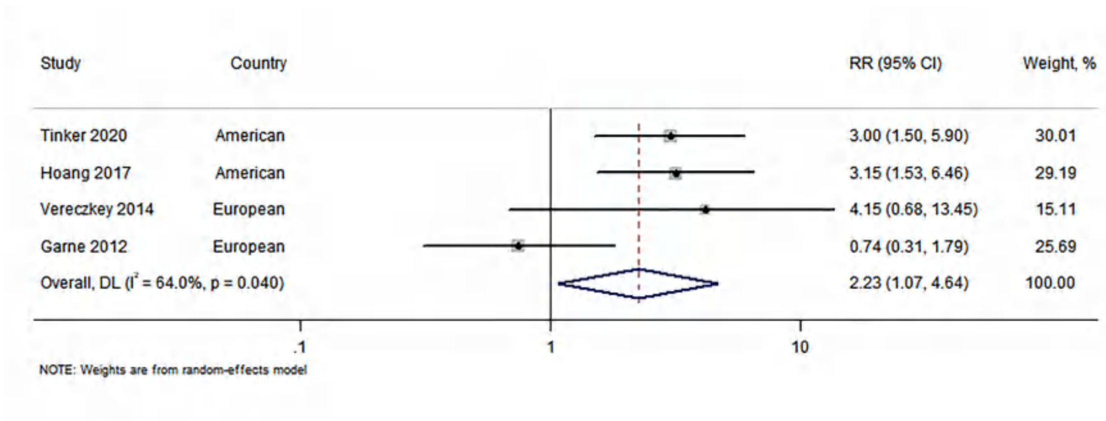

Fig G10. Forest plot of the relative risks of population-based studies on maternal pre-gestational diabetes and the risk of hypoplastic left heart (RR = 2.23, 95% CI, 1.07 to 4.64; I^2 = 64.0%, P = 0.040). DL, DerSimonian and Laird random-effects model; RR, relative risk.

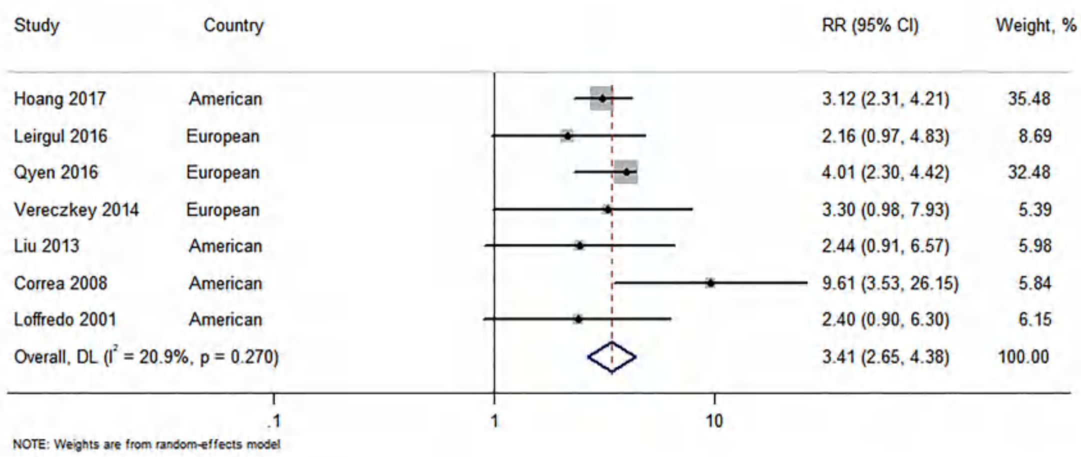

Fig G11. Forest plot of the relative risks of population-based studies on maternal pre-gestational diabetes and the risk of right ventricular outflow tract (RR = 3.41, 95% CI, 2.65 to 4.38; I^2 = 20.9%, P = 0.270). DL, DerSimonian and Laird random-effects model; RR, relative risk.

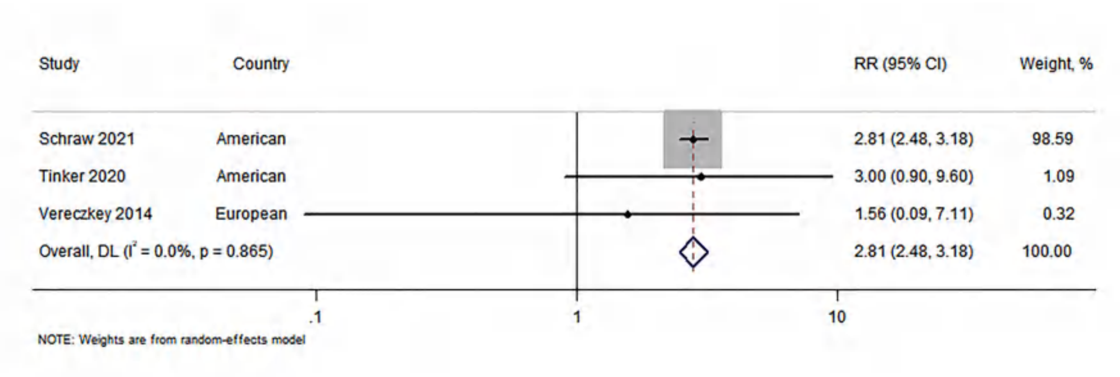

Fig G12. Forest plot of the relative risks of population-based studies on maternal pre-gestational diabetes and the risk of pulmonary artery anomalies (RR = 2.81, 95% CI, 2.48 to 3.18; I^2 = 0.0%, P = 0.865). DL, DerSimonian and Laird random-effects model; RR, relative risk.

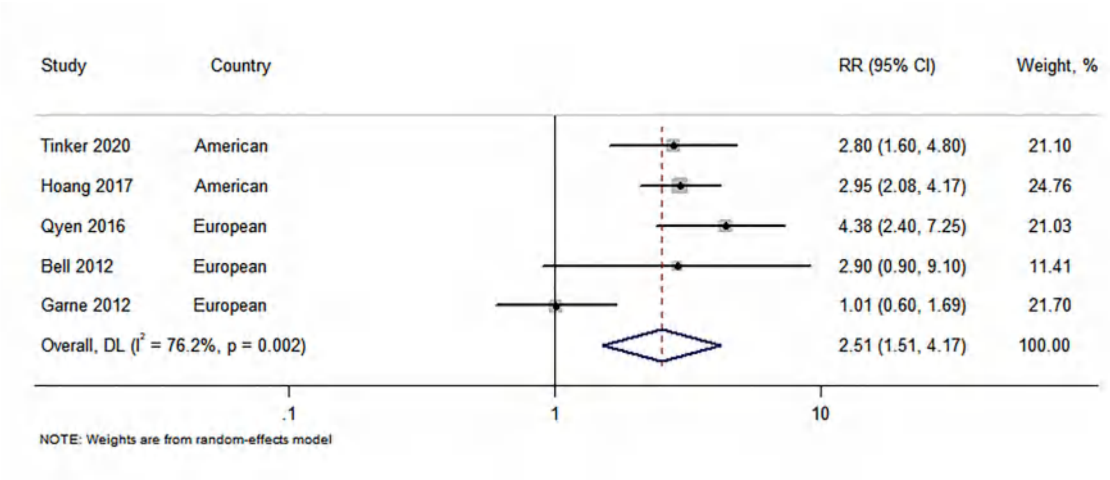

Fig G13. Forest plot of the relative risks of population-based studies on maternal pre-gestational diabetes and the risk of pulmonary valve stenosis (RR = 2.51, 95% CI, 1.51 to 4.17; I^2 = 76.2%, P = 0.002). DL, DerSimonian and Laird random-effects model; RR, relative risk.

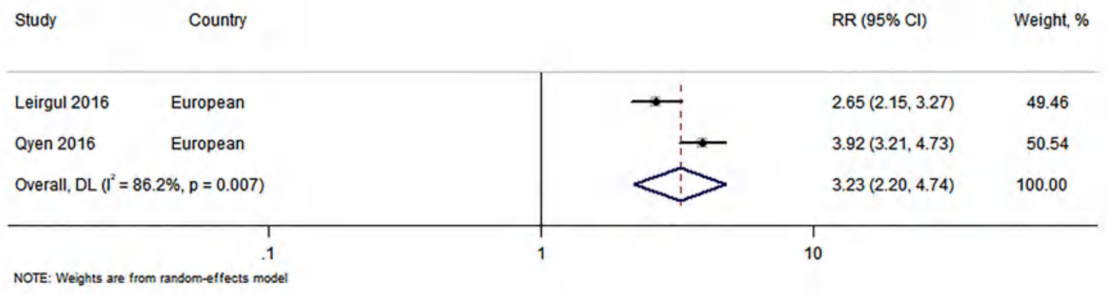

Fig G14. Forest plot of the relative risks of population-based studies on maternal pre-gestational diabetes and the risk of septal defects (RR = 3.23, 95% CI, 2.20 to 4.74; I^2 = 86.2%, P = 0.007). DL, DerSimonian and Laird random-effects model; RR, relative risk.

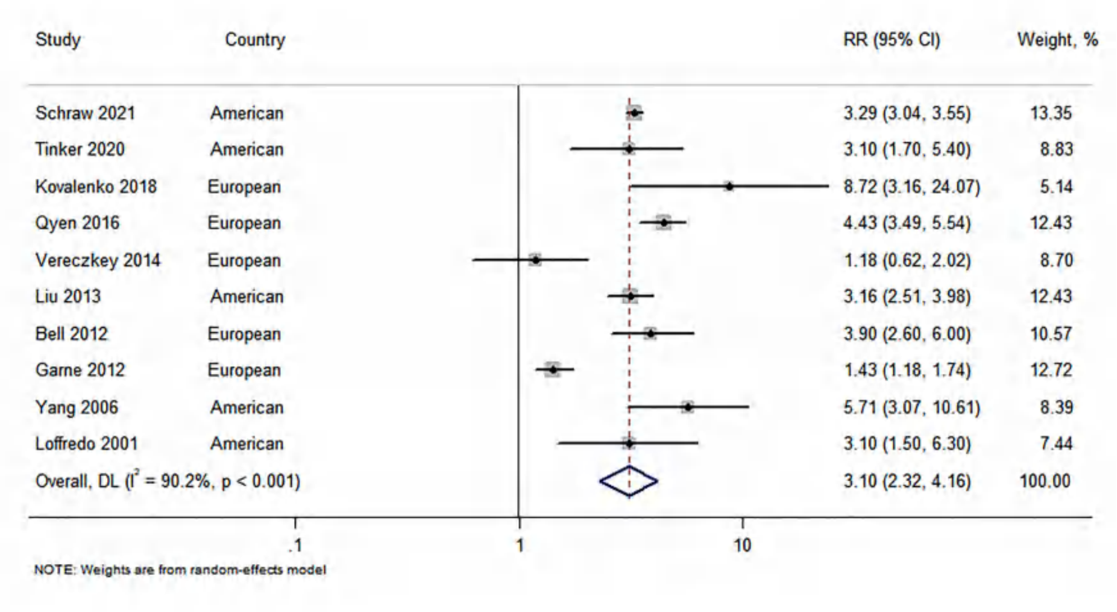

Fig G15. Forest plot of the relative risks of population-based studies on maternal pre-gestational diabetes and the risk of ventricular septal defects (RR = 3.10, 95% CI, 2.32 to 4.16; I^2 = 90.2%, P < 0.001). DL, DerSimonian and Laird random-effects model; RR, relative risk.

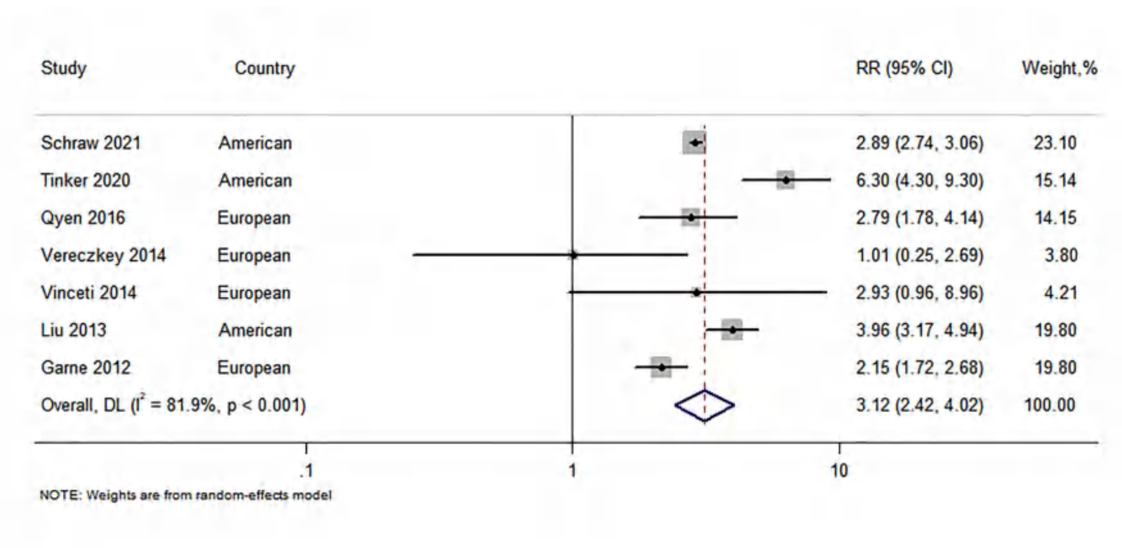

Fig G16. Forest plot of the relative risks of population-based studies on maternal pre-gestational diabetes and the risk of atrial septal defects (RR = 3.12, 95% CI, 2.42 to 4.02; I^2 = 81.9%, P < 0.001). DL, DerSimonian and Laird random-effects model; RR, relative risk.

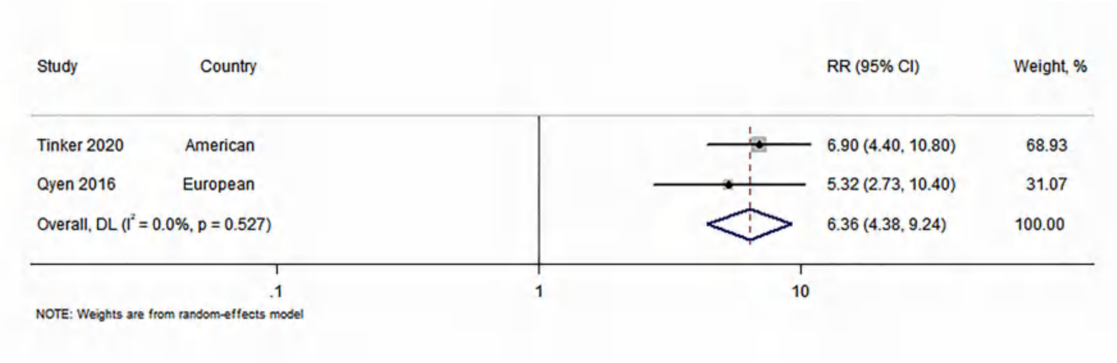

Fig G17. Forest plot of the relative risks of population-based studies on maternal pre-gestational diabetes and the risk of ventricular septal defect and atrial septal defects (RR = 6.36, 95% CI, 4.38 to 9.24; I^2 = 0.0%, P = 0.527). DL, DerSimonian and Laird random-effects model; RR, relative risk.

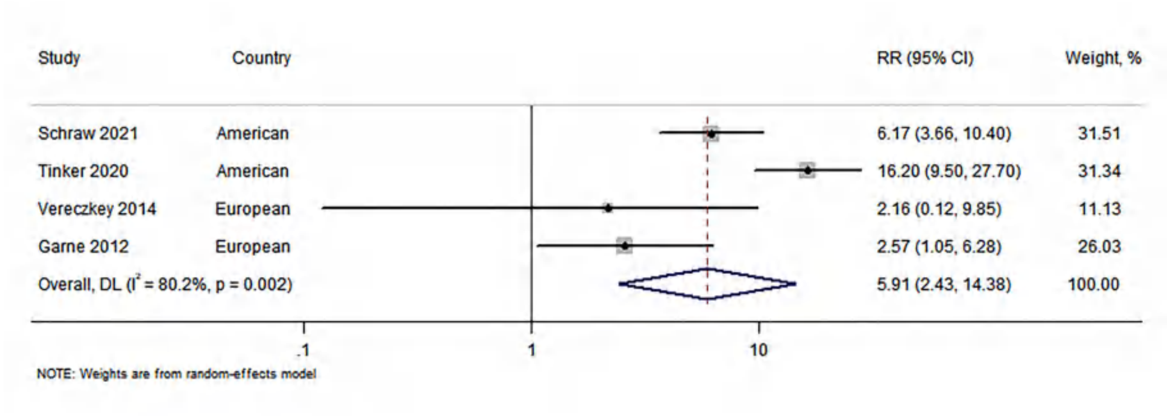

Fig G18. Forest plot of the relative risks of population-based studies on maternal pre-gestational diabetes and the risk of single ventricle (RR = 5.91, 95% CI, 2.43 to 14.38; I^2 = 80.2%, P = 0.002). DL, DerSimonian and Laird random-effects model; RR, relative risk.

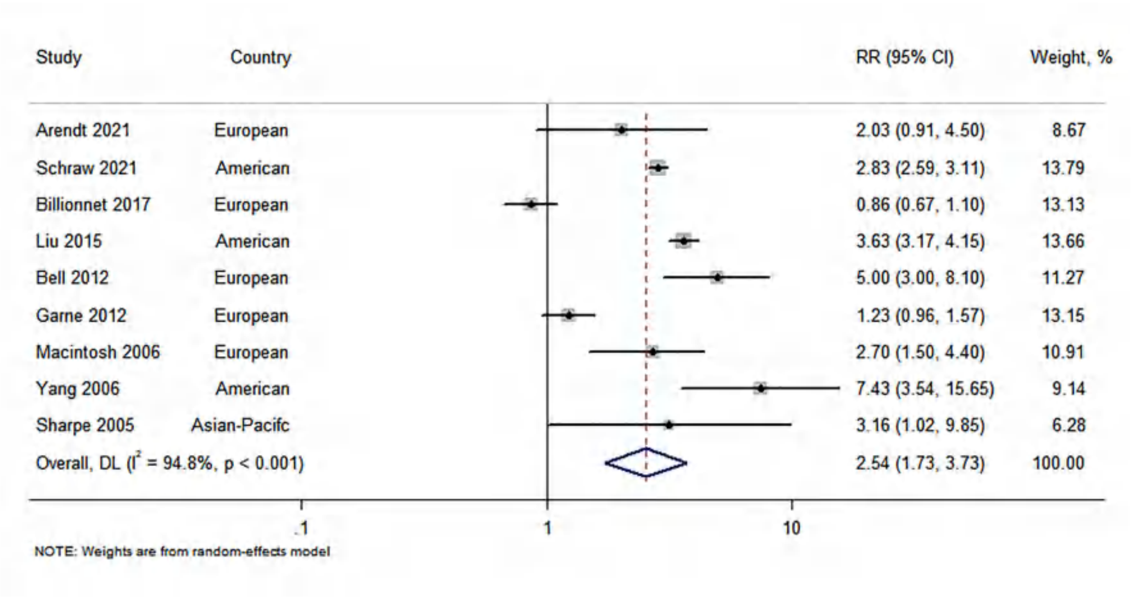

Fig H1. Forest plot of the relative risks of population-based studies on maternal pre-gestational diabetes and the risk of congenital anomalies of nervous system (RR = 2.54, 95% CI, 1.73 to 3.73; I^2 = 94.8%, P < 0.001). DL, DerSimonian and Laird random-effects model; RR, relative risk.

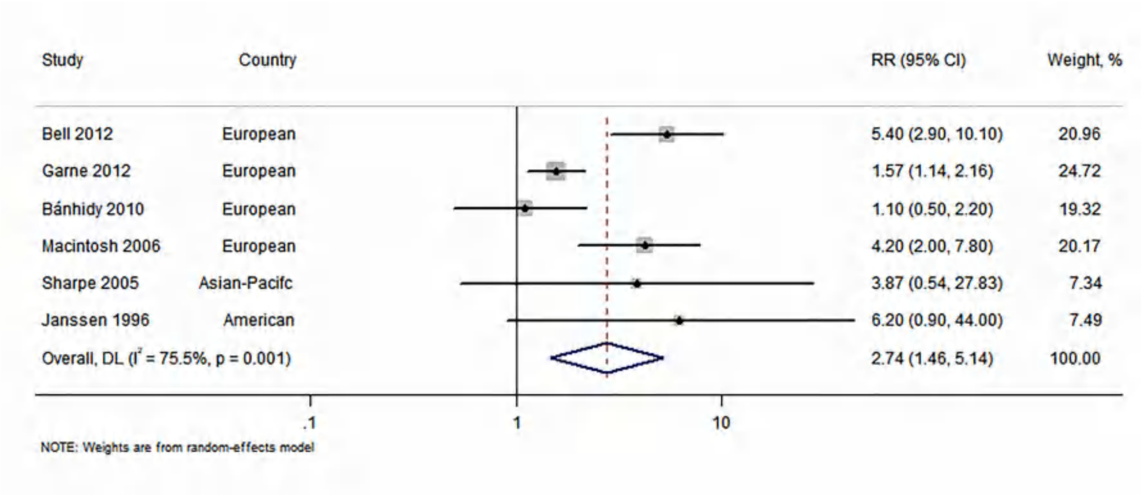

Fig H2. Forest plot of the relative risks of population-based studies on maternal pre-gestational diabetes and the risk of neural tube defects (RR = 2.74, 95% CI, 1.46 to 5.14; I^2 = 75.5%, P = 0.001). DL, DerSimonian and Laird random-effects model; RR, relative risk.

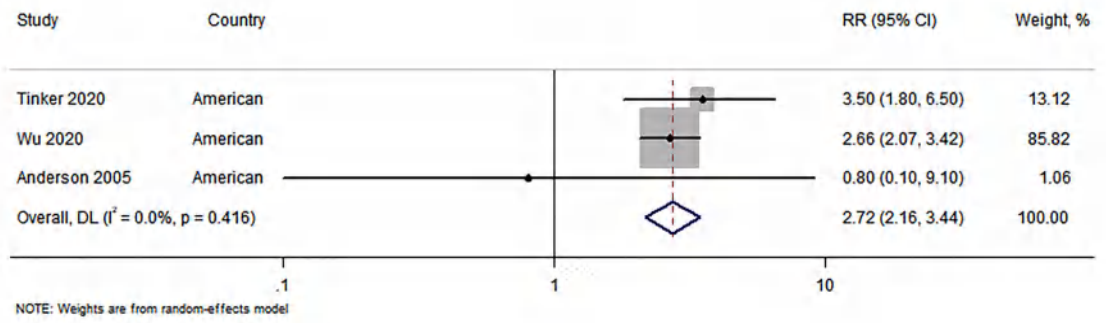

Fig H3. Forest plot of the relative risks of population-based studies on maternal pre-gestational diabetes and the risk of anencephaly (RR = 2.72, 95% CI, 2.16 to 3.44; I^2 = 0.0%, P = 0.416). DL, DerSimonian and Laird random-effects model; RR, relative risk.

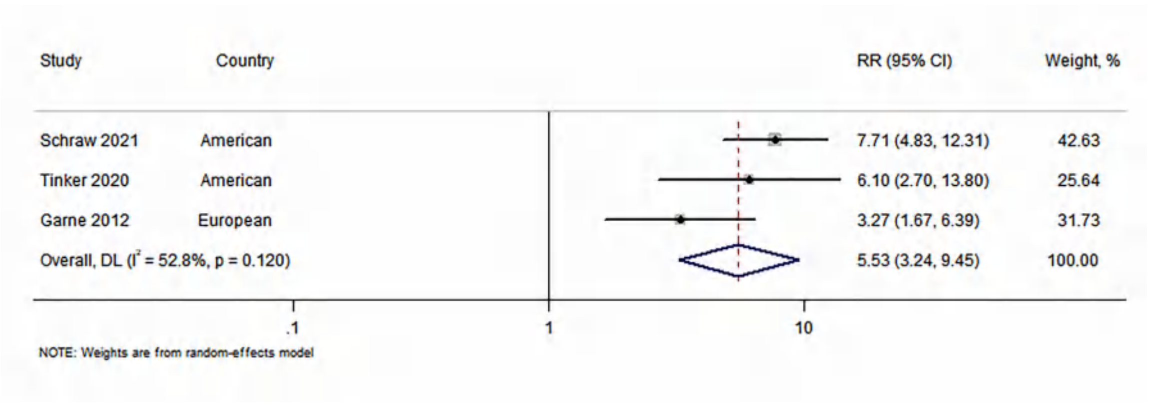

Fig H4. Forest plot of the relative risks of population-based studies on maternal pre-gestational diabetes and the risk of encephalocele (RR = 5.53, 95% CI, 3.24 to 9.45; I^2 = 52.8%, P = 0.120). DL, DerSimonian and Laird random-effects model; RR, relative risk.

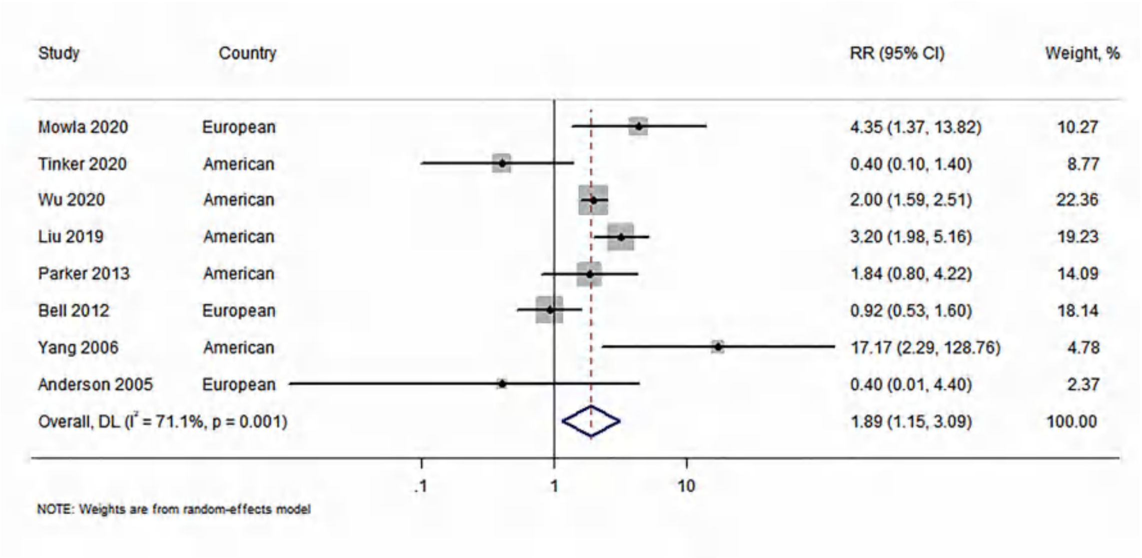

Fig H5. Forest plot of the relative risks of population-based studies on maternal pre-gestational diabetes and the risk of spina bifida (RR = 1.89, 95% CI, 1.15 to 3.09; I^2 = 71.1%, P = 0.001). DL, DerSimonian and Laird random-effects model; RR, relative risk.

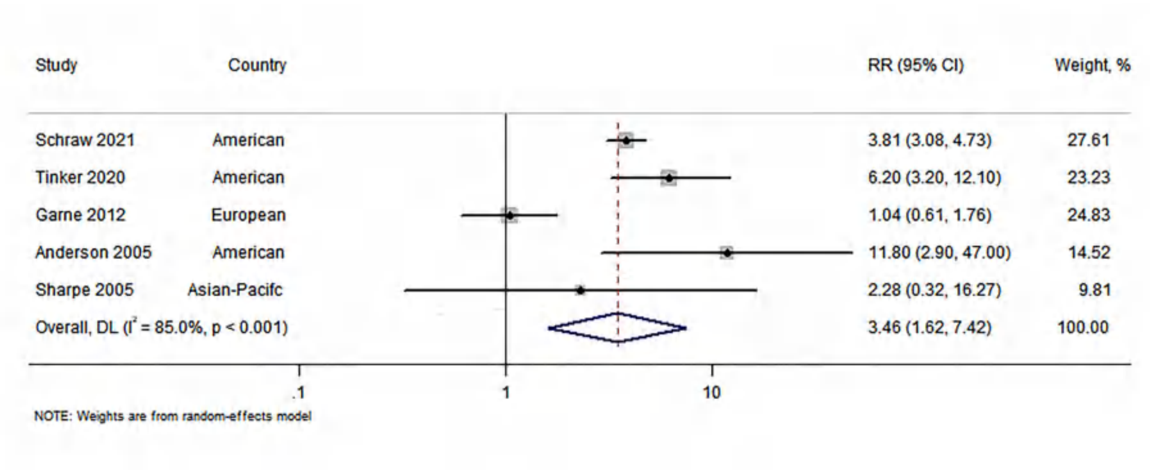

Fig H6. Forest plot of the relative risks of population-based studies on maternal pre-gestational diabetes and the risk of hydrocephaly (RR = 3.46, 95% CI, 1.62 to 7.42; I^2 = 85.0%, P < 0.001). DL, DerSimonian and Laird random-effects model; RR, relative risk.

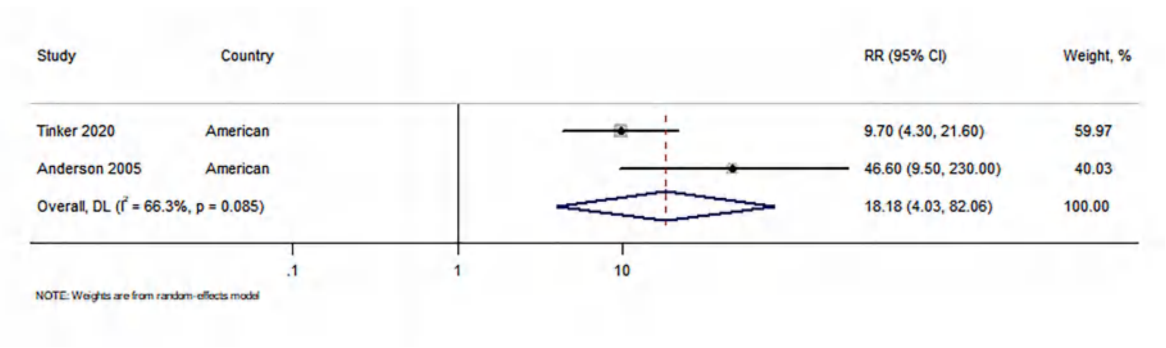

Fig H7. Forest plot of the relative risks of population-based studies on maternal pre-gestational diabetes and the risk of holoprosencephaly (RR = 18.18, 95% CI, 4.03 to 82.06; I^2 = 66.3%, P = 0.085). DL, DerSimonian and Laird random-effects model; RR, relative risk.

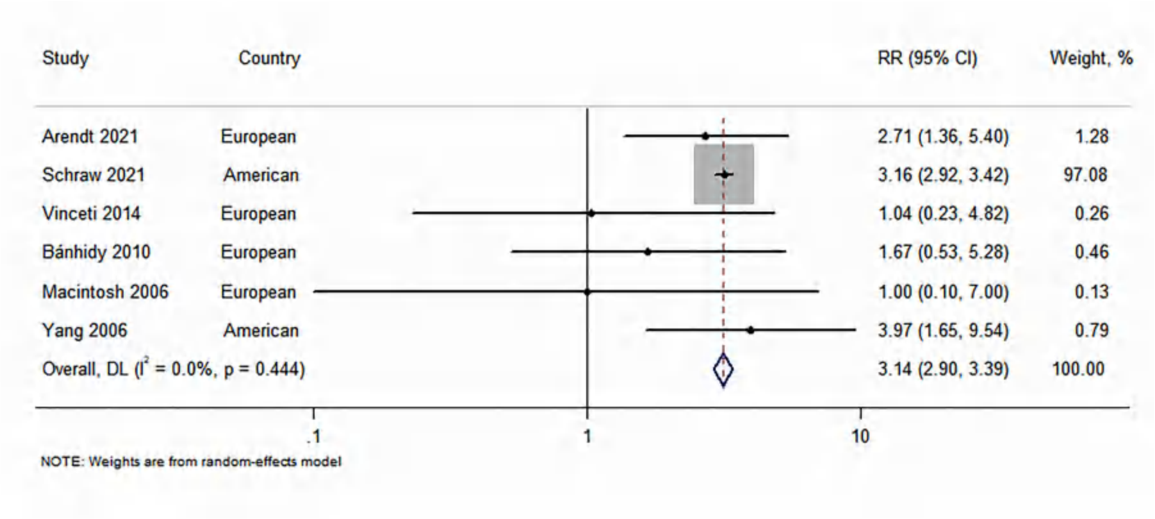

Fig H8. Forest plot of the relative risks of population-based studies on maternal pre-gestational diabetes and the risk of eye, ear, face, and neck (RR = 3.14, 95% CI, 2.90 to 3.39; I^2 = 0.0%, P = 0.444). DL, DerSimonian and Laird random-effects model; RR, relative risk.

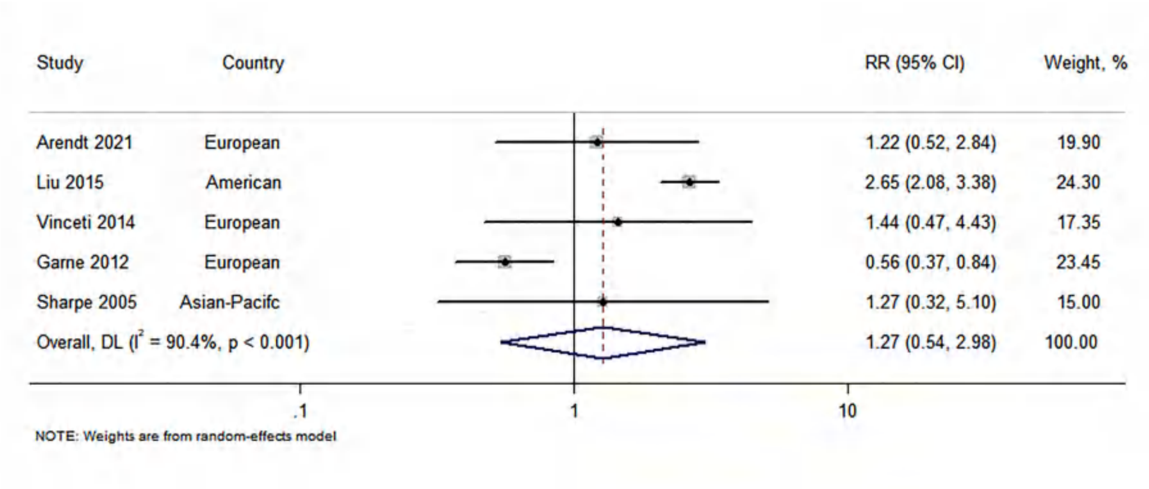

Fig H9. Forest plot of the relative risks of population-based studies on maternal pre-gestational diabetes and the risk of orofacial clefts (RR = 1.27, 95% CI, 0.54 to 2.98; I^2 = 90.4%, P < 0.001). DL, DerSimonian and Laird random-effects model; RR, relative risk.

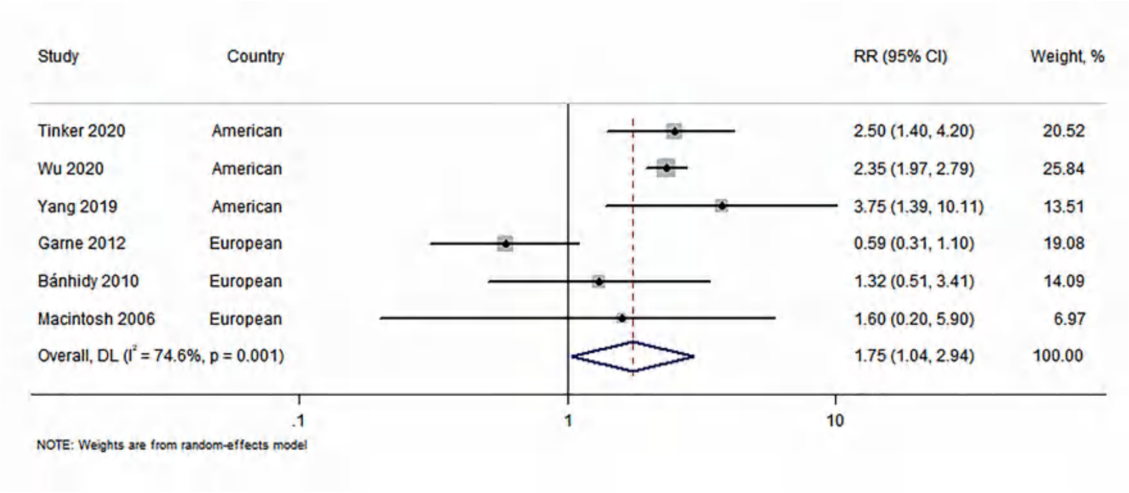

Fig H10. Forest plot of the relative risks of population-based studies on maternal pre-gestational diabetes and the risk of cleft palate (RR = 1.75, 95% CI, 1.04 to 2.94; I^2 = 74.6%, P = 0.001). DL, DerSimonian and Laird random-effects model; RR, relative risk.

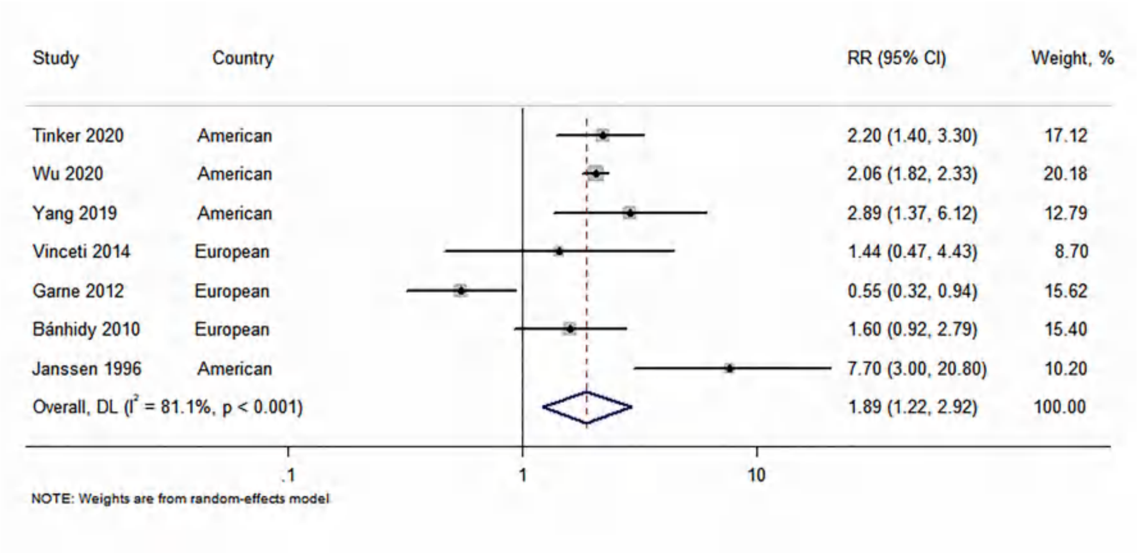

Fig H11. Forest plot of the relative risks of population-based studies on maternal pre-gestational diabetes and the risk of cleft lip with or without palate (RR = 1.89, 95% CI, 1.22 to 2.92; I^2 = 81.1%, P < 0.001). DL, DerSimonian and Laird random-effects model; RR, relative risk.

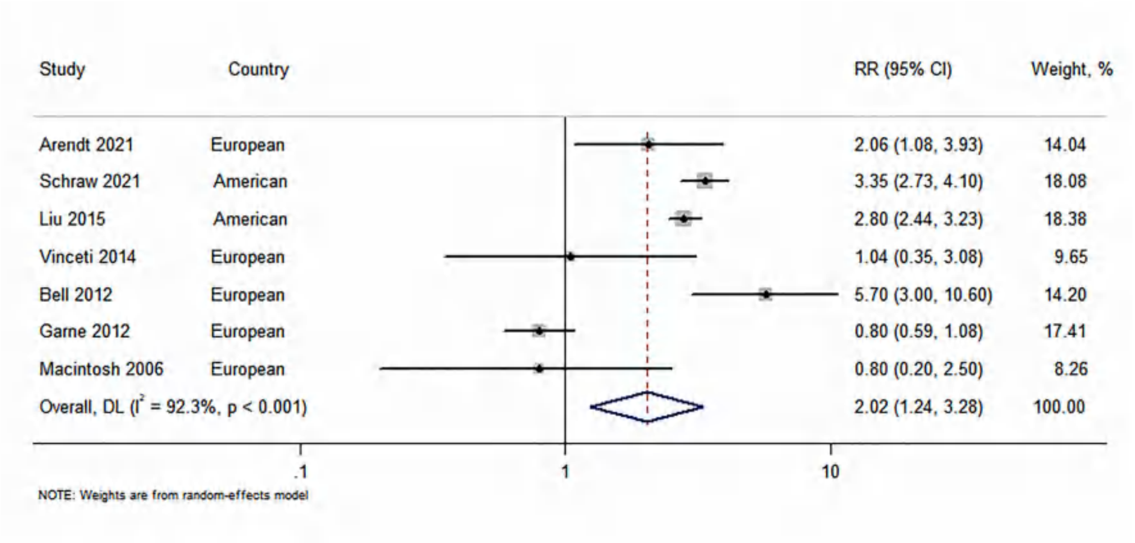

Fig H12. Forest plot of the relative risks of population-based studies on maternal pre-gestational diabetes and the risk of congenital anomalies of digestive system (RR = 2.02, 95% CI, 1.24 to 3.28; I^2 = 92.3%, P < 0.001). DL, DerSimonian and Laird random-effects model; RR, relative risk.

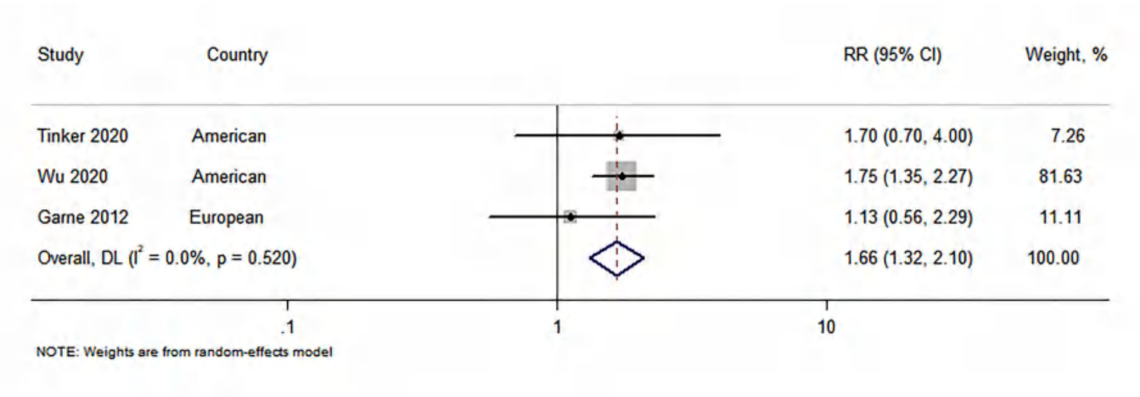

Fig H13. Forest plot of the relative risks of population-based studies on maternal pre-gestational diabetes and the risk of diaphragmatic hernia (RR = 1.66, 95% CI, 1.32 to 2.10; I^2 = 0.0%, P = 0.520). DL, DerSimonian and Laird random-effects model; RR, relative risk.

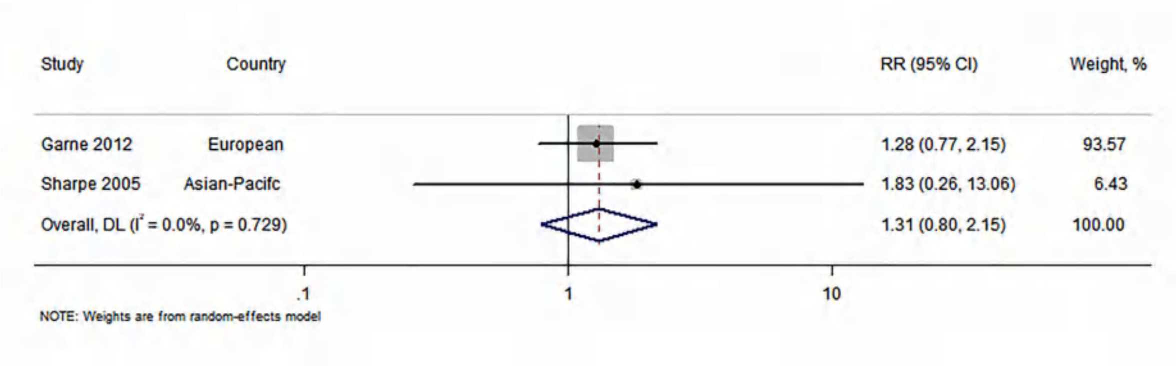

Fig H14. Forest plot of the relative risks of population-based studies on maternal pre-gestational diabetes and the risk of abdominal wall defects (RR = 1.31, 95% CI, 0.80 to 2.15; I^2 = 0.0%, P = 0.729). DL, DerSimonian and Laird random-effects model; RR, relative risk.

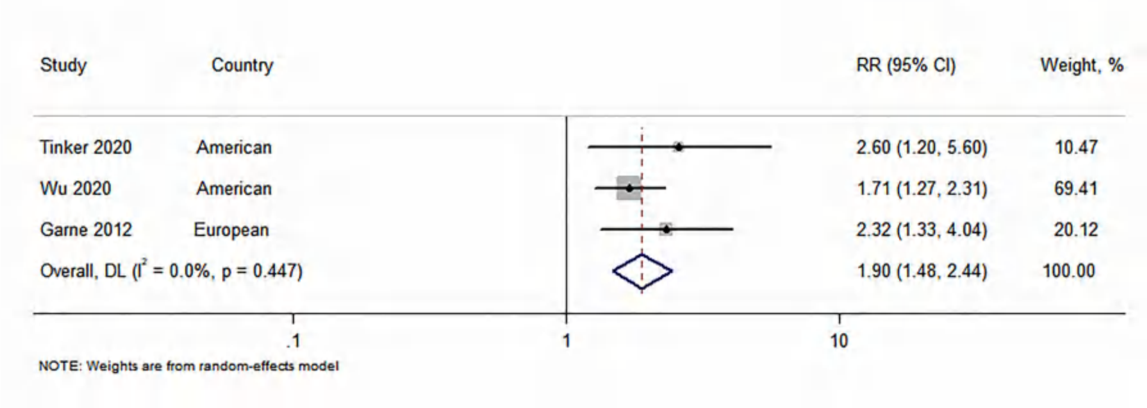

Fig H15. Forest plot of the relative risks of population-based studies on maternal pre-gestational diabetes and the risk of omphalocele (RR = 1.90, 95% CI, 1.48 to 2.44; I^2 = 0.0%, P = 0.447). DL, DerSimonian and Laird random-effects model; RR, relative risk.

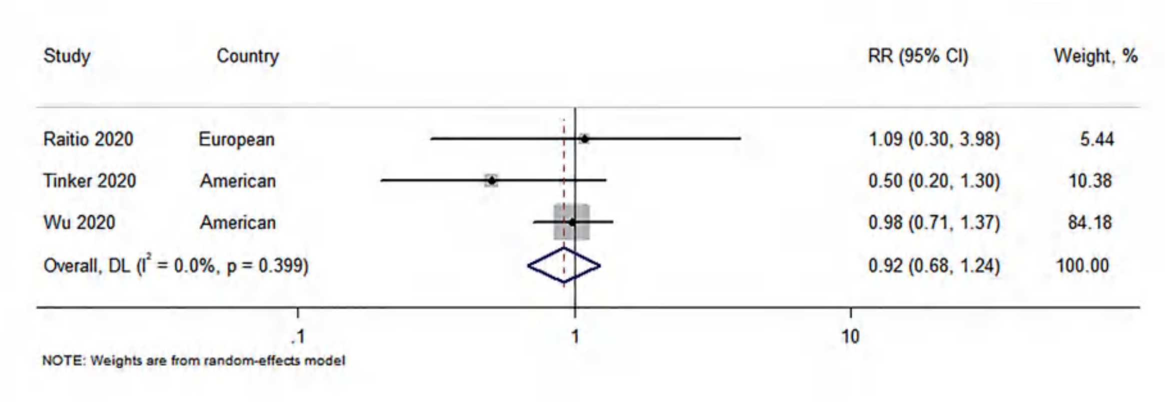

Fig H16. Forest plot of the relative risks of population-based studies on maternal pre-gestational diabetes and the risk of gastroschisis (RR = 0.92, 95% CI, 0.68 to 1.24; I^2 = 0.0%, P = 0.399). DL, DerSimonian and Laird random-effects model; RR, relative risk.

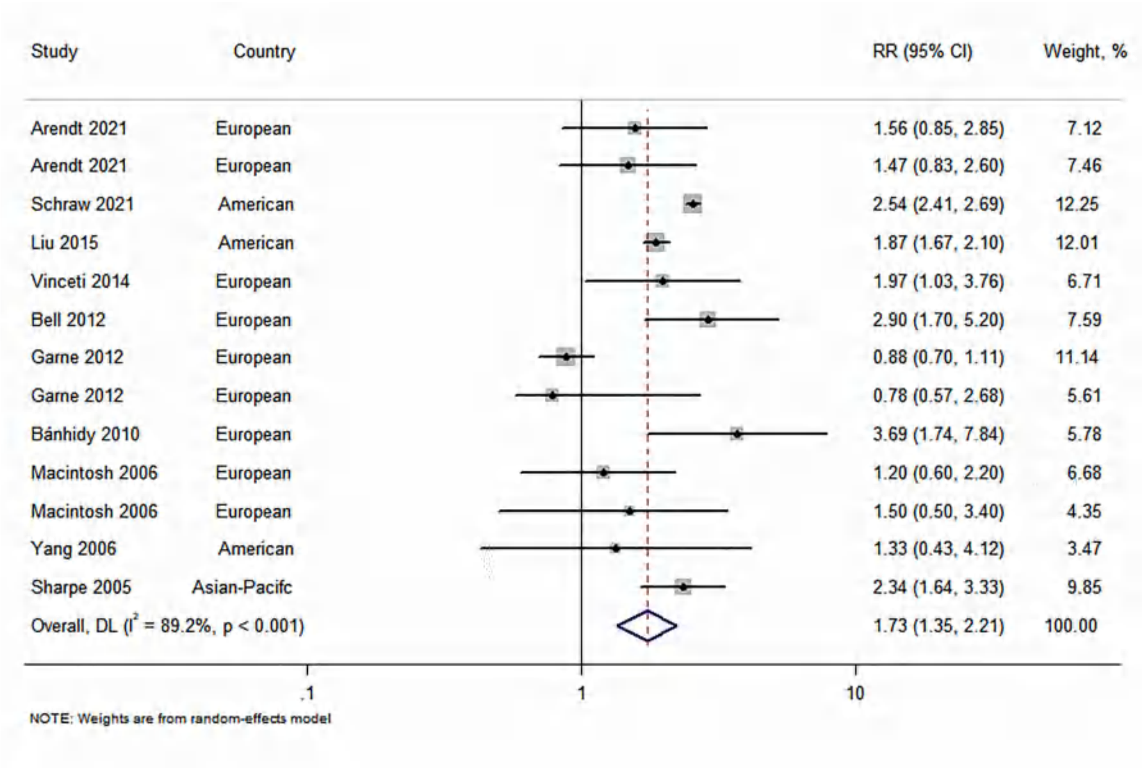

Fig H17. Forest plot of the relative risks of population-based studies on maternal pre-gestational diabetes and the risk of congenital anomalies of genitourinary system (RR = 1.73, 95% CI, 1.35 to 2.21; I^2 = 89.2%, P < 0.001). DL, DerSimonian and Laird random-effects model; RR, relative risk.

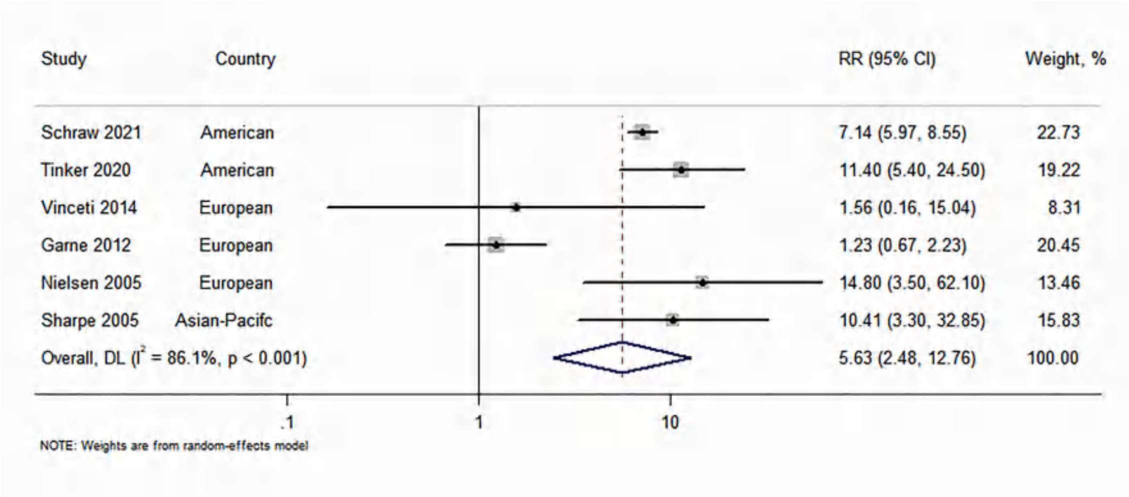

Fig H18. Forest plot of the relative risks of population-based studies on maternal pre-gestational diabetes and the risk of renal agenesis/dysgenesis (RR = 5.63, 95% CI, 2.48 to 12.76; I^2 = 86.1%, P < 0.001). DL, DerSimonian and Laird random-effects model; RR, relative risk.

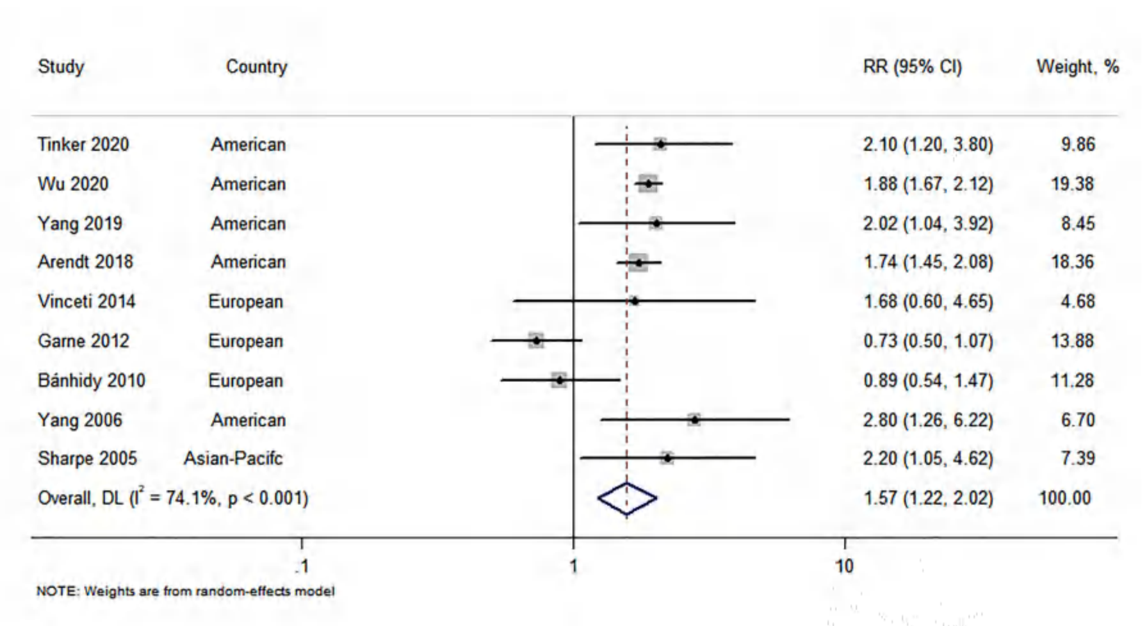

Fig H19. Forest plot of the relative risks of population-based studies on maternal pre-gestational diabetes and the risk of hypospadias (RR = 1.57, 95% CI, 1.22 to 2.02; I^2 = 74.1%, P < 0.001). DL, DerSimonian and Laird random-effects model; RR, relative risk.

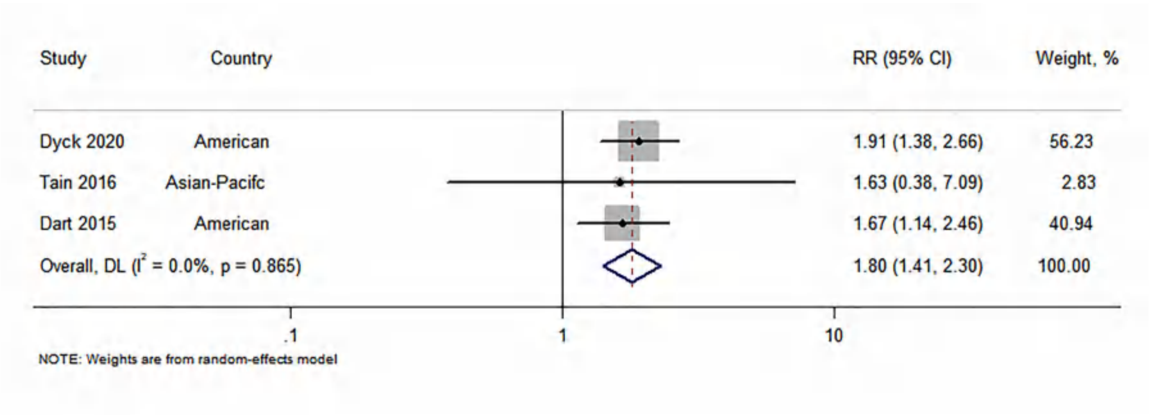

Fig H20. Forest plot of the relative risks of population-based studies on maternal pre-gestational diabetes and the risk of congenital anomalies of the kidney and urinary tract (RR = 1.80, 95% CI, 1.41 to 2.30; I^2 = 0.0%, P = 0.865). DL, DerSimonian and Laird random-effects model; RR, relative risk.

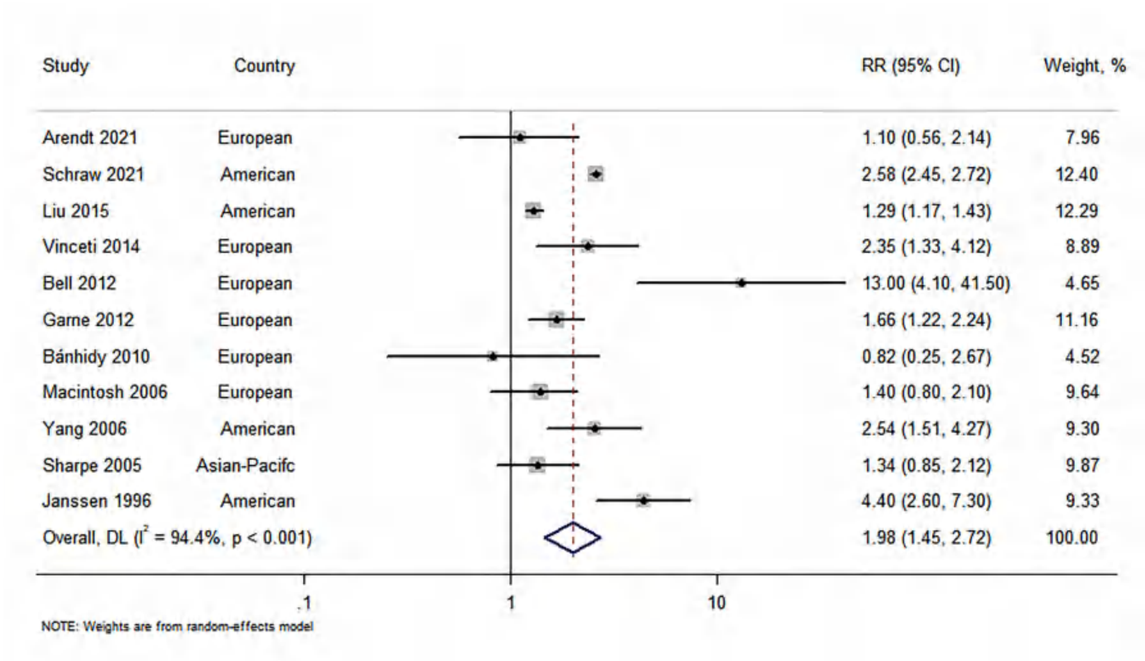

Fig H21. Forest plot of the relative risks of population-based studies on maternal pre-gestational diabetes and the risk of congenital anomalies of musculoskeletal system (RR = 1.98, 95% CI, 1.45 to 2.72; I^2 = 94.4%, P < 0.001). DL, DerSimonian and Laird random-effects model; RR, relative risk.

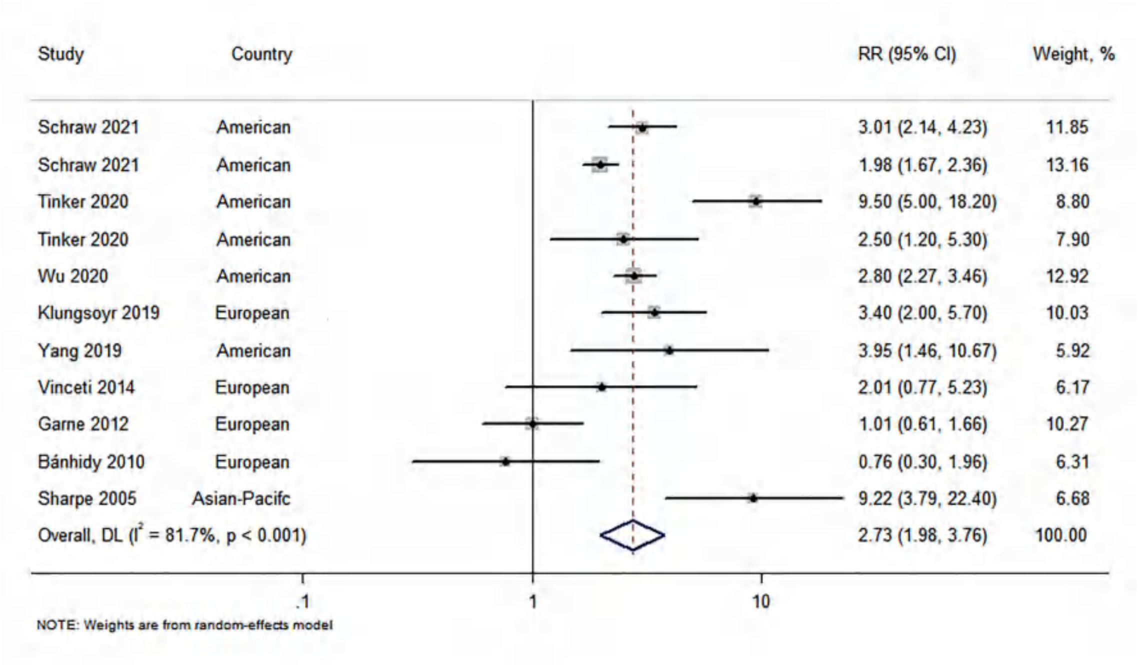

Fig H22. Forest plot of the relative risks of population-based studies on maternal pre-gestational diabetes and the risk of limb reduction (RR = 2.73, 95% CI, 1.98 to 3.76; I^2 = 81.7%, P < 0.001). DL, DerSimonian and Laird random-effects model; RR, relative risk.

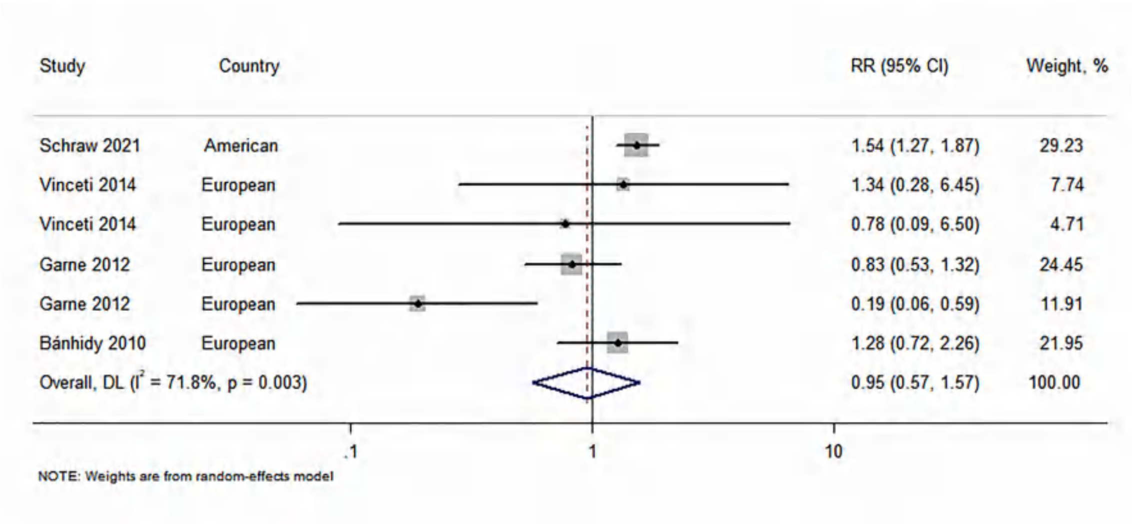

Fig H23. Forest plot of the relative risks of population-based studies on maternal pre-gestational diabetes and the risk of poly/syndactyly (RR = 0.95, 95% CI, 0.57 to 1.57; I^2 = 71.8%, P = 0.003). DL, DerSimonian and Laird random-effects model; RR, relative risk.

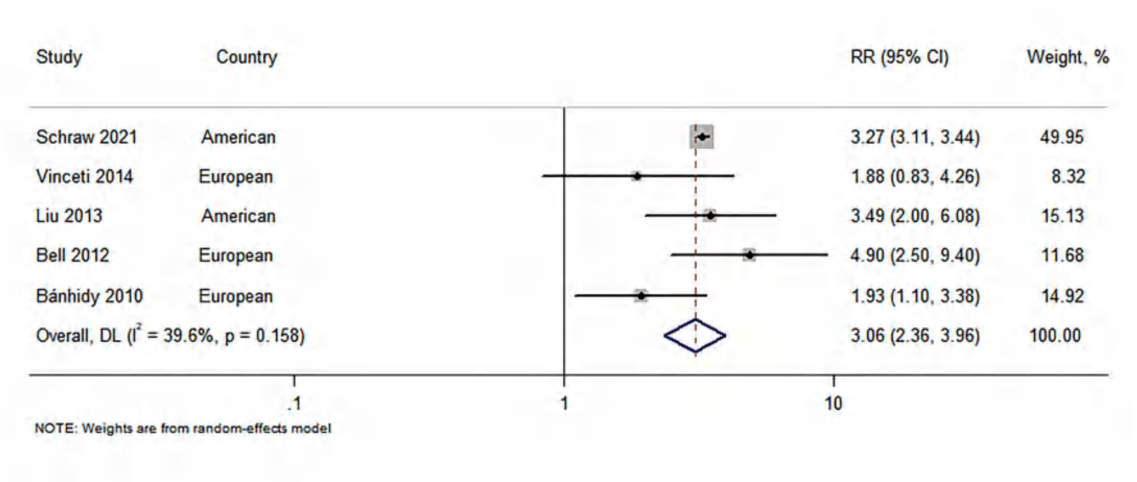

Fig H24. Forest plot of the relative risks of population-based studies on maternal pre-gestational diabetes and the risk of multiple congenital anomalies (RR = 3.06, 95% CI, 2.36 to 3.96; I^2 = 39.6%, P = 0.158). DL, DerSimonian and Laird random-effects model; RR, relative risk.

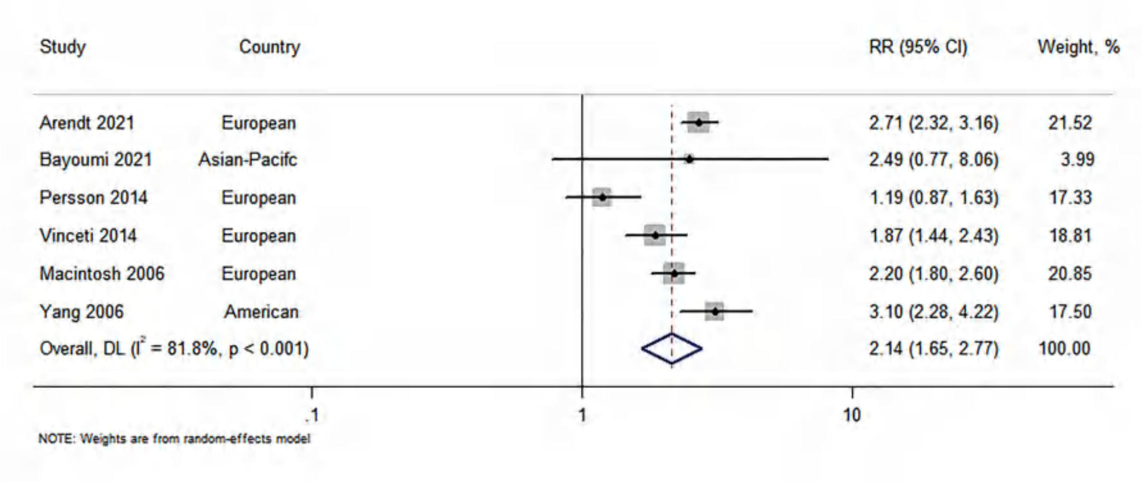

Fig H25. Forest plot of the relative risks of population-based studies on maternal pre-gestational diabetes and the risk of major congenital anomalies (RR = 2.14, 95% CI, 1.65 to 2.77; I^2 = 81.8%, P < 0.001). DL, DerSimonian and Laird random-effects model; RR, relative risk.

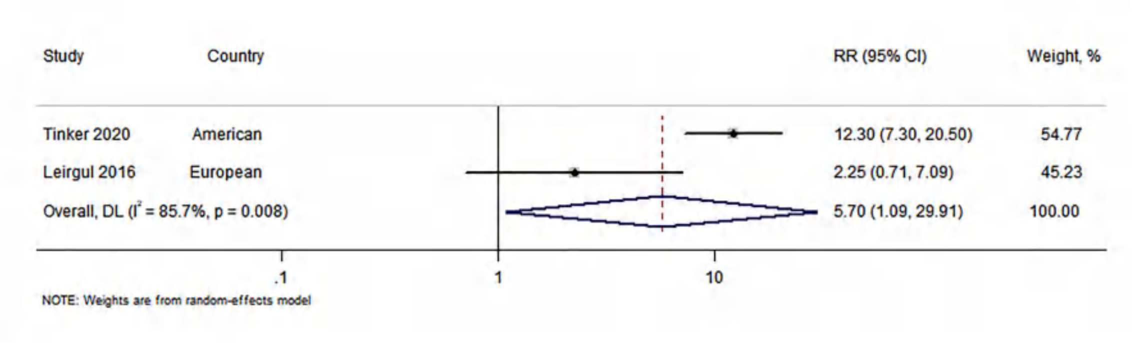

Fig I1. Forest plot of the relative risks of population-based studies on maternal gestational diabetes and the risk of heterotaxia (RR = 5.70, 95% CI, 1.09 to 29.91; I^2 = 85.7%, P = 0.008). DL, DerSimonian and Laird random-effects model; RR, relative risk.

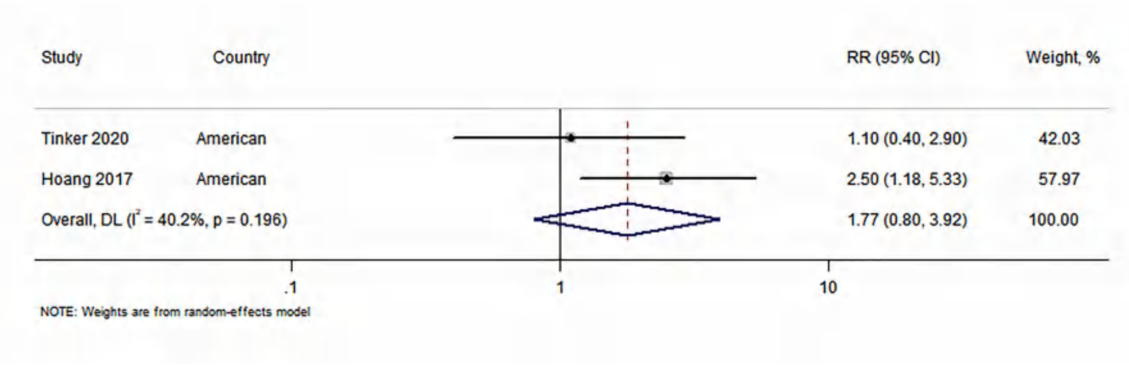

Fig I2. Forest plot of the relative risks of population-based studies on maternal gestational diabetes and the risk of truncus arteriosus (RR = 1.77, 95% CI, 0.80 to 3.92; I^2 = 40.2%, P = 0.196). DL, DerSimonian and Laird random-effects model; RR, relative risk.

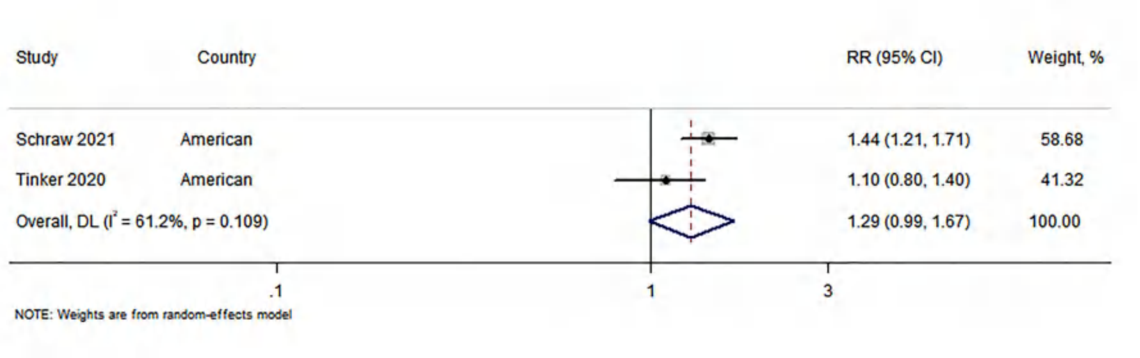

Fig I3. Forest plot of the relative risks of population-based studies on maternal gestational diabetes and the risk of transposition of great vessels (RR = 1.29, 95% CI, 0.99 to 1.67; I^2 = 61.2%, P = 0.109). DL, DerSimonian and Laird random-effects model; RR, relative risk.

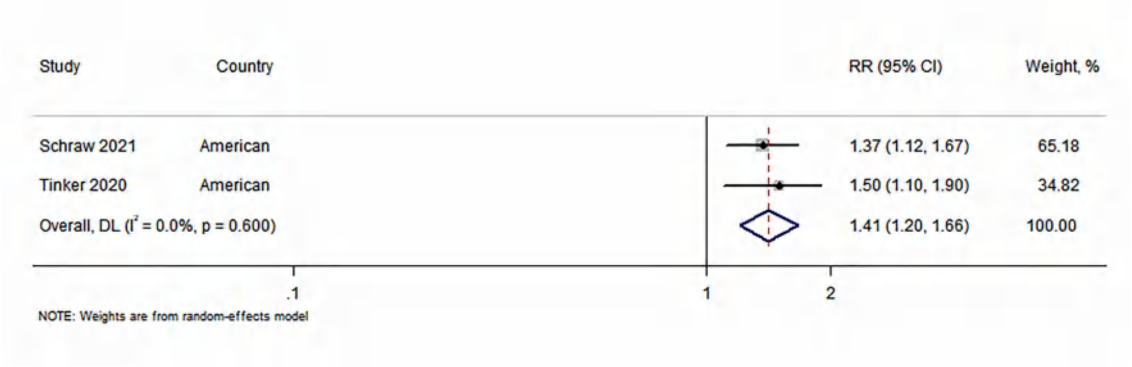

Fig I4. Forest plot of the relative risks of population-based studies on maternal gestational diabetes and the risk of tetralogy of Fallot (RR = 1.41, 95% CI, 1.20 to 1.66; I^2 = 0.0%, P = 0.600). DL, DerSimonian and Laird random-effects model; RR, relative risk.

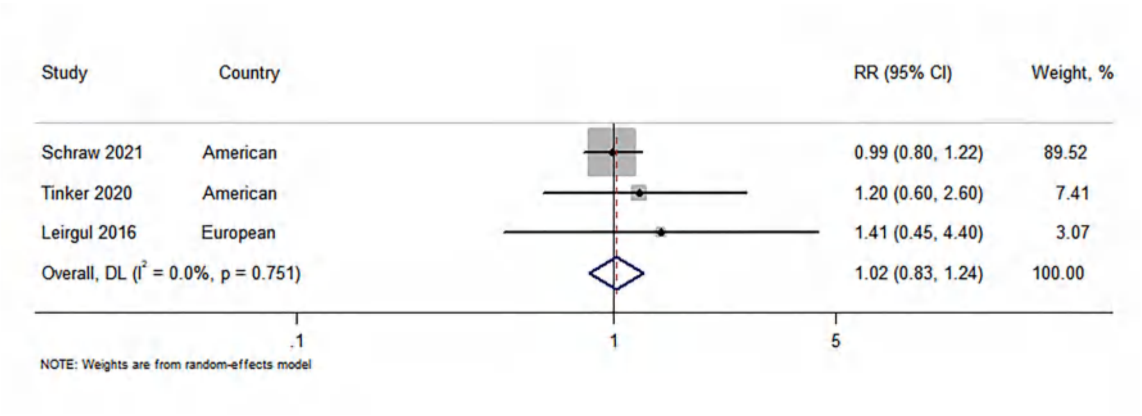

Fig I5. Forest plot of the relative risks of population-based studies on maternal gestational diabetes and the risk of atrioventricular septal defects (RR = 1.02, 95% CI, 0.83 to 1.24; I^2 = 0.0%, P = 0.751). DL, DerSimonian and Laird random-effects model; RR, relative risk.

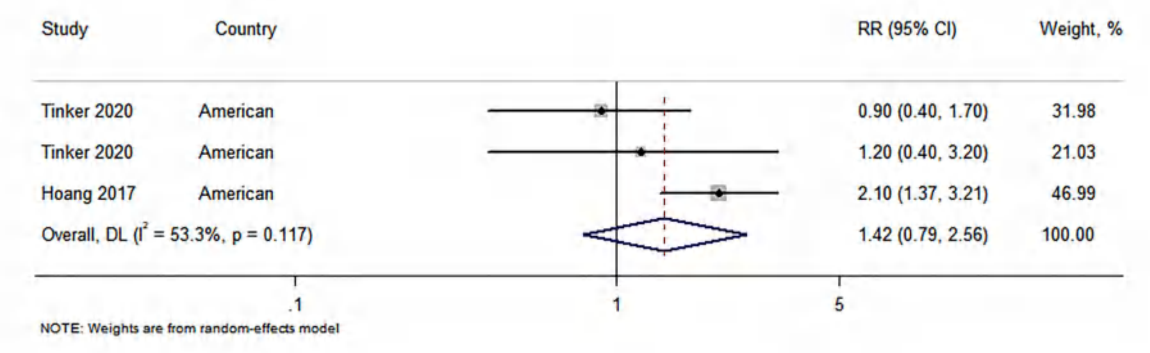

Fig I6. Forest plot of the relative risks of population-based studies on maternal gestational diabetes and the risk of anomalous pulmonary venous return (RR = 1.42, 95% CI, 0.79 to 2.56; I^2 = 53.3%, P = 0.117). DL, DerSimonian and Laird random-effects model; RR, relative risk.

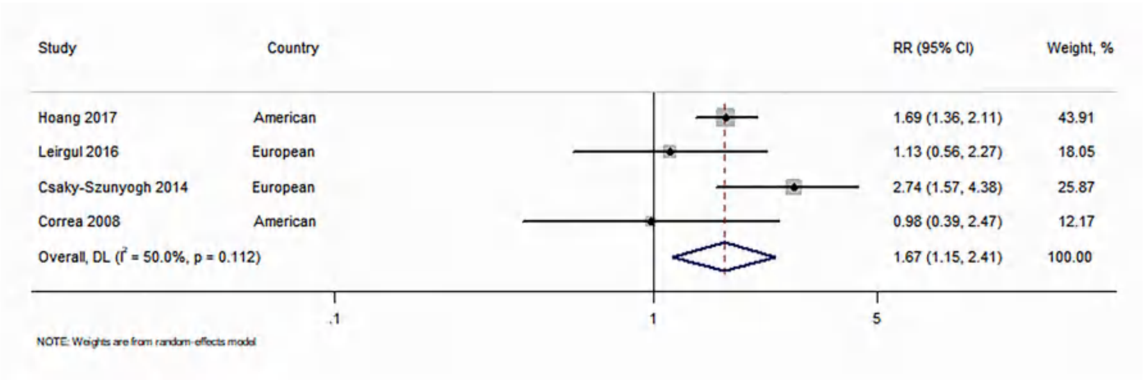

Fig I7. Forest plot of the relative risks of population-based studies on maternal gestational diabetes and the risk of left ventricular outflow tract (RR = 1.67, 95% CI, 1.15 to 2.41; I^2 = 50.0%, P = 0.112). DL, DerSimonian and Laird random-effects model; RR, relative risk.

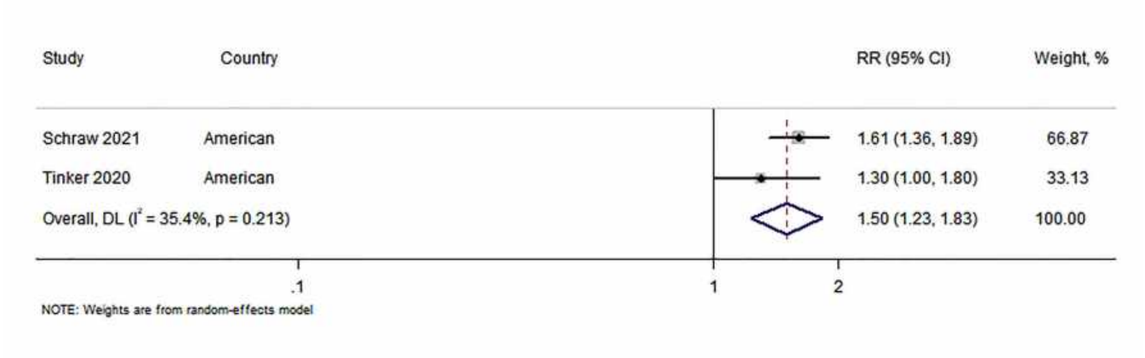

Fig I8. Forest plot of the relative risks of population-based studies on maternal gestational diabetes and the risk of coarctation of aorta (RR = 1.50, 95% CI, 1.23 to 1.83; I^2 = 35.4%, P = 0.213). DL, DerSimonian and Laird random-effects model; RR, relative risk.

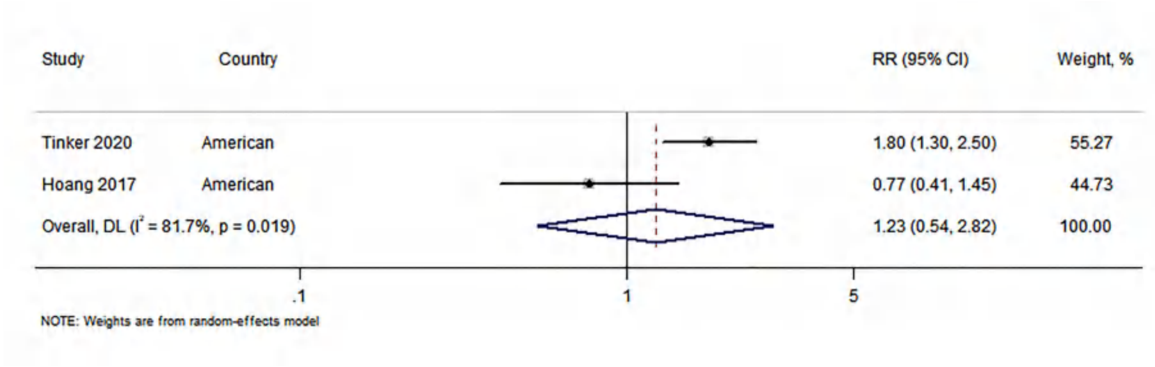

Fig I9. Forest plot of the relative risks of population-based studies on maternal gestational diabetes and the risk of hypoplastic left heart (RR = 1.23, 95% CI, 0.54 to 2.82; I^2 = 81.7%, P = 0.019). DL, DerSimonian and Laird random-effects model; RR, relative risk.

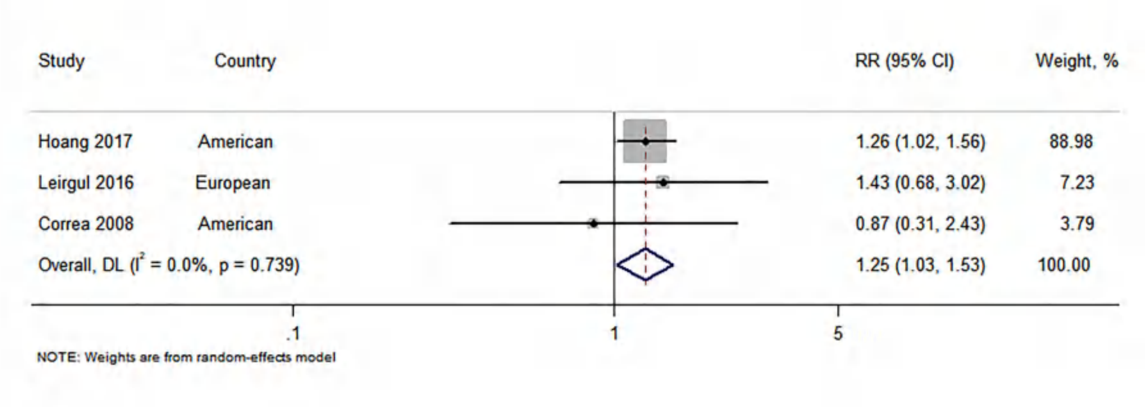

Fig I10. Forest plot of the relative risks of population-based studies on maternal gestational diabetes and the risk of right ventricular outflow tract (RR = 1.25, 95% CI, 1.03 to 1.53; I^2 = 0.0%, P = 0.739). DL, DerSimonian and Laird random-effects model; RR, relative risk.

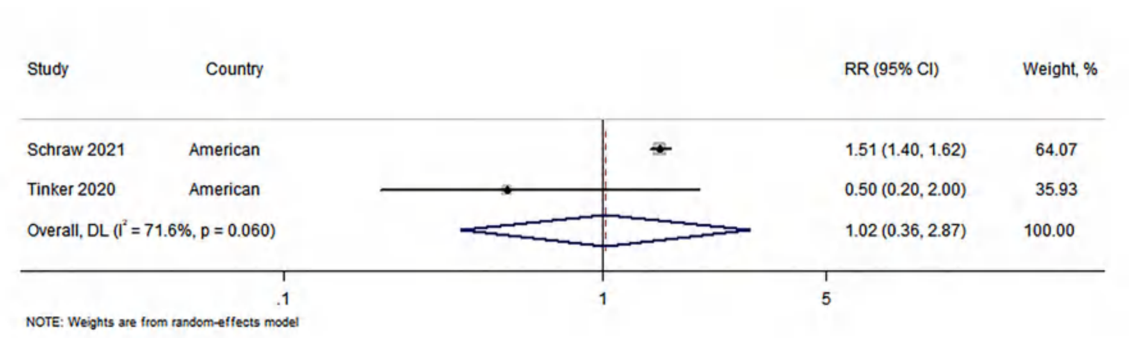

Fig I11. Forest plot of the relative risks of population-based studies on maternal gestational diabetes and the risk of pulmonary artery anomalies (RR = 1.02, 95% CI, 0.36 to 2.87; I^2 = 71.6%, P = 0.060). DL, DerSimonian and Laird random-effects model; RR, relative risk.

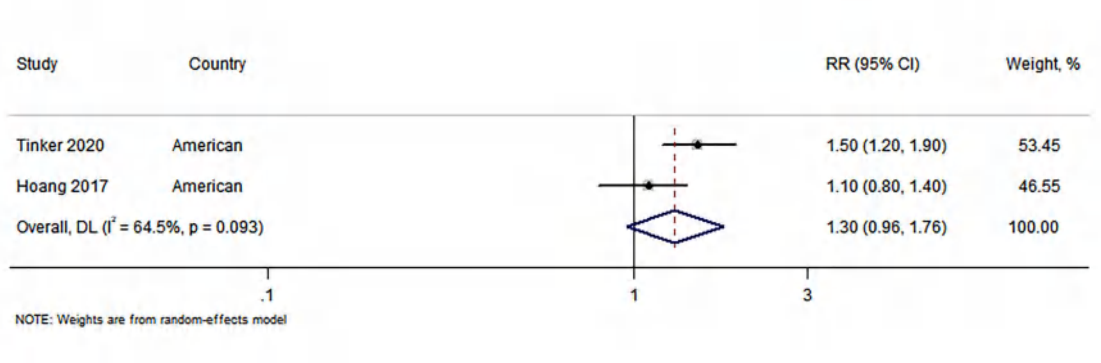

Fig I12. Forest plot of the relative risks of population-based studies on maternal gestational diabetes and the risk of pulmonary valve stenosis (RR = 1.30, 95% CI, 0.96 to 1.76; I^2 = 64.5%, P = 0.093). DL, DerSimonian and Laird random-effects model; RR, relative risk.

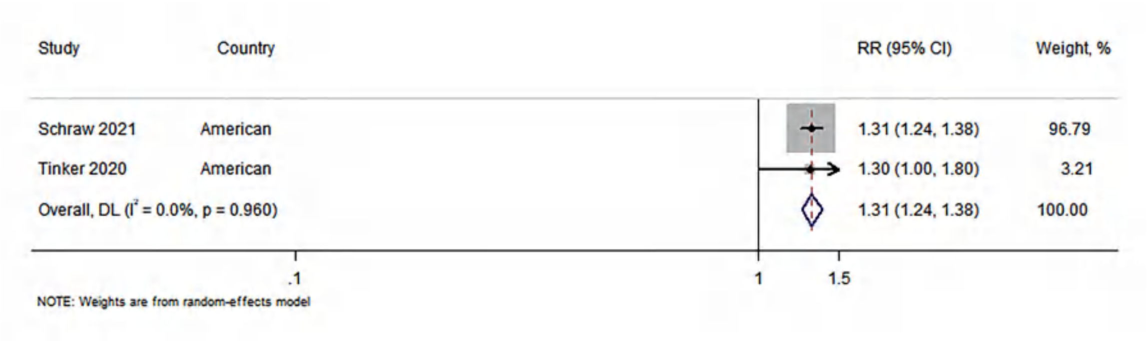

Fig I13. Forest plot of the relative risks of population-based studies on maternal gestational diabetes and the risk of ventricular septal defects (RR = 1.31, 95% CI, 1.24 to 1.38; I^2 = 0.0%, P = 0.960). DL, DerSimonian and Laird random-effects model; RR, relative risk.

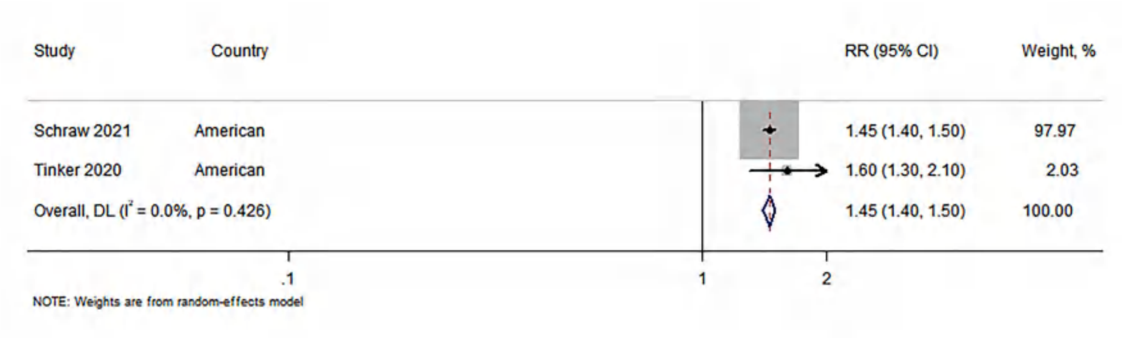

Fig I14. Forest plot of the relative risks of population-based studies on maternal gestational diabetes and the risk of atrial septal defects (RR = 1.45, 95% CI, 1.40 to 1.50; I^2 = 0.0%, P = 0.426). DL, DerSimonian and Laird random-effects model; RR, relative risk.

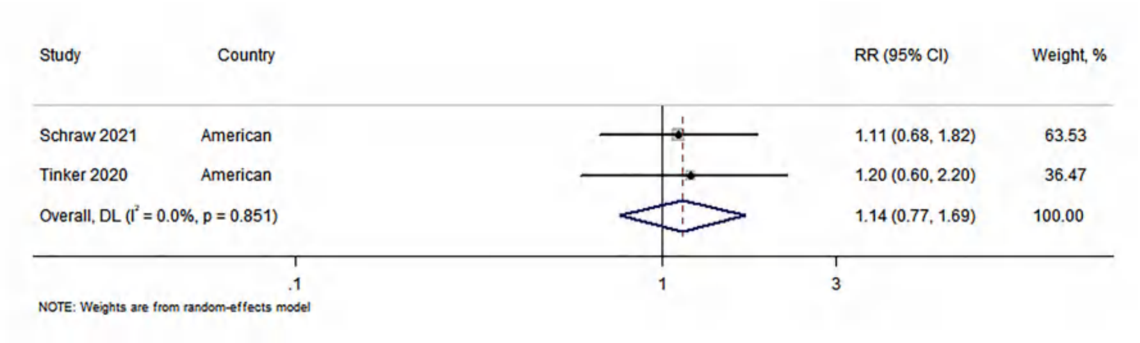

Fig I15. Forest plot of the relative risks of population-based studies on maternal gestational diabetes and the risk of single ventricle (RR = 1.14, 95% CI, 0.77 to 1.69; I^2 = 0.0%, P = 0.851). DL, DerSimonian and Laird random-effects model; RR, relative risk.

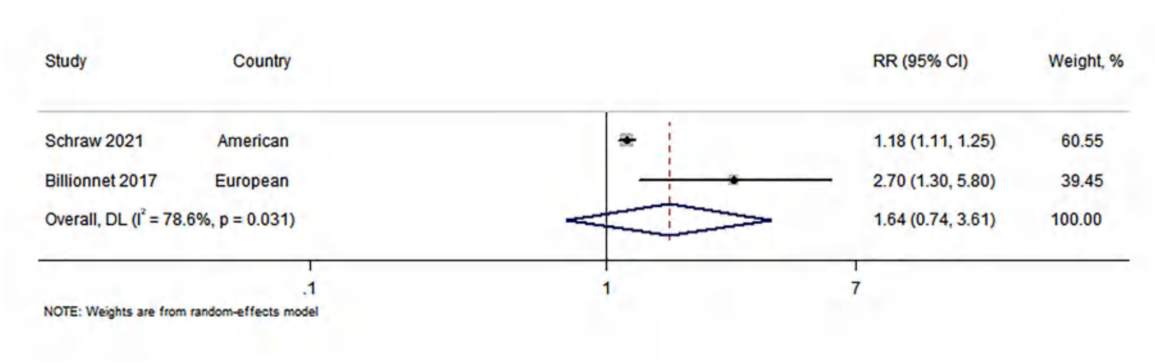

Fig J1. Forest plot of the relative risks of population-based studies on maternal gestational diabetes and the risk of congenital anomalies of nervous system (RR = 1.64, 95% CI, 0.74 to 3.61; I^2 = 78.6%, P = 0.031). DL, DerSimonian and Laird random-effects model; RR, relative risk.

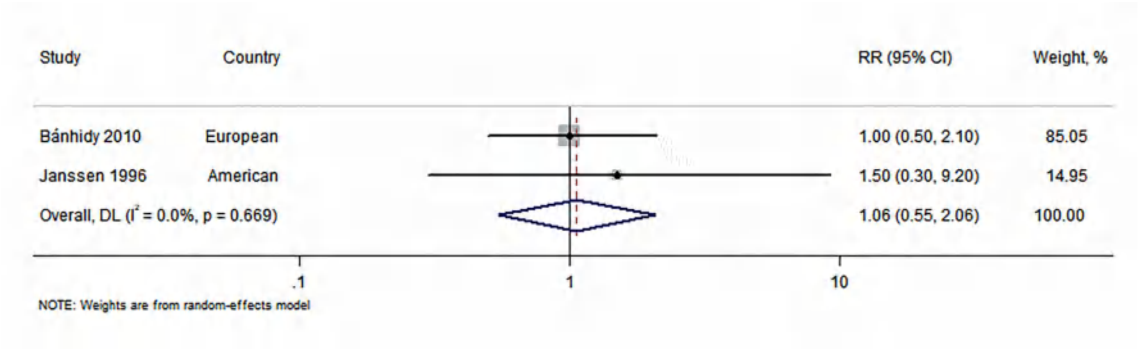

Fig J2. Forest plot of the relative risks of population-based studies on maternal gestational diabetes and the risk of neural tube defects (RR = 1.06, 95% CI, 0.55 to 2.06; I^2 = 0.0%, P = 0.669). DL, DerSimonian and Laird random-effects model; RR, relative risk.

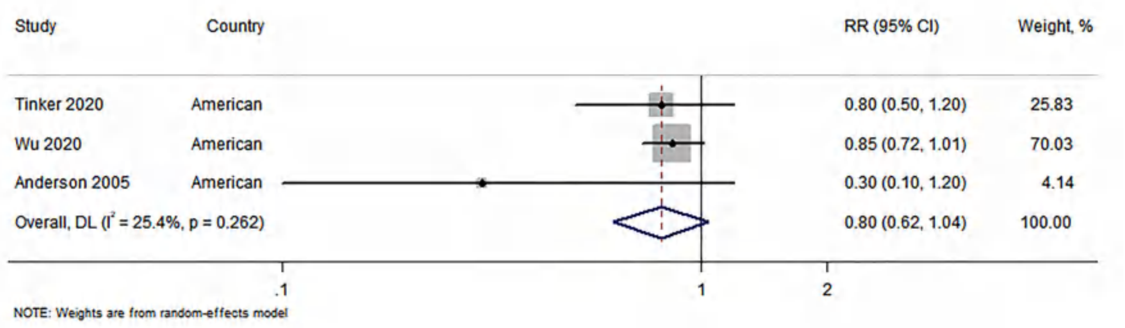

Fig J3. Forest plot of the relative risks of population-based studies on maternal gestational diabetes and the risk of anencephaly (RR = 0.80, 95% CI, 0.62 to 1.04; I^2 = 25.4%, P = 0.262). DL, DerSimonian and Laird random-effects model; RR, relative risk.

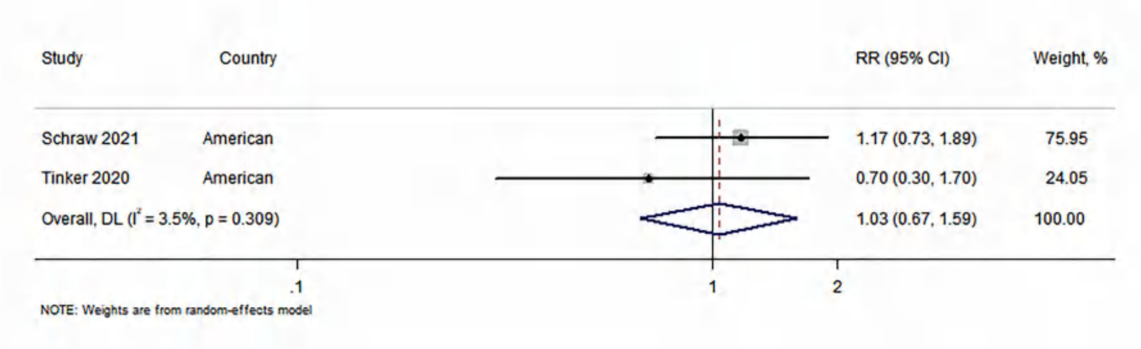

Fig J4. Forest plot of the relative risks of population-based studies on maternal gestational diabetes and the risk of encephalocele (RR = 1.03, 95% CI, 0.67 to 1.59; I^2 = 3.5%, P = 0.309). DL, DerSimonian and Laird random-effects model; RR, relative risk.

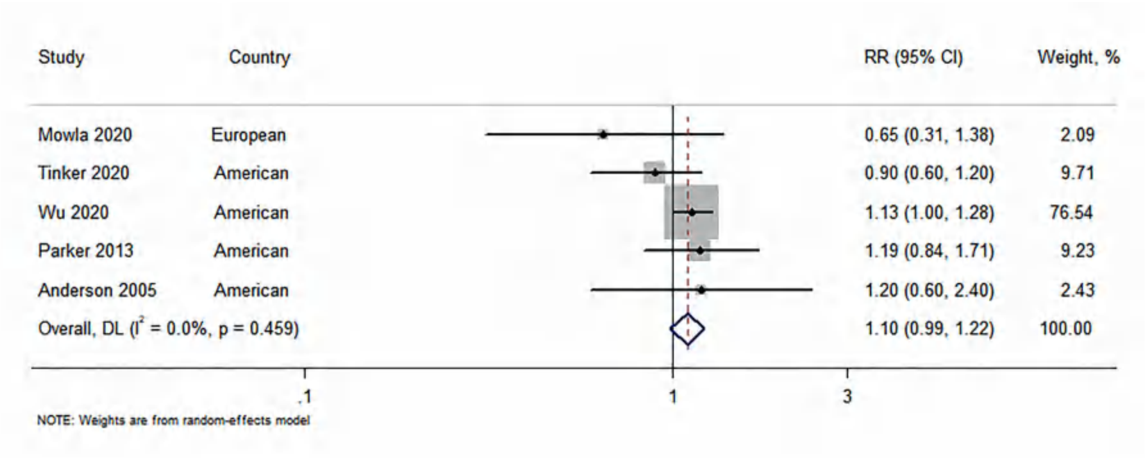

Fig J5. Forest plot of the relative risks of population-based studies on maternal gestational diabetes and the risk of spina bifida (RR = 1.10, 95% CI, 0.99 to 1.22; I^2 = 0.0%, P = 0.459). DL, DerSimonian and Laird random-effects model; RR, relative risk.

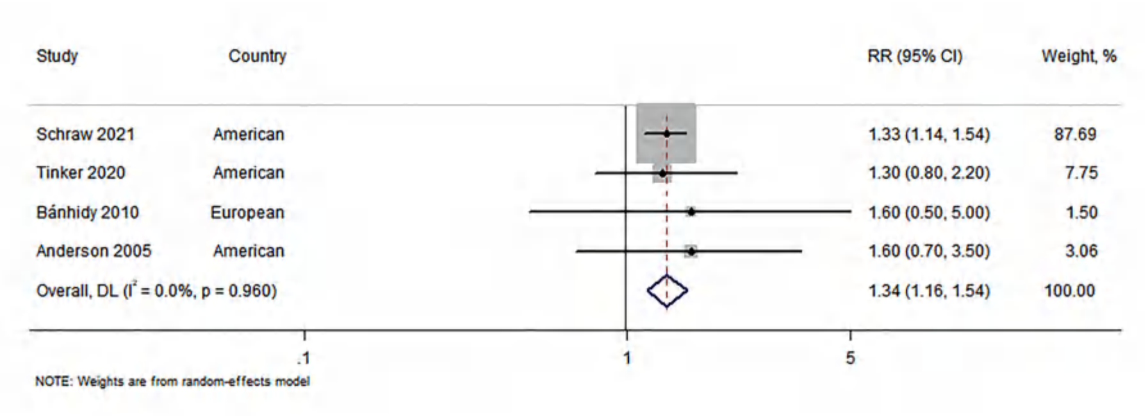

Fig J6. Forest plot of the relative risks of population-based studies on maternal gestational diabetes and the risk of hydrocephaly (RR = 1.34, 95% CI, 1.16 to 1.54; I^2 = 0.0%, P = 0.960). DL, DerSimonian and Laird random-effects model; RR, relative risk.

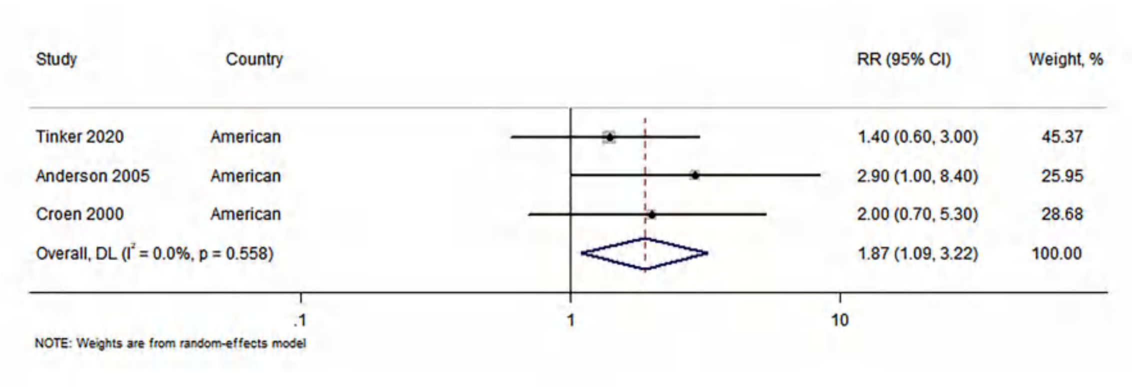

Fig J7. Forest plot of the relative risks of population-based studies on maternal gestational diabetes and the risk of holoprosencephaly (RR = 1.87, 95% CI, 1.09 to 3.22; I^2 = 0.0%, P = 0.558). DL, DerSimonian and Laird random-effects model; RR, relative risk.

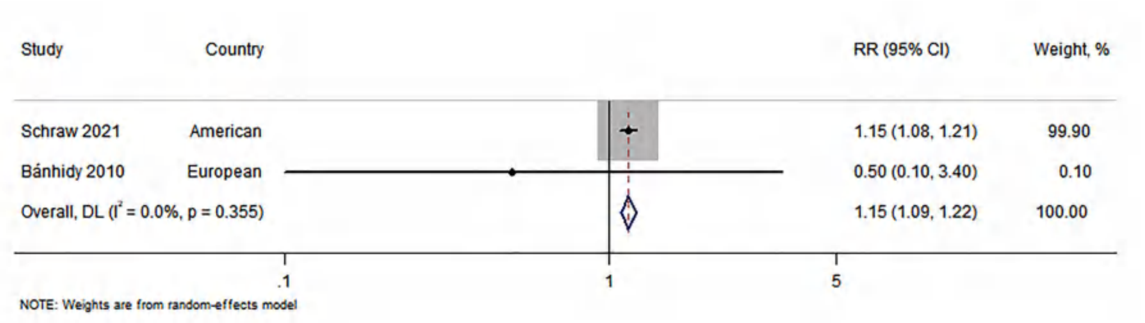

Fig J8. Forest plot of the relative risks of population-based studies on maternal gestational diabetes and the risk of eye, ear, face, and neck (RR = 1.15, 95% CI, 1.09 to 1.22; I^2 = 0.0%, P = 0.355). DL, DerSimonian and Laird random-effects model; RR, relative risk.

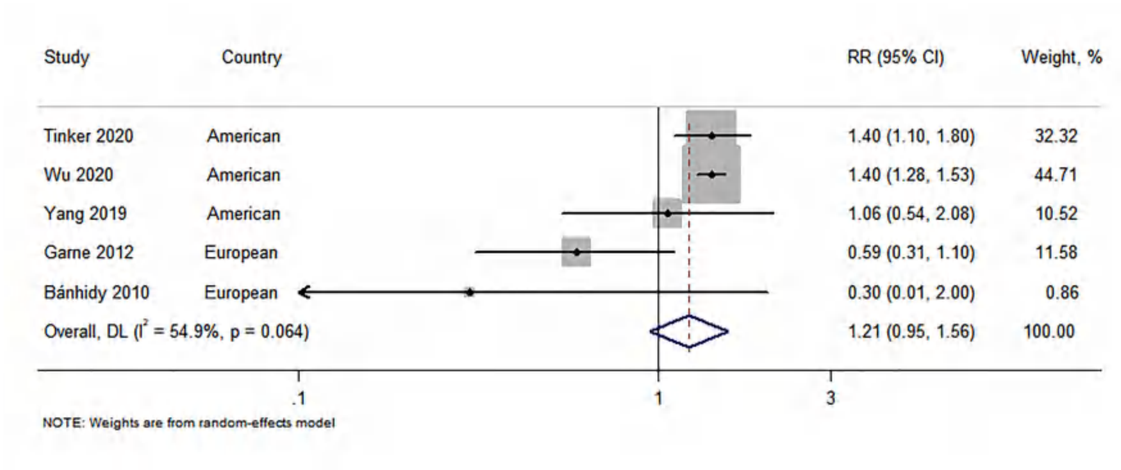

Fig J9. Forest plot of the relative risks of population-based studies on maternal gestational diabetes and the risk of cleft palate (RR = 1.21, 95% CI, 0.95 to 1.56; I^2 = 54.9%, P=0.064). DL, DerSimonian and Laird random-effects model; RR, relative risk.

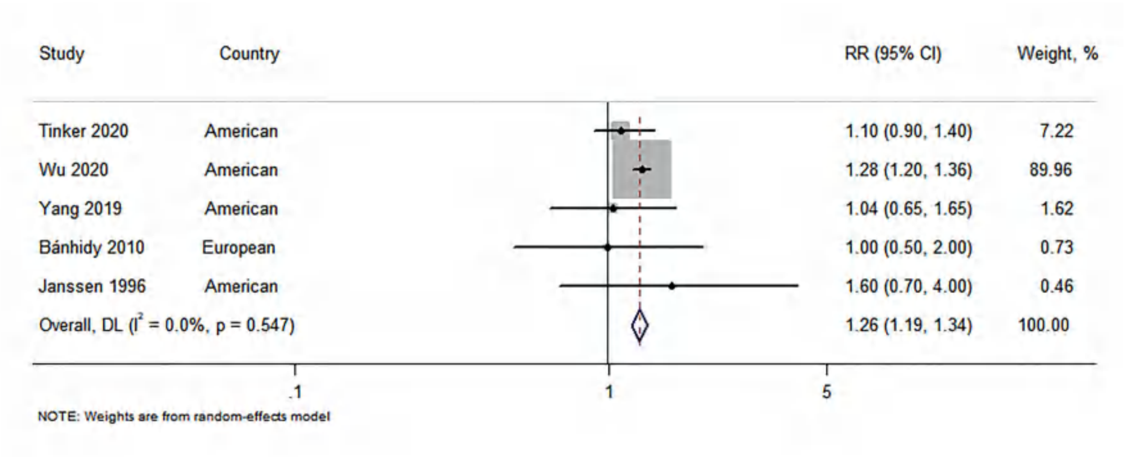

Fig J10. Forest plot of the relative risks of population-based studies on maternal gestational diabetes and the risk of cleft lip with or without palate (RR = 1.26, 95% CI, 1.19 to 1.34; I^2 = 0.0%, P = 0.547). DL, DerSimonian and Laird random-effects model; RR, relative risk.

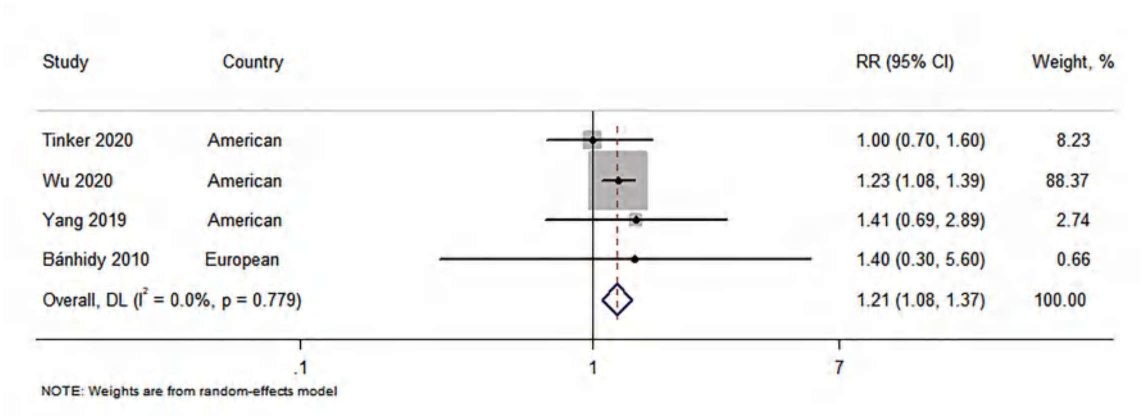

Fig J11. Forest plot of the relative risks of population-based studies on maternal gestational diabetes and the risk of diaphragmatic hernia (RR = 1.21, 95% CI, 1.08 to 1.37; I^2 = 0.0%, P = 0.779). DL, DerSimonian and Laird random-effects model; RR, relative risk.

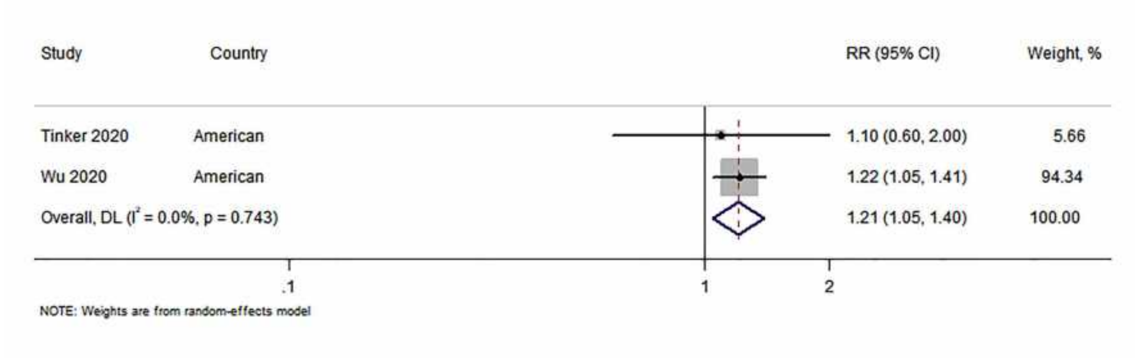

Fig J12. Forest plot of the relative risks of population-based studies on maternal gestational diabetes and the risk of omphalocele (RR = 1.21, 95% CI, 1.05 to 1.40; I^2 = 0.0%, P = 0.743). DL, DerSimonian and Laird random-effects model; RR, relative risk.

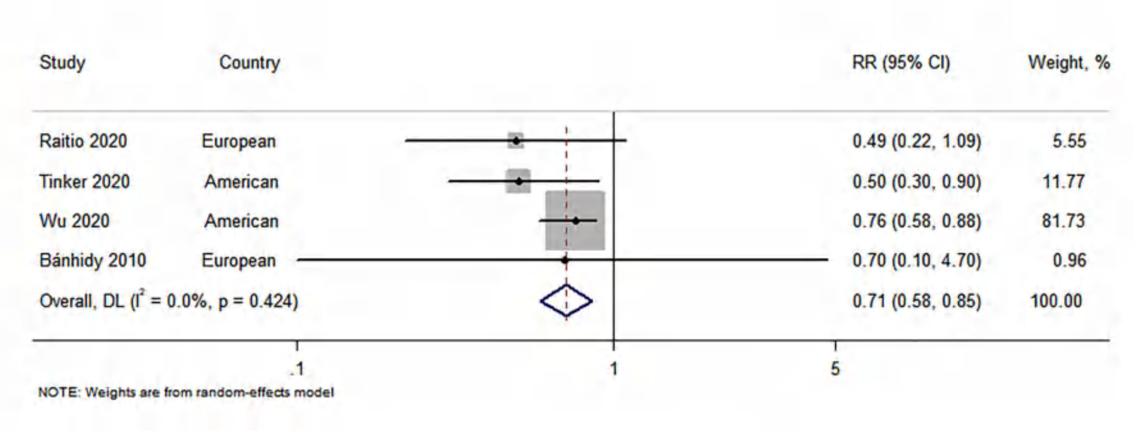

Fig J13. Forest plot of the relative risks of population-based studies on maternal gestational diabetes and the risk of gastroschisis (RR = 0.71, 95% CI, 0.58 to 0.85; I^2 = 0.0%, P = 0.424). DL, DerSimonian and Laird random-effects model; RR, relative risk.

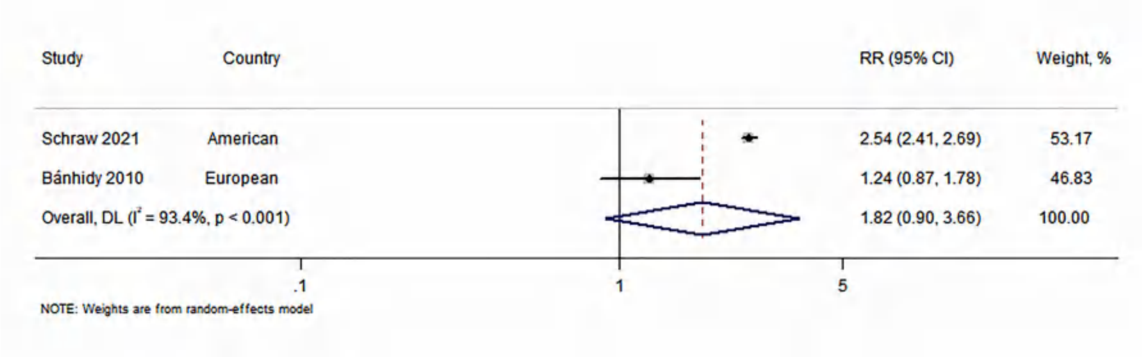

Fig J14. Forest plot of the relative risks of population-based studies on maternal gestational diabetes and the risk of congenital anomalies of genitourinary system (RR = 1.82, 95% CI, 0.90 to 3.66; I^2 = 93.4%, P < 0.001). DL, DerSimonian and Laird random-effects model; RR, relative risk.

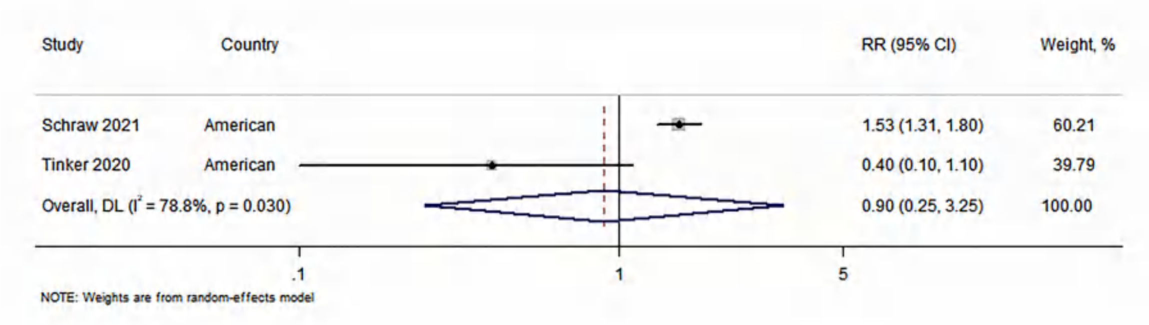

Fig J15. Forest plot of the relative risks of population-based studies on maternal gestational diabetes and the risk of renal agenesis/dysgenesis (RR = 0.90, 95% CI, 0.25 to 3.25; I^2 = 78.8%, P = 0.030). DL, DerSimonian and Laird random-effects model; RR, relative risk.

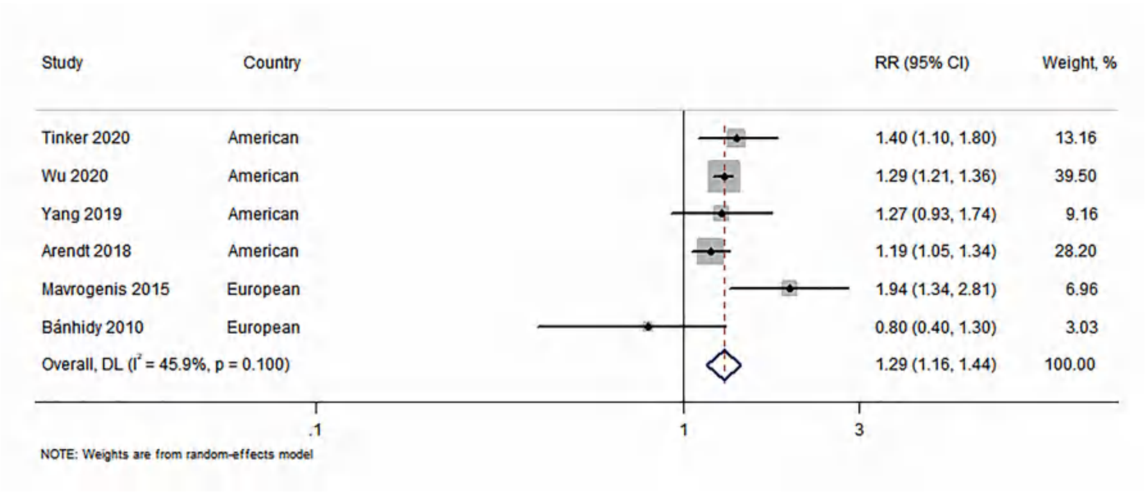

Fig J16. Forest plot of the relative risks of population-based studies on maternal gestational diabetes and the risk of hypospadias (RR = 1.29, 95% CI, 1.16 to 1.44; I^2 = 45.9%, P=0.100). DL, DerSimonian and Laird random-effects model; RR, relative risk.

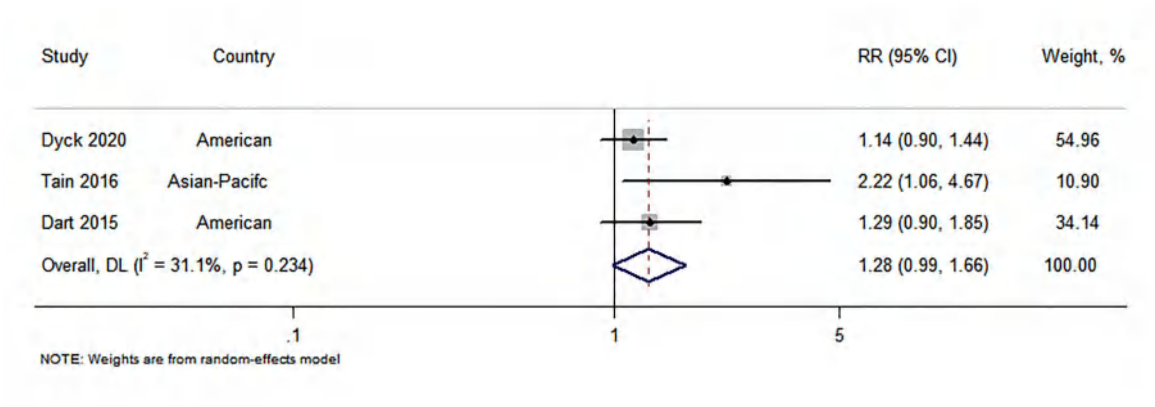

Fig J17. Forest plot of the relative risks of population-based studies on maternal gestational diabetes and the risk of congenital anomalies of the kidney and urinary tract (RR = 1.28, 95% CI, 0.99 to 1.66; I^2 = 31.1%, P = 0.234). DL, DerSimonian and Laird random-effects model; RR, relative risk.

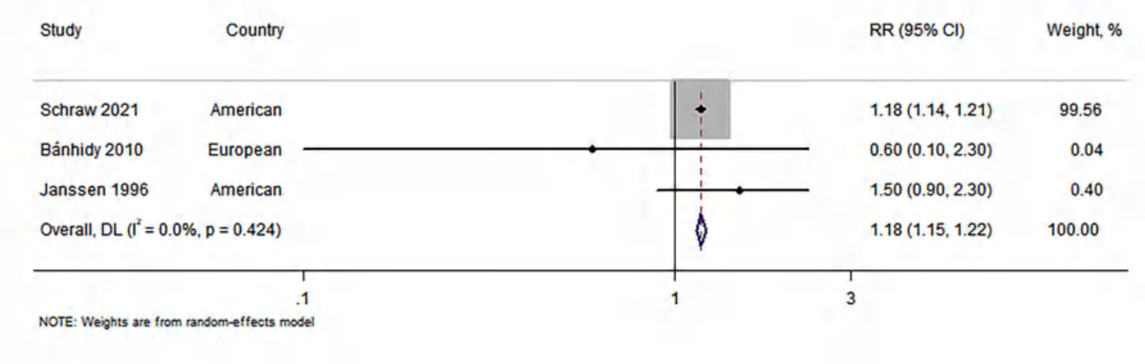

Fig J18. Forest plot of the relative risks of population-based studies on maternal gestational diabetes and the risk of congenital anomalies of musculoskeletal system (RR = 1.18, 95% CI, 1.15 to 1.22; I^2 = 0.0%, P = 0.424). DL, DerSimonian and Laird random-effects model; RR, relative risk.

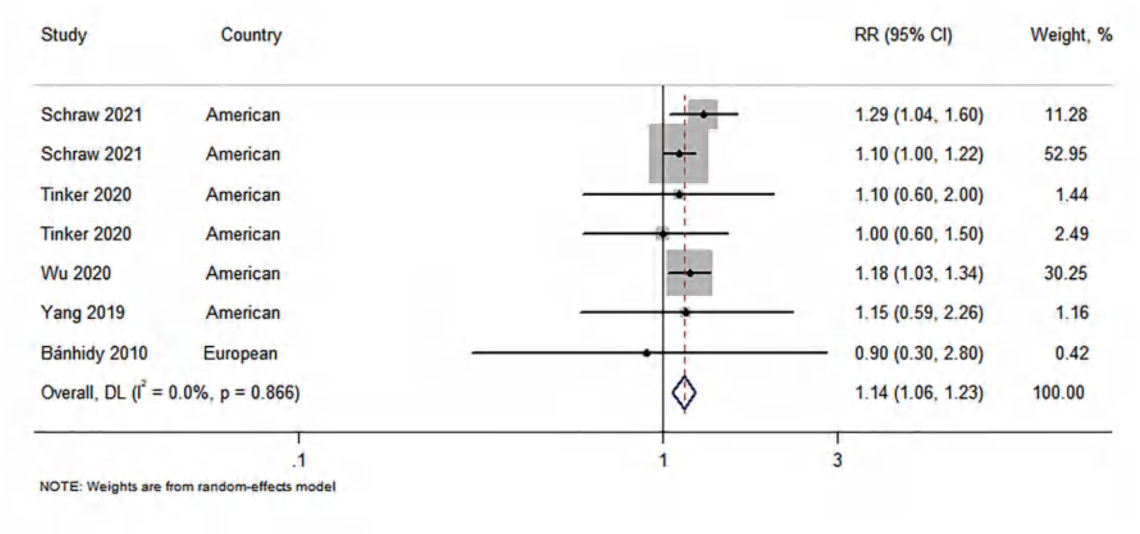

Fig J19. Forest plot of the relative risks of population-based studies on maternal gestational diabetes and the risk of limb reduction (RR = 1.14, 95% CI, 1.06 to 1.23; I^2 = 0.0%, P = 0.866). DL, DerSimonian and Laird random-effects model; RR, relative risk.

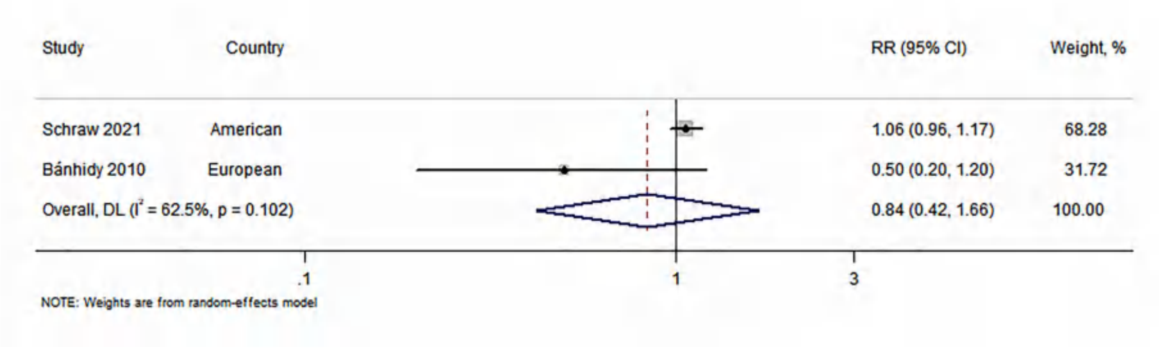

Fig J20. Forest plot of the relative risks of population-based studies on maternal gestational diabetes and the risk of poly/syndactyly (RR = 0.84, 95% CI, 0.42 to 1.66; I^2 = 62.5%, P = 0.102). DL, DerSimonian and Laird random-effects model; RR, relative risk.

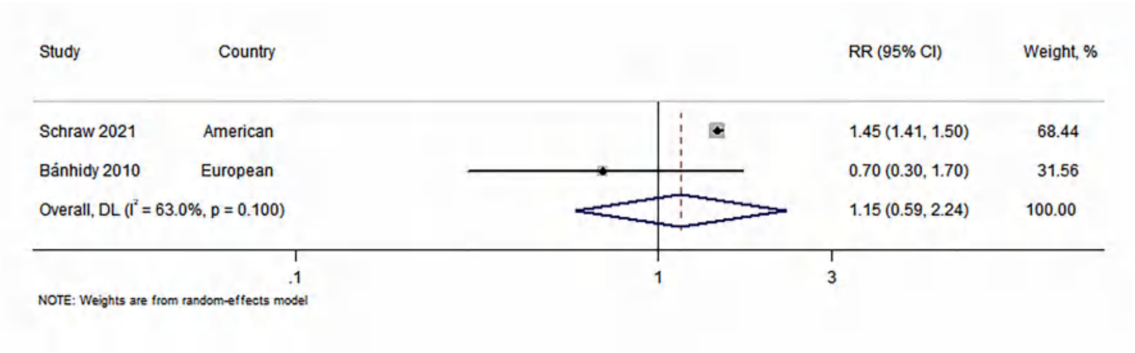

Fig J21. Forest plot of the relative risks of population-based studies on maternal gestational diabetes and the risk of multiple congenital anomalies (RR = 1.15, 95% CI, 0.59 to 2.24; I^2 = 63.0%, P = 0.100). DL, DerSimonian and Laird random-effects model; RR, relative risk.

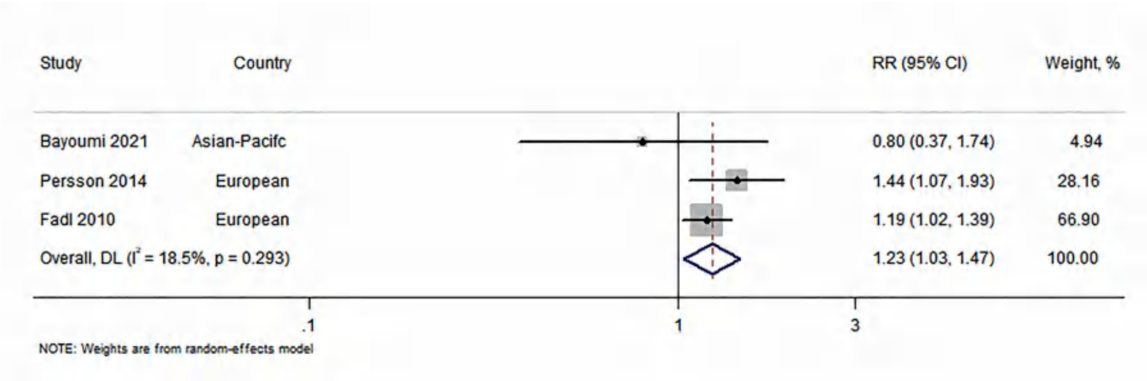

Fig J22. Forest plot of the relative risks of population-based studies on maternal gestational diabetes and the risk of major congenital anomalies (RR = 1.23, 95% CI, 1.03 to 1.47; I^2 = 18.5%, P = 0.293). DL, DerSimonian and Laird random-effects model; RR, relative risk.

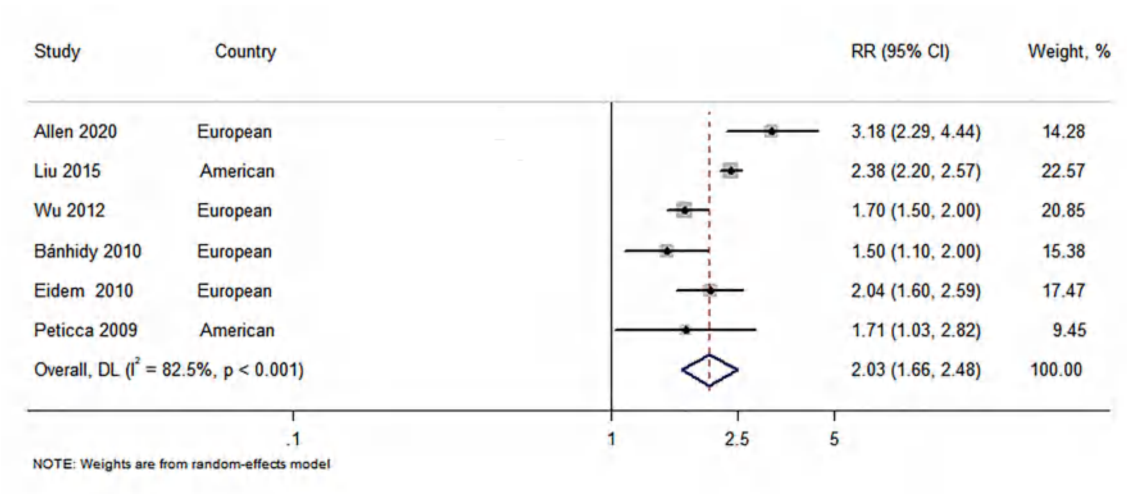

Fig K1. Forest plot of the relative risks of population-based studies on maternal Type 1 diabetes and the risk of overall congenital anomalies (RR = 2.03, 95% CI, 1.66 to 2.48; I^2 = 82.5%, P < 0.001). DL, DerSimonian and Laird random-effects model; RR, relative risk.

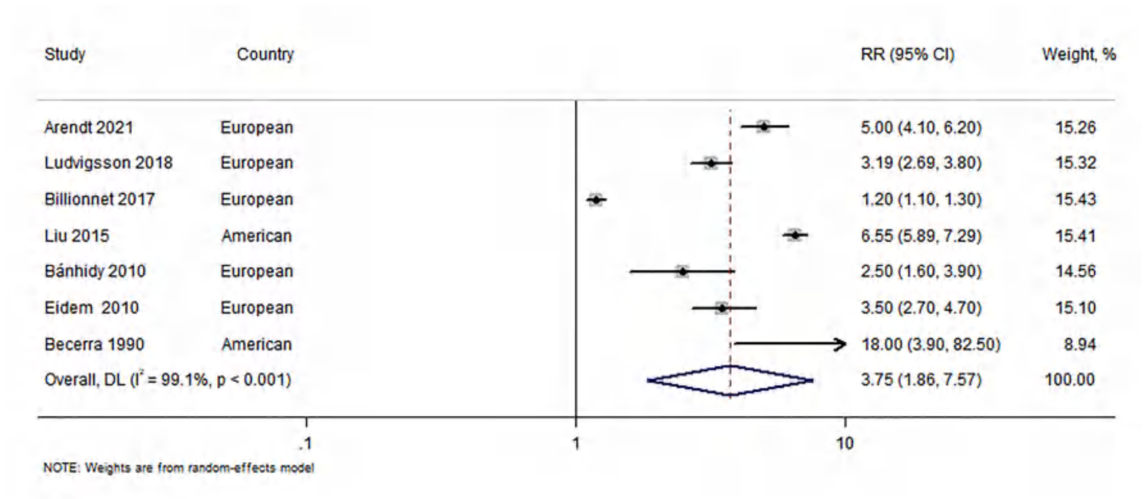

Fig K2. Forest plot of the relative risks of population-based studies on maternal Type 1 diabetes and the risk of congenital heart defects (RR = 3.75, 95% CI, 1.86 to 7.57; I^2 = 99.1%, P < 0.001). DL, DerSimonian and Laird random-effects model; RR, relative risk.

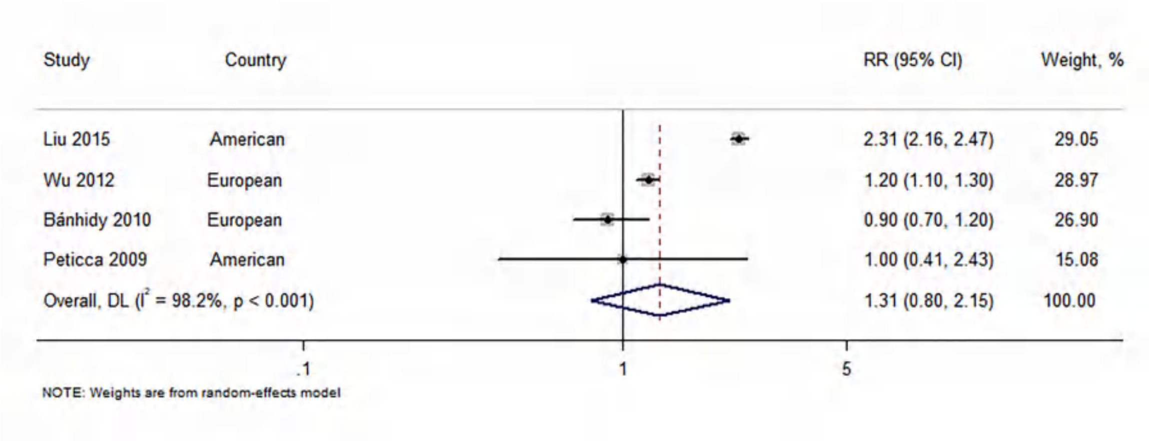

Fig L1. Forest plot of the relative risks of population-based studies on maternal Type 2 diabetes and the risk of overall congenital anomalies (RR = 1.31, 95% CI, 0.80 to 2.15; I^2 = 98.2%, P < 0.001). DL, DerSimonian and Laird random-effects model; RR, relative risk.

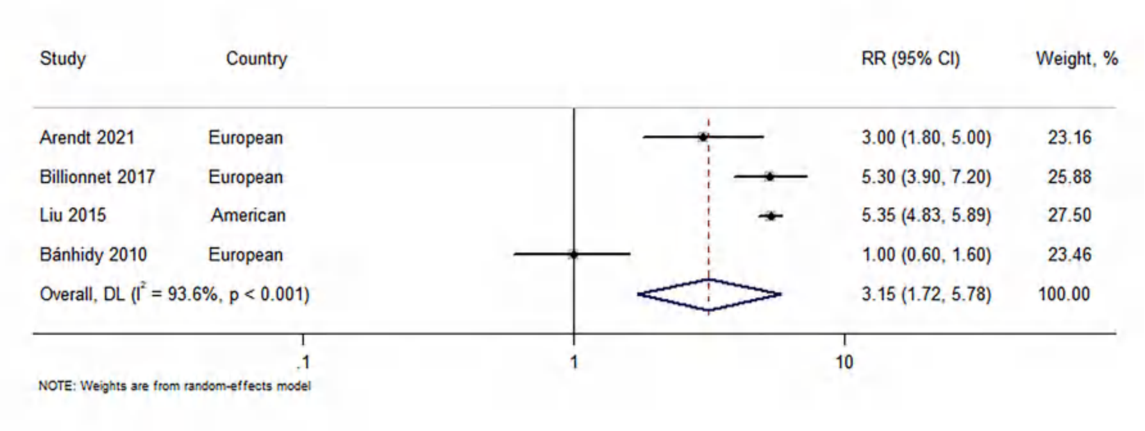

Fig L2. Forest plot of the relative risks of population-based studies on maternal Type 2 diabetes and the risk of congenital heart defects (RR = 3.15, 95% CI, 1.72 to 5.78; I^2 = 93.6%, P < 0.001). DL, DerSimonian and Laird random-effects model; RR, relative risk.

Reference cited in all supplementary figures

1.Arendt LH, Pedersen LH, Pedersen L, Ovesen PG, Henriksen TB, Lindhard MS, et al. Glycemic Control in Pregnancies Complicated by Pre-Existing Diabetes Mellitus and Congenital Malformations: A Danish Population-Based Study. Clin Epidemiol. 2021;13:615–26.

2.Bayoumi MAA, Masri RM, Matani NYS, Hendaus MA, Masri MM, Chandra P, et al. Maternal and neonatal outcomes in mothers with diabetes mellitus in qatari population.BMC Pregnancy Childbirth. 2021;21(1):651

3.Schraw JM, Langlois PH, Lupo PJ. Comprehensive assessment of the associations between maternal diabetes and structural birth defects in offspring: a phenome-wide association study. Ann Epidemiol. 2021;53:14–20.e8.

4.Allen LA, Cannings-John RL, Evans A, Thayer DS, French R, Paranjothy S, et al. Pregnancy in teenagers diagnosed with type 1 diabetes mellitus in childhood: a national population-based e-cohort study. Diabetologia. 2020;63:799–810.

5.Dyck RF, Karunanayake C, Pahwa P, Stang M, Erickson RL, Osgood ND. Congenital Anomalies of the Kidney and Urinary Tract (CAKUT): An Emerging Relationship With Pregestational Diabetes Mellitus Among First Nations and Non-First Nations People in Saskatchewan-Results From the DIP: ORRIIGENSS Project. Can J Diabetes. 2020:S1499–2671.

6.Lee D, Booth GL, Ray JG, Ling V, Feig DS. Undiagnosed type 2 diabetes during pregnancy is associated with increased perinatal mortality: a large population-based cohort study in Ontario, Canada. Diabet Med. 2020;37:1696–1704.

7.Mowla S, Gissler M, Räisänen S, Kancherla V. Association between maternal pregestational diabetes mellitus and spina bifida: A population-based case-control study, Finland, 2000-2014. Birth Defects Res. 2020;112:186–95.

8.Oliveira-Brancati CIF, Ferrarese VCC, Costa AR, Fett-Conte AC. Birth defects in Brazil: Outcomes of a population-based study. Genet Mol Biol. 2020;43:e20180186.

9.Raitio A, Tauriainen A, Leinonen MK, Syvänen J, Kemppainen T, Löyttyniemi E, et al. Maternal risk factors for gastroschisis: A population-based case-control study. Birth Defects Res. 2020;112:989–95.

10.Seghieri G, Di Cianni G, Seghieri M, Lacaria E, Corsi E, Lencioni C, et al. Risk and adverse outcomes of gestational diabetes in migrants: A population cohort study. Diabetes Res Clin Pract. 2020;163:108128.

11.Tinker SC, Gilboa SM, Moore CA, Waller DK, Simeone RM, Kim SY, et al. Specific birth defects in pregnancies of women with diabetes: National Birth Defects Prevention Study, 1997–2011. Am J Obstet Gynecol. 2020;222:176.e1–11.

12.Wu Y, Liu B, Sun Y, Du Y, Santillan MK, Santillan DA, et al. Association of Maternal Prepregnancy Diabetes and Gestational Diabetes Mellitus With Congenital Anomalies of the Newborn. Diabetes Care. 2020;43:2983–90.

13.Chen L, Wang WJ, Auger N, Xiao L, Torrie J, McHugh NG, et al. Diabetes in pregnancy in associations with perinatal and postneonatal mortality in First Nations and non-Indigenous populations in Quebec, Canada: population-based linked birth cohort study. BMJ Open. 2019;9:e025084.

14.Hildén K, Hanson U, Persson M, Magnuson A, Simmons D, Fadl H. Gestational diabetes and adiposity are independent risk factors for perinatal outcomes: a population based cohort study in Sweden. Diabet Med. 2019;36:151–57.

15.Klungsøyr K, Nordtveit TI, Kaastad TS, Solberg S, Sletten IN, Vik AK. Epidemiology of limb reduction defects as registered in the Medical Birth Registry of Norway, 1970-2016: Population based study. PLoS One. 2019;14(7):e0219930.
16.Liu S, Evans J, MacFarlane AJ, Ananth CV, Little J, Kramer MS, et al. Association of maternal risk factors with the recent rise of neural tube defects in Canada. Paediatr Perinat Epidemiol. 2019;33:145–53.
17.Wei Y, Xu Q, Yang H, Yang Y, Wang L, Chen H, et al. Preconception diabetes mellitus and adverse pregnancy outcomes in over 6.4 million women: A population-based cohort study in China. PLoS Med. 2019;16:e1002926.
18.Yang G-R, Dye TD, Li D. Effects of pre-gestational diabetes mellitus and gestational diabetes mellitus on macrosomia and birth defects in Upstate New York. Diabetes Res Clin Pract. 2019;155:107811.
19.Ludvigsson JF, Neovius M, Söderling J, Gudbjörnsdottir S, Svensson AM, Franzén S, et al. Periconception glycaemic control in women with type 1 diabetes and risk of major birth defects: population based cohort study in Sweden. BMJ. 2018;362:k2638.
20.Arendt LH, Lindhard MS, Henriksen TB, Olsen J, Cnattingius S, Petersson G, et al. Maternal Diabetes Mellitus and Genital Anomalies in Male Offspring: A Nationwide Cohort Study in 2 Nordic Countries. Epidemiology. 2018;29:280–89.
21.Kovalenko AA, Anda EE, Odland JØ, Nieboer E, Brenn T, Krettek A. Risk Factors for Ventricular Septal Defects in Murmansk County, Russia: A Registry-Based Study. Int J Env Res Public Health. 2018;15:1320.
22.Soliman A, Salama H, Al Rifai H, De Sanctis V, Al-Obaidly S, Al Qubasi M, et al. The effect of different forms of dysglycemia during pregnancy on maternal and fetal outcomes in treated women and comparison with large cohort studies. Acta Biomed. 2018;89:11–21.
23.Billionnet C, Mitanchez D, Weill A, Nizard J, Alla F, Hartemann A, et al. Gestational diabetes and adverse perinatal outcomes from 716,152 births in France in 2012. Diabetologia. 2017;60:636–44.
24.Hoang TT, Marengo LK, Mitchell LE, Canfield MA, Agopian AJ. Original Findings and Updated Meta-Analysis for the Association Between Maternal Diabetes and Risk for Congenital Heart Disease Phenotypes. Am J Epidemiol. 2017;186:118–28.
25.Darke J, Glinianaia SV, Marsden P, Bell R. Pregestational diabetes is associated with adverse outcomes in twin pregnancies: a regional register-based study. Acta Obstet Gynecol Scand. 2016;95:339–46.
26.Lai FY, Johnson JA, Dover D, Kaul P. Outcomes of singleton and twin pregnancies complicated by pre-existing diabetes and gestational diabetes: A population-based study in Alberta, Canada, 2005–11. J Diabetes. 2016;8:45–55.
27.Leirgul E, Brodwall K, Greve G, Vollset SE, Holmstrøm H, Tell GS, et al. Maternal Diabetes, Birth Weight, and Neonatal Risk of Congenital Heart Defects in Norway, 1994–2009. Obstet Gynecol. 2016;128:1116–25.
28.Øyen N, Diaz LJ, Leirgul E, Boyd HA, Priest J, Mathiesen ER, et al. Prepregnancy Diabetes and Offspring Risk of Congenital Heart Disease: A Nationwide Cohort Study. Circulation. 2016;133:2243–53.
29.Tain Y-L, Luh H, Lin C-Y, Hsu C-N. Incidence and Risks of Congenital Anomalies of Kidney and Urinary Tract in Newborns: A Population-Based Case-Control Study in Taiwan. Medicine. 2016;95:e2659.

30.Dart AB, Ruth CA, Sellers EA, Au W, Dean HJ. Maternal diabetes mellitus and congenital anomalies of the kidney and urinary tract (CAKUT) in the child. Am J Kidney Dis. 2015;65:684–91.
31.Liu S, Rouleau J, León JA, Sauve R, Joseph KS, Ray JG. Impact of pre-pregnancy diabetes mellitus on congenital anomalies, Canada, 2002–2012. Health Promot Chronic Dis Prev Can. 2015;35:79–84.
32.Liu X, Liu G, Wang P, Huang Y, Liu E, Li D, et al. Prevalence of congenital heart disease and its related risk indicators among 90,796 Chinese infants aged less than 6 months in Tianjin. Int J Epidemiol. 2015;44:884–93.
33.Mavrogenis S, Urban R, Czeizel AE. Pregnancy complications in the mothers who delivered boys with isolated hypospadias - a population-based case-control study. J Matern Fetal Neona. 2015;28:489–93.
34.Csáky-Szunyogh M, Vereczkey A, Kósa Z, Gerencsér B, Czeizel AE. Risk factors in the origin of congenital left-ventricular outflow-tract obstruction defects of the heart: a population-based case-control study. Pediatr Cardiol. 2014;35:108–20.
35.Feig DS, Hwee J, Shah BR, Booth GL, Bierman AS, Lipscombe LL. Trends in incidence of diabetes in pregnancy and serious perinatal outcomes: a large, population-based study in Ontario, Canada, 1996–2010. Diabetes Care. 2014;37:1590–96.
36.Persson M, Fadl H. Perinatal outcome in relation to fetal sex in offspring to mothers with pre-gestational and gestational diabetes--a population-based study. Diabet Med. 2014;31:1047–54.
37.Vereczkey A, Gerencsér B, Czeizel AE, Szabó I. Association of certain chronic maternal diseases with the risk of specific congenital heart defects: a population-based study. Eur J Obstet Gynecol Reprod Biol. 2014;182:1–6.
38.Vinceti M, Malagoli C, Rothman KJ, Rodolfi R, Astolfi G, Calzolari E, et al. Risk of birth defects associated with maternal pregestational diabetes. Eur J Epidemiol. 2014; 29:411–18.
39.Liu S, Joseph KS, Lisonkova S, Rouleau J, Van den Hof M, Sauve R, et al. Association between maternal chronic conditions and congenital heart defects: a population-based cohort study. Circulation. 2013;128:583–89.
40.Parker SE, Yazdy MM, Tinker SC, Mitchell AA, Werler MM. The impact of folic acid intake on the association among diabetes mellitus, obesity, and spina bifida. Am J Obstet Gynecol. 2013;209:239.e231–38.
41.Bell R, Glinianaia SV, Tennant PWG, Bilous RW, Rankin J. Peri-conception hyperglycaemia and nephropathy are associated with risk of congenital anomaly in women with pre-existing diabetes: a population-based cohort study. Diabetologia. 2012;55:936–47.
42.Garne E, Loane M, Dolk H, Barisic I, Addor MC, Arriola L, et al. Spectrum of congenital anomalies in pregnancies with pregestational diabetes. Birth Defects Res A Clin Mol Teratol. 2012;94:134–40.
43.Wu CS, Nohr EA, Bech BH, Vestergaard M, Olsen J. Long-term health outcomes in children born to mothers with diabetes: a population-based cohort study. PLoS One. 2012;7:e36727.
44.Alverson CJ, Strickland MJ, Gilboa SM, Correa A. Maternal smoking and congenital heart defects in the Baltimore-Washington Infant Study. Pediatrics. 2011;127:e647–53.
45.Bánhidy F, Acs N, Puhó EH, Czeizel AE. Congenital abnormalities in the offspring of pregnant women with type 1, type 2 and gestational diabetes mellitus: a population-based case-control study. Congenit Anom (Kyoto). 2010;50:115–21.
46.Eidem I, Stene LC, Henriksen T, Hanssen KF, Vangen S, Vollset SE, et al. Congenital anomalies in newborns of women with type 1 diabetes: nationwide population-based study in Norway, 1999-2004. Acta Obstet Gynecol Scand. 2010;89:1403–11.

47.Fadl HE, Ostlund IKM, Magnuson AFK, Hanson USB. Maternal and neonatal outcomes and time trends of gestational diabetes mellitus in Sweden from 1991 to 2003. Diabet Med. 2010;27:436–41.
48.Peticca P, Keely EJ, Walker MC, Yang Q, Bottomley J. Pregnancy outcomes in diabetes subtypes: how do they compare? A province-based study of Ontario, 2005-2006. J Obstet Gynaecol Can. 2009;31:487–96.
49.Correa A, Gilboa SM, Besser LM, Botto LD, Moore CA, Hobbs CA, et al. Diabetes mellitus and birth defects. Am J Obstet Gynecol. 2008;199:237.e1–9.
50.Macintosh MC, Fleming KM, Bailey JA, Doyle P, Modder J, Acolet D, et al. Perinatal mortality and congenital anomalies in babies of women with type 1 or type 2 diabetes in England, Wales, and Northern Ireland: population based study. BMJ. 2006;333:177.
51.Yang J, Cummings EA, O'Connell C, Jangaard K. Fetal and neonatal outcomes of diabetic pregnancies. Obstet Gynecol. 2006;108:644–50.
52.Anderson JL, Waller DK, Canfield MA, Shaw GM, Watkins ML, Werler MM. Maternal obesity, gestational diabetes, and central nervous system birth defects. Epidemiology. 2005;16:87–92.
53.Nielsen GL, Nørgard B, Puho E, Rothman KJ, Sørensen HT, Czeizel AE. Risk of specific congenital abnormalities in offspring of women with diabetes. Diabet Med. 2005;22:693–96.
54.Sharpe PB, Chan A, Haan EA, Hiller JE. Maternal diabetes and congenital anomalies in South Australia 1986-2000: a population-based cohort study. Birth Defects Res A Clin Mol Teratol. 2005;73:605–11.
55.Loffredo CA, Wilson PD, Ferencz C. Maternal diabetes: an independent risk factor for major cardiovascular malformations with increased mortality of affected infants. Teratology. 2001;64:98–106.
56.Croen LA, Shaw GM, Lammer EJ. Risk factors for cytogenetically normal holoprosencephaly in California: a population-based case-control study. Am J Med Genet. 2000;90:320–25.
57.von Kries R, Kimmerle R, Schmidt JE, Hachmeister A, Böhm O, Wolf HG. Pregnancy outcomes in mothers with pregestational diabetes: a population-based study in North Rhine (Germany) from 1988 to 1993. Eur J Pediatr. 1997;156:963–67.
58.Janssen PA, Rothman I, Schwartz SM. Congenital malformations in newborns of women with established and gestational diabetes in Washington State, 1984-91. Paediatr Perinat Epidemiol. 1996;10:52–63.
59.Becerra JE, Khoury MJ, Cordero JF, Erickson JD. Diabetes mellitus during pregnancy and the risks for specific birth defects: a population-based case-control study. Pediatrics. 1990;85:1–9.
