# Supplementary material for: Risks of specific congenital anomalies in offspring of women with diabetes: A systematic review and meta-analysis of population-based studies including over 80 million births
Source: PLoS Med. 2022 Feb 1;19(2):e1003900. doi: 10.1371/journal.pmed.1003900 (PMC8806075; doi:10.1371/journal.pmed.1003900)
Supplement: S1 Protocol — (PDF) [file pmed.1003900.s002.pdf]

National Institute for Health Research

UNIVERSITY of York
Centre for Reviews and Dissemination

Systematic review

Fields that have an ***asterisk (*)*** *next to them means that they* ***must be answered. Word limits*** *are provided for each section. You will be unable to submit the form if the word limits are exceeded for any section. Registrant means the person filling out the form.*

1. * Review title.

Give the title of the review in English

Maternal diabetes and risk of specific birth defects in offspring: a systematic review and meta-analysis of population-based studies

2. Original language title.

For reviews in languages other than English, give the title in the original language. This will be displayed with the English language title.

3. * Anticipated or actual start date.

Give the date the systematic review started or is expected to start.
27/12/2020

4. * Anticipated completion date.

Give the date by which the review is expected to be completed.
04/02/2021

5. * Stage of review at time of this submission.

Tick the boxes to show which review tasks have been started and which have been completed. Update this field each time any amendments are made to a published record.

Reviews that have started data extraction (at the time of initial submission) are not eligible for inclusion in PROSPERO. If there is later evidence that incorrect status and/or completion date has been supplied, the published PROSPERO record will be marked as retracted.

This field uses answers to initial screening questions. It cannot be edited until after registration.

The review has not yet started: No

Review stage	Started	Completed
Preliminary searches	Yes	No
Piloting of the study selection process	Yes	No
Formal screening of search results against eligibility criteria	Yes	No
Data extraction	No	No
Risk of bias (quality) assessment	No	No
Data analysis	No	No

Provide any other relevant information about the stage of the review here.

6. * Named contact.

The named contact is the guarantor for the accuracy of the information in the register record. This may be any member of the review team.

Shan-Yan Gao

Email salutation (e.g. "Dr Smith" or "Joanne") for correspondence:

Dr Gao

7. * Named contact email.

Give the electronic email address of the named contact.

gaosy@sj-hospital.org

8. Named contact address

Give the full institutional/organisational postal address for the named contact.

No. 36, San Hao Street, Shenyang, Liaoning, P. R. China

9. Named contact phone number.

Give the telephone number for the named contact, including international dialling code.

86-96615-10027

10. * Organisational affiliation of the review.

Full title of the organisational affiliations for this review and website address if available. This field may be completed as 'None' if the review is not affiliated to any organisation.

Shengjing Hospital of China Medical University

Organisation web address:

http://www.sj-hospital.org/

11. * Review team members and their organisational affiliations.

Give the personal details and the organisational affiliations of each member of the review team. Affiliation refers to groups or organisations to which review team members belong. **NOTE: email and country now MUST be entered for each person, unless you are amending a published record.**

Dr Tie-Ning Zhang. Shengjing Hospital of China Medical University
Dr Xin-Mei Huang. Shanghai Fifth People's Hospital, Fudan University
Dr Xin-Yi Zhao. Shengjing Hospital of China Medical University
Wei Wang. Shengjing Hospital of China Medical University
Ri Wen. Shengjing Hospital of China Medical University
Dr Shan-Yan Gao. Shengjing Hospital of China Medical University

12. * Funding sources/sponsors.

Details of the individuals, organizations, groups, companies or other legal entities who have funded or sponsored the review.

China Postdoctoral Science Foundation funded project (Ref 2019M661178); 345 Talent Project of Shengjing Hospital of China Medical University (Ref M0334). Funding provided by S-YG.

Grant number(s)

State the funder, grant or award number and the date of award

13. * Conflicts of interest.

List actual or perceived conflicts of interest (financial or academic).

None

14. Collaborators.

Give the name and affiliation of any individuals or organisations who are working on the review but who are not listed as review team members. **NOTE: email and country must be completed for each person, unless you are amending a published record.**

15. * Review question.

State the review question(s) clearly and precisely. It may be appropriate to break very broad questions down into a series of related more specific questions. Questions may be framed or refined using PI(E)COS or similar where relevant.

Whether maternal diabetes (i.e, type 1, type 2, or gestational) will increase risk of any birth defects and specific birth defects in offspring?

16. * Searches.

State the sources that will be searched (e.g. Medline). Give the search dates, and any restrictions (e.g. language or publication date). Do NOT enter the full search strategy (it may be provided as a link or attachment below.)

We searched PubMed and Embase from database inception to 27 December 2020. A search strategy combined medical subject heading (MeSH) and Embase subject heading (EMTREE) terms with other unindexed or free text terms, with no limitation on language. Details of the full search strategy are provided in supplementary material. Reference lists of retrieved articles and previous systematic and narrative reviews were searched manually to retrieve all relevant documents. Duplicate citations were removed.

17. URL to search strategy.

Upload a file with your search strategy, or an example of a search strategy for a specific database, (including the keywords) in pdf or word format. In doing so you are consenting to the file being made publicly accessible. Or provide a URL or link to the strategy. Do NOT provide links to your search **results**.

https://www.crd.york.ac.uk/PROSPEROFILES/229217_STRATEGY_20210104.pdf

Alternatively, upload your search strategy to CRD in pdf format. Please note that by doing so you are consenting to the file being made publicly accessible.

Do not make this file publicly available until the review is complete

18. * Condition or domain being studied.

Give a short description of the disease, condition or healthcare domain being studied in your systematic review.

Birth defects in offspring of pregnant women with prepregnancy diabetes or gestational diabetes mellitus.

19. * Participants/population.

Specify the participants or populations being studied in the review. The preferred format includes details of both inclusion and exclusion criteria.

Any birth defects in infants born to mothers with prepregnancy diabetes or gestational diabetes mellitus had a comparison group that included mothers who were without any diabetes.

Birth defect cases attributable to known chromosomal or single-gene disorders were not eligible for the study.

20. * Intervention(s), exposure(s).

Give full and clear descriptions or definitions of the interventions or the exposures to be reviewed. The preferred format includes details of both inclusion and exclusion criteria.

Pregnant women who had prepregnancy diabetes or gestational diabetes mellitus

21. * Comparator(s)/control.

Where relevant, give details of the alternatives against which the intervention/exposure will be compared (e.g. another intervention or a non-exposed control group). The preferred format includes details of both inclusion and exclusion criteria.

Pregnant women who were without any diabetes.

22. * Types of study to be included.

Give details of the study designs (e.g. RCT) that are eligible for inclusion in the review. The preferred format includes both inclusion and exclusion criteria. If there are no restrictions on the types of study, this should be stated.

Population-based studies that reported original data were eligible for inclusion if they reported any birth defects in infants born to mothers with prepregnancy diabetes or gestational diabetes mellitus had a comparison group that included mothers who were without any diabetes, if a risk estimate was not reported, provided enough data from which a risk estimate could be calculated. All conference abstracts, guidelines, dissertations, commentaries, and letters were excluded. Birth defect cases attributable to known chromosomal or single-gene disorders were not eligible for the study.

23. Context.

Give summary details of the setting or other relevant characteristics, which help define the inclusion or exclusion criteria.

24. * Main outcome(s).

Give the pre-specified main (most important) outcomes of the review, including details of how the outcome is defined and measured and when these measurement are made, if these are part of the review inclusion criteria.

Cardiac birth defects (including septal defects, atrial septal defect, ventricular septal defect, left ventricular outflow tract defects, and right ventricular outflow tract defects) in offspring.

* Measures of effect

Please specify the effect measure(s) for you main outcome(s) e.g. relative risks, odds ratios, risk difference, and/or 'number needed to treat.

Risk estimate (i.e., odds ratio, risk ratio, or relative risk) with 95% confidence intervals.

25. * Additional outcome(s).

List the pre-specified additional outcomes of the review, with a similar level of detail to that required for main outcomes. Where there are no additional outcomes please state 'None' or 'Not applicable' as appropriate to the review

Specific birth defects (including nervous system, eye, ear face and neck, respiratory system, orofacial cleft, digestive system, urogenital system, urinary system, genital system, musculoskeletal system, limb, and abdominal wall defects) in offspring.

* Measures of effect

Please specify the effect measure(s) for you additional outcome(s) e.g. relative risks, odds ratios, risk difference, and/or 'number needed to treat.

Risk estimate (i.e., odds ratio, risk ratio, or relative risk) with 95% confidence intervals.

26. * Data extraction (selection and coding).

Describe how studies will be selected for inclusion. State what data will be extracted or obtained. State how this will be done and recorded.

A standardized, pre-designed spreadsheet was used for data extraction from the included studies. The study quality and synthesis of evidence were assessed. The following data were extracted into the spreadsheet: first author, publication year, geographic location, study period, data source, sample size (cases and cohorts), types of birth, definition of outcome, outcome with their risk estimates and 95% confidence intervals (CIs), and adjusted confounders. Two authors (T-NZ and S-YG) independently undertook data extraction according to The Cochrane Handbook guidelines, and findings were reported according to PRISMA and MOOSE guidance. Any disagreement was settled by consensus among all authors. For studies that did not report any adjusted effect sizes, we used the crude risk estimate. If an included study reported several risk estimates, we extracted the fully adjusted effect sizes. Because the odds ratio is an excellent approximation of the risk ratio in the case of rare outcomes, the results were referred to as relative risks (RRs); therefore, all

results were reported as RRs for simplicity. If a study lacked required data, they were requested by contacting the study authors by email.

27. * Risk of bias (quality) assessment.

State which characteristics of the studies will be assessed and/or any formal risk of bias/quality assessment tools that will be used.

We used the Newcastle-Ottawa scale to assess the risk of bias of cohort and case-control studies, which included studies consists of three categories: selection of study participant groups (four items, one point each), the comparability of study groups (one item, up to two points), and the ascertainment of outcome (three items, one point each), up to a maximum score of nine points. Studies were considered to have low risk of bias if they achieved a full rating in at least two categories of selection, comparability, or outcome assessment.

28. * Strategy for data synthesis.

Describe the methods you plan to use to synthesise data. This **must not be generic text** but should be **specific to your review** and describe how the proposed approach will be applied to your data. If meta-analysis is planned, describe the models to be used, methods to explore statistical heterogeneity, and software package to be used.

For studies that reported effect sizes separately; here, the results were pooled using a fixed effects model to obtain an overall estimate then included the pooled effect size in the meta-analysis. The effective count method proposed by Hamling et al. was used to recalculate the effect sizes. If the selected study did not include a effect size, the unadjusted risk estimate and the 95% CI were calculated from the raw data for simplicity by using EpiCalc-2000 (https://en.freedownloadmanager.org/Windows-PC/EpiCalc-2000-FREE.html). Estimates were pooled using the DerSimonian and Laird random-effects model to calculate summarized RRs and 95% CI. To pool study results, random effects models were used for analyses, in which we calculated I^2 as indicators of heterogeneity. I^2 values greater than 50% were considered to indicate signifcant heterogeneity between the included studies. Heterogeneity between subgroups was evaluated by meta-regression analysis if the data were reported in more than 10 studies, according to the Cochrane guidelines. Publication bias was examined by inspecting funnel plots for the outcomes, and further tested with Begg’ s test and Egger’ s test. A sensitivity analysis was undertaken to explore the effect of each individual study on the overall pooled estimate. Statistical analyses were conducted using Stata version 13.0 (StataCorp, College Station, Texas). A two-tailed P-value less than 0.05 was considered as statistically significant.

29. * Analysis of subgroups or subsets.

State any planned investigation of ‘subgroups’. Be clear and specific about which type of study or participant will be included in each group or covariate investigated. State the planned analytic approach.

We will explore potential sources of heterogeneity by conducting subgroup analyses according to: study

quality, geographic location, type of study (i.e., cross-sectional, case-control, and cohort), and adjustment for potential confounders (e.g., maternal age, socioeconomic status, smoking or alcohol drinking, pregnancy body mass index [BMI], pregnancy complications, and parity).

30. * Type and method of review.

Select the type of review, review method and health area from the lists below.

Type of review

Cost effectiveness
No

Diagnostic
No

Epidemiologic
Yes

Individual patient data (IPD) meta-analysis
No

Intervention
No

Meta-analysis
Yes

Methodology
No

Narrative synthesis
No

Network meta-analysis
No

Pre-clinical
No

Prevention
No

Prognostic
No

Prospective meta-analysis (PMA)
No

Review of reviews
No

Service delivery
No

Synthesis of qualitative studies
No

Systematic review
Yes

Other

No

Health area of the review

Alcohol/substance misuse/abuse
No

Blood and immune system
No

Cancer
No

Cardiovascular
Yes

Care of the elderly
No

Child health
No

Complementary therapies
No

COVID-19
No

Crime and justice
No

Dental
No

Digestive system
No

Ear, nose and throat
No

Education
No

Endocrine and metabolic disorders
Yes

Eye disorders
No

General interest
No

Genetics
No

Health inequalities/health equity
No

Infections and infestations
No

International development
No

Mental health and behavioural conditions
No

Musculoskeletal
No

Neurological
No

Nursing
No

Obstetrics and gynaecology
No

Oral health
No

Palliative care
No

Perioperative care
No

Physiotherapy
No

Pregnancy and childbirth
Yes

Public health (including social determinants of health)
No

Rehabilitation
No

Respiratory disorders
No

Service delivery
No

Skin disorders
No

Social care
No

Surgery
No

Tropical Medicine
No

Urological
No

Wounds, injuries and accidents
No

Violence and abuse
No

31. Language.

Select each language individually to add it to the list below, use the bin icon to remove any added in error.
English

There is not an English language summary

32. * Country.

Select the country in which the review is being carried out. For multi-national collaborations select all the countries involved.
China

33. Other registration details.

Name any other organisation where the systematic review title or protocol is registered (e.g. Campbell, or The Joanna Briggs Institute) together with any unique identification number assigned by them. If extracted data will be stored and made available through a repository such as the Systematic Review Data Repository (SRDR), details and a link should be included here. If none, leave blank.

34. Reference and/or URL for published protocol.

If the protocol for this review is published provide details (authors, title and journal details, preferably in Vancouver format)

Add web link to the published protocol.

Or, upload your published protocol here in pdf format. Note that the upload will be publicly accessible.

No I do not make this file publicly available until the review is complete

Please note that the information required in the PROSPERO registration form must be completed in full even if access to a protocol is given.

35. Dissemination plans.

Do you intend to publish the review on completion?

Yes

Give brief details of plans for communicating review findings.?

36. Keywords.

Give words or phrases that best describe the review. Separate keywords with a semicolon or new line. Keywords help PROSPERO users find your review (keywords do not appear in the public record but are included in searches). Be as specific and precise as possible. Avoid acronyms and abbreviations unless these are in wide use.

Systematic review; meta-analysis; birth defect; gestational diabetes mellitus; maternal diabetes; population-based study

37. Details of any existing review of the same topic by the same authors.

If you are registering an update of an existing review give details of the earlier versions and include a full bibliographic reference, if available.

38. * Current review status.

Update review status when the review is completed and when it is published.New registrations must be

ongoing so this field is not editable for initial submission.
Please provide anticipated publication date

Review_Ongoing

39. Any additional information.

Provide any other information relevant to the registration of this review.

40. Details of final report/publication(s) or preprints if available.

Leave empty until publication details are available OR you have a link to a preprint (NOTE: this field is not editable for initial submission). List authors, title and journal details preferably in Vancouver format.

Give the link to the published review or preprint.
