# Supplementary material for: Risks of specific congenital anomalies in offspring of women with diabetes: A systematic review and meta-analysis of population-based studies including over 80 million births
Source: PLoS Med. 2022 Feb 1;19(2):e1003900. doi: 10.1371/journal.pmed.1003900 (PMC8806075; doi:10.1371/journal.pmed.1003900)
Supplement: S1 Table — (DOCX) [file pmed.1003900.s003.docx]

**S1 Table**

This supporting information formed part of the original submission and has been peer reviewed.

We post it as supplied by the authors.

Supplement to: Tie-Ning Zhang, Xin-Mei Huang, Xin-Yi Zhao, Wei Wang, Ri Wen, Shan-Yan Gao.

Risks of specific congenital anomalies in offspring of women with diabetes: A systematic review and meta-analysis of population-based studies including over 80 million births

| **S1 Table. EUROCAT, ICD-10 and ICD-9 codes used to identify and define congenital anomalies.** | | | | |
| --- | --- | --- | --- | --- |
| **Types of congenital anomalies** | **EUROCAT subgroup** | ***ICD-10* codes** | ***ICD-9* codes** | **Defination of included studies** |
| **Congenital anomalies** | All Anomalies: al1 | Congenital malformations: Q-chapter, D215, D821, D1810, P350, P351, P371 | 74, 75, 27910, 2281, 7710, 7711, 77121, 76280 | Yang et al., 2019 [1]: Any birth defect was defined as having any least one of above twelve birth defects, including multiple birth defects. Feig et al., 2014 [2]: any congenital anomaly with ICD-9 diagnosis codes 740 to 759 or any ICD-10 diagnosis codes Q00.0 to Q99.9 (including cardiac, neural tube defect, central nervous system, gastrointestinal, and renal anomalies).  Correa et al., 2008 [3]: All cases with heart defects were classified further about whether the heart defect was simple (ie, 1 well-recognized entity such as atrial septal defect or tetralogy of Fallot with no other cardiac defects), complex (ie, heterotaxy and single ventricle malformations), or an association of 2 heart defects neither of which could be considered the primary defect for analysis (eg, atrial septal defect with ventricular septal defect). |
| **Major congenital anomalies** | N/A | N/A | N/A | Bayoumi et al., 2021 [4]: major congenital malformations (cardiovascular, central nervous system defects or gastrointestinal anomalies).  Yang et al., 2006 [5]: Major congenital anomaly was defined in the database as one which is any of the following: lethal, life-shortening, life threatening, requires major surgery, or affecting in a significant way the quality of life. |
| **Congenital heart defects** | Congenital heart defects: al17 | Q20-Q26 | 745, 746, 7470-7474 | Arendt et al., 2021 [6]: ICD-10 Q20-Q25, Q260, Q262-Q269. Q250 and Q256 not included if GA <37 weeks .  Ludvigsson et al., 2018 [7]: Q20-Q25, Q260, Q262-Q269.  Liu et al., 2015 [8]: congenital heart disease was defined as a structural abnormality of the heart or great vessels as shown by  echocardiography. |
| **Heterotaxia** | N/A | N/A | N/A | Leirgul et al., 2016 [9]: Situs inversus, lsomerism, and dextrocardia or levocardia with other congenital heart defect. Liu et al., 2013 [10]: heterotaxia (abnormalities in left-right cardiac asymmetry associated with intracardiac defects such as an atrioventricular septal defect, abnormalities of systemic and pulmonary venous drainage, malposition of the great vessels, and subpulmonary or aortic obstruction). |
| **Conotruncal defects** | N/A | N/A | N/A | Leirgul et al., 2016 [9]: transposition of the great arteries, Tetralogy of Fallot, pulmonary atresia with ventricular septal defect (Tetralogy of Fallot type), double outlet right ventricle, conoventricular septal defect, interrupted aortic arch type B or C, supravalvular aortic stenosis, and aortopulmonary window. |
| **Truncus arteriosus** | N/A | N/A | N/A |  |
| **Transposition of great vessels** | Transposition of great vessels (complete): al19 | Q203 | 74510 |  |
| **Tetralogy of Fallot** | Tetralogy of Fallot: al24 | Q213 | 7452 |  |
| **Atrioventricular septal defect** | AVSD: al23 | Q212 | 7456 |  |
| **Anomalous pulmonary venous return** | Total anomalous pulm venous return: al33 | Q262 | Total anomalous pulmonary venous return: 74741 | Leirgul et al., 2016 [9]: total anomalous pulmonary venous return and partial anomalous pulmonary venous return. |
| **LVOT** | N/A | N/A | N/A | Leirgul et al., 2016 [9]: hypoplastic left heart syndrome, mitral valve stenosis, coarctation of the aorta, interrupted aortic arch type A, and valvular aortic stenosis. Csaky-Szunyogh et al., 2014 [11]: congenital stenosis of the aortic valve, aortic valve stenosis, hypoplastic left heart, coarctation of aorta, other congenital anomalies of the Aorta. Correa et al., 2008 [3]: coarctation of the aorta with aortic stenosis, coarctation of the aorta with VSD, and coarctation of the aorta with VSD and ASD. |
| **Coarctation of aorta** | Coarctation of aorta: al32 | Q251 | 7471 |  |
| **Hypoplastic left heart** | Hypoplastic left heart: al30 | Hypoplastic left heart syndrome: Q234 | 7467 |  |
| **RVOT** | N/A | N/A | N/A | Leirgul et al., 2016 [9]: tricuspid atresia or stenosis, hypoplastic right heart syndrome, ebstein anomaly, valvular pulmonary atresia (not Tetralogy of Fallot anatomy), arterial pulmonary atresia, and valvular pulmonary stenosis.  Correa et al., 2008 [3]: pulmonary valvestenosis with VSD and pulmonary valve stenosis with ASD. |
| **Pulmonary artery anomalies** | Pulmonary valve atresia: al28 | Pulmonary valve atresia: Q220 | Pulmonary valve anomaly, unspecified: 74600 |  |
| **Pulmonary valve stenosis** | Pulmonary valve stenosis: al27 | Q221 | 74601 | Liu et al., 2015 [8]: Pulmonary stenosis was defined as having an obstruction (increased velocity > 2.0 mm/s) to outflow from the right ventricle of the heart to the pulmonary artery. |
| **Septal defects** | N/A | Q21 | 745 |  |
| **Ventricular septal defect** | VSD: al21 | Q210 | 7454 | Liu et al., 2015 [8]: Ventricular septal defect was defined as having a defect between the left and right ventricles at the interventricular septal level. |
| **Atrial septal defect** | ASD: al22 | Q211 | 7455 | Liu et al., 2015 [8]: Atrial septal defect was defined as having a defect and shunt between the left and right atria at the interatria septal level except for patent foramen oval (PFO, defect diameter at the oval fossa < 5 mm). |
| **Single ventricle** | Single ventricle: al20 | Q204 | 7453 | Liu et al., 2015 [8]: Single ventricle was defined as the presence of two atrioventricular valves with one ventricular chamber or a large dominant ventricle associated with a diminutive opposing ventricle. |
| **Nervous system** | Nervous system: al2 | Q00-Q07 | 740-742 | Arendt et al., 2021 [6]: ICD-10 Q00–Q07. Minor anomalies excluded: Q0461, Q0782.  Billionnet et al., 2017 [12]: congenital malformations of the nervous system (ICD-10 Q00–Q07). |
| **Neural tube defects** | Neural tube defect: al3 | Q00, Q01, Q05 | 740, 741, 7420 |  |
| **Anencephaly** | Anencephalus and similar: al4 | Anencephaly and similar malformations: Q00 | 740 | Liu et al., 2019 [13]: anencephaly (Q00.0‐Q00.2, including acrania, craniorachischisis, and exencephaly) on ICD -10 codes. |
| **Encephalocele** | Encephalocele: al5 | Q01 | 7420 | Liu et al., 2019 [13]: encephalocele (Q01.0-Q01.2, Q01.8, and Q01.9) on ICD -10 codes. |
| **Spina bifida** | Spina bifida: al6 | Q05 | 741 | Schraw et al.,2021 [14]: spina bifida (collapsed to the first three digits to combine spina bifida with and without hydrocephaly). Liu et al., 2019 [13]: spina bifida or meningomyelocele (Q05.0-Q05.9) on ICD -10 codes. |
| **Hydrocephaly** | Hydrocephaly: al7 | Congenital hydrocephalus: Q03 | 7423, Exclude 74232 |  |
| **Holoprosencephaly** | Arhinencephaly/holoprosencephaly: al9 | Holoprosencephaly: Q042 | 74226 |  |
| **Eye, ear, face and neck** | Eye: al10 | Eye: Q10-Q15 | Eye: 743 | Arendt et al., 2021 [6]: ICD-10 Q100, Q104, Q106-Q109, Q11-Q12, Q130-Q134, Q136-Q139, Q14-Q15. Minor anomalies excluded: Q101-Q103, Q105, Q135. |
|  | Ear, face and neck: al15 | Ear, face and neck: Q16-Q18 | Ear, face, and neck: 744 | Arendt et al., 2021 [6]: ICD-10 Q16, Q176-Q178, Q183, Q188, Q189. Minor anomalies excluded: Q170-Q175, Q179, Q180- Q182, Q184-Q187. |
| **Orofacial cleft** | Oro-facial clefts: al101 | Cleft lip and cleft palate: Q35-Q37 | 7490, 7491, 7492 |  |
| **Cleft palate** | Cleft palate: al103 | Q35 | 7490 |  |
| **Cleft lip with or without cleft** | Cleft lip with or without cleft: al102 | Q36, Q37 | Cleft lip: 7491 Cleft palate with cleft lip: 7492 | Schraw et al.,2021 [14]: cleft lip alone (7491) and cleft lip with cleft palate (7492) which were both assigned 7491 - cleft lip with or without cleft palate on ICD-9 codes. |
| **Digestive system** | Digestive system: al40 | Q38-Q45, Q790 | 749, 750, 751 | Arendt et al., 2021 [6]: ICD-10 Q38-Q45, Q790. Minor anomalies excluded: Q381, Q382, Q3850, Q400, Q401, Q4021, Q430, Q4320, Q4381, Q4382, Q385A. |
| **Diaphragmatic hernia** | Diaphragmatic hernia: al48 | Q790 | 75661 |  |
| **Abdominal wall defects** | Abdominal wall defects: al49 | Q792, Q793, Q795 | Congenital anomalies of abdominal wall: 7567 |  |
| **Omphalocele** | Omphalocele: al51 | Q792 | 7567 |  |
| **Gastroschisis** | Gastroschisis: al50 | Q793 | 75671 |  |
| **Genitourinary** | Genital: al58 | Genital organs: Q50-Q56 | Genital organs: 752 | Arendt et al., 2021 [6]: ICD-10 Q50-Q52, Q54-Q56. Minor anomalies excluded: Q53, Q523, Q525, Q527, Q552A, Q552B, Q552E, Q552F. |
|  | Urinary: al52 | Urinary system: Q60-Q64 | Urinary system: 753 | Arendt et al., 2021 [6]: ICD-10 Q60-Q64, Q794. Minor anomalies excluded: Q610, Q627, Q633. |
| **Renal agenesis/dysgenesis** | Renal Dysplasia: al54 | Renal dysplasia: Q614 | 75316 |  |
| **Hypospadias** | Hypospadias: al59 | Q54 | 75261 | Arendt et al., 2018 [6]: Hypospadias were defined based on the following diagnostic codes; ICD-8: 75220, 75221, 75222, 75228, 75229, ICD-9: 752G and ICD-10: Q540, Q540A, Q541, Q542, Q543, Q548 and Q549. |
| **Musculoskeletal system** | N/A | Congenital malformations and deformations of the musculoskeletal system: Q65-Q79 | 754, 755, 756 |  |
| **Limb reduction** | Limb reduction: al62 | Q71-Q73 | 7552-7554, 7556 | Yang et al., 2019 [1]: Limb reduction defect was defined if any of the following conditions had been diagnosed by a physician: a missing hand, arm, foot, or leg, or any portion of it, excluding congenital amputation and dwarfing syndromes. |
| **Poly/syndactyly** | Polydactyly: al68 | Q69 | 7550 |  |
|  | Syndactyly: al69 | Q70 | 7551 |  |
| Abbreviations: EUROCAT, European Surveillance of Congenital Anomalies; ICD-8, International Classification of Diseases, Eighth Revision; ICD-9, International Classification of Diseases, Ninth Revision; ICD-10, International Classification of Diseases, Tenth Revision; LVOT, Left ventricular outflow tract defects; N/A, Not available; RVOT, Right ventricular outflow tract defects. | | | | |

References

1.Yang G-R, Dye TD, Li D. Effects of pre-gestational diabetes mellitus and gestational diabetes mellitus on macrosomia and birth defects in Upstate New York. Diabetes Res Clin Pract. 2019;155:107811.

2.Feig DS, Hwee J, Shah BR, Booth GL, Bierman AS, Lipscombe LL. Trends in incidence of diabetes in pregnancy and serious perinatal outcomes: a large, population-based study in Ontario, Canada, 1996–2010. Diabetes Care. 2014;37:1590–96.

3.Correa A, Gilboa SM, Besser LM, Botto LD, Moore CA, Hobbs CA, et al. Diabetes mellitus and birth defects. Am J Obstet Gynecol. 2008;199:237.e1–9.

4.Bayoumi MAA, Masri RM, Matani NYS, Hendaus MA, Masri MM, Chandra P, et al. Maternal and neonatal outcomes in mothers with diabetes mellitus in qatari population.BMC Pregnancy Childbirth. 2021;21(1):651.

5.Yang J, Cummings EA, O'Connell C, Jangaard K. Fetal and neonatal outcomes of diabetic pregnancies. Obstet Gynecol. 2006;108:644–50.1

6.Arendt LH, Pedersen LH, Pedersen L, Ovesen PG, Henriksen TB, Lindhard MS, et al. Glycemic Control in Pregnancies Complicated by Pre-Existing Diabetes Mellitus and Congenital Malformations: A Danish Population-Based Study. Clin Epidemiol. 2021;13:615–26.

7.Ludvigsson JF, Neovius M, Söderling J, Gudbjörnsdottir S, Svensson AM, Franzén S, et al. Periconception glycaemic control in women with type 1 diabetes and risk of major birth defects: population based cohort study in Sweden. BMJ. 2018;362:k2638.

8.Liu X, Liu G, Wang P, Huang Y, Liu E, Li D, et al. Prevalence of congenital heart disease and its related risk indicators among 90,796 Chinese infants aged less than 6 months in Tianjin. Int J Epidemiol. 2015;44:884–93.

9.Leirgul E, Brodwall K, Greve G, Vollset SE, Holmstrøm H, Tell GS, et al. Maternal Diabetes, Birth Weight, and Neonatal Risk of Congenital Heart Defects in Norway, 1994–2009. Obstet Gynecol. 2016;128:1116–25.

10.Liu S, Joseph KS, Lisonkova S, Rouleau J, Van den Hof M, Sauve R, et al. Association between maternal chronic conditions and congenital heart defects: a population-based cohort study. Circulation. 2013;128:583–89.

11.Csáky-Szunyogh M, Vereczkey A, Kósa Z, Gerencsér B, Czeizel AE. Risk factors in the origin of congenital left-ventricular outflow-tract obstruction defects of the heart: a population-based case-control study. Pediatr Cardiol. 2014;35:108–20.

12.Billionnet C, Mitanchez D, Weill A, Nizard J, Alla F, Hartemann A, et al. Gestational diabetes and adverse perinatal outcomes from 716,152 births in France in 2012. Diabetologia. 2017;60:636–44.

13.Liu S, Evans J, MacFarlane AJ, Ananth CV, Little J, Kramer MS, et al. Association of maternal risk factors with the recent rise of neural tube defects in Canada. Paediatr Perinat Epidemiol. 2019;33:145–53.

14.Schraw JM, Langlois PH, Lupo PJ. Comprehensive assessment of the associations between maternal diabetes and structural birth defects in offspring: a phenome-wide association study. Ann Epidemiol. 2021;53:14–20.e8.
