# Supplementary material for: Risks of specific congenital anomalies in offspring of women with diabetes: A systematic review and meta-analysis of population-based studies including over 80 million births
Source: PLoS Med. 2022 Feb 1;19(2):e1003900. doi: 10.1371/journal.pmed.1003900 (PMC8806075; doi:10.1371/journal.pmed.1003900)
Supplement: S2 Table — (DOCX) [file pmed.1003900.s004.docx]

**S2 Table**

This supporting information formed part of the original submission and has been peer reviewed.

We post it as supplied by the authors.

Supplement to: Tie-Ning Zhang, Xin-Mei Huang, Xin-Yi Zhao, Wei Wang, Ri Wen, Shan-Yan Gao.

Risks of specific congenital anomalies in offspring of women with diabetes: A systematic review and meta-analysis of population-based studies including over 80 million births

| **S2 Table. References of studies excluded in the systematic review and meta-analysis of population-based studie**s |
| --- |
| **Mothers with any diabetes not distinguish pre-gestational or gestational (n = 3)** |
| 1. Munch TN, Rasmussen ML, Wohlfahrt J, Juhler M, Melbye M. Risk factors for congenital hydrocephalus: a nationwide, register-based, cohort study. J Neurol Neurosurg Psychiatry. 2014;85(11):1253-9. |
| 2. Spilson SV, Kim HJ, Chung KC. Association between maternal diabetes mellitus and newborn oral cleft. Ann Plast Surg. 2001;47(5):477-81. |
| 3. Ghimire LV, Croce N. Socioeconomic and racial disparities in the risk of congenital anomalies in infants of diabetic mothers: A national population-based study. Early Hum Dev. 2020;151:105195. |
| **Not have a comparison group that included mothers without diabetes (n = 1)** |
| 4. Murphy HR, Howgate C, O'Keefe J, et al. National Pregnancy in Diabetes (NPID) advisory group. Characteristics and outcomes of pregnant women with type 1 or type 2 diabetes: a 5-year national population-based cohort study. Lancet Diabetes Endocrinol. 2021;9(3):153-164. |
| **Duplication (n = 4)** |
| 5. Newham JJ, Glinianaia SV, Tennant PW, Rankin J, Bell R. Improved antenatal detection of congenital anomalies in women with pre-gestational diabetes: population-based cohort study. Diabet Med. 2013;30(12):1442-8.^*^ |
| 6. Tinker SC, Gilboa SM, Moore CA, et al. National Birth Defects Prevention Study. Modification of the association between diabetes and birth defects by obesity, National Birth Defects Prevention Study, 1997-2011. Birth Defects Res. 2021;113(14):1084-1097. ^†^ |
| 7. Nalbandyan M, Howley MM, Cunniff CM, Romitti PA, Browne ML, National Birth Defects Prevention Study. Descriptive and risk factor analysis of nonsyndromic sacral agenesis: National Birth Defects Prevention Study, 1997-2011. Am J Med Genet A. 2019;179(9):1799-1814.^†^ |
| 8. Miller EA, Rasmussen SA, Siega-Riz AM, Frías JL, Honein MA, National Birth Defects Prevention Study. Risk factors for non-syndromic holoprosencephaly in the National Birth Defects Prevention Study. Am J Med Genet C Semin Med Genet. 2010;154C(1):62-72.^†^ |
| **Not population-based studies (n = 27)** |
| 9. Ali DS, Davern R, Rutter E, et al. Pre-Gestational Diabetes and Pregnancy Outcomes. Diabetes Ther. 2020;11(12):2873-2885. |
| 10. Nakanishi K, Kanagawa T, Fujikawa K, Ishii K, Waguri M. Congenital malformation and hemoglobin A1c in the first trimester among Japanese women with pregestational diabetes. J Obstet Gynaecol Res. 2021. |
| 11. Martin RB, Duryea EL, Ambia A, et al. Congenital Malformation Risk According to Hemoglobin A1c Values in a Contemporary Cohort with Pregestational Diabetes. Am J Perinatol. 2021;38(12):1217-1222. |
| 12. Alyousif SMM, Aldokhel FT, Alkhanbashi OK, et al. The Incidence of Congenital Heart Defects in Offspring Among Women With Diabetes in Saudi Arabia. Cureus. 2021;13(3):e14225. |
| 13. Dude AM, Badreldin N, Schieler A, Yee LM. Periconception glycemic control and congenital anomalies in women with pregestational diabetes. BMJ Open Diabetes Res Care. 2021;9(1):e001966. |
| 14. Riskin A, Itzchaki O, Bader D, Iofe A, Toropine A, Riskin-Mashiah S. Perinatal Outcomes in Infants of Mothers with Diabetes in Pregnancy. Isr Med Assoc J. 2020;22(9):569-575. |
| 15. Martínez-Frías ML, Bermejo E, Rodríguez-Pinilla E, Prieto L, Frías JL. Epidemiological analysis of outcomes of pregnancy in gestational diabetic mothers. Am J Med Genet. 1998;78(2):140-5. |
| 16. Evers IM, de Valk HW, Visser GH. Risk of complications of pregnancy in women with type 1 diabetes: nationwide prospective study in the Netherlands. BMJ. 2004;328(7445):915. |
| 17. Agha MM, Glazier RH, Moineddin R, Booth G. Congenital abnormalities in newborns of women with pregestational diabetes: A time-trend analysis, 1994 to 2009. Birth Defects Res A Clin Mol Teratol. 2016;106(10):831-839. |
| 18. Beyerlein A, Lack N, von Kries R. No further improvement in pregnancy-related outcomes in the offspring of mothers with pre-gestational diabetes in Bavaria, Germany, between 2001 and 2016. Diabet Med. 2018;35(10):1420-1424. |
| 19. Luo L, Huang P, Wang TT, et al. [Association of maternal diabetes mellitus and UCP2 gene polymorphisms with congenital heart disease in offspring: a case-control study]. Zhongguo Dang Dai Er Ke Za Zhi. 2020;22(10):1092-1099. |
| 20. Giraldo-Grueso M , Zarante I , Alejandro Mejía-Grueso, et al. Risk factors for congenital heart disease: A case-control study. Revista Colombiana de Cardiologia. 2020;27(4). |
| 21. Pascon T, Barbosa AMP, Cordeiro RCL, et al. Prenatal exposure to gestational diabetes mellitus increases developmental defects in the enamel of offspring. PLoS One. 2019;14(2):e0211771. |
| 22. Abell SK, Boyle JA, de Courten B, et al. Impact of type 2 diabetes, obesity and glycaemic control on pregnancy outcomes. Aust N Z J Obstet Gynaecol. 2017;57(3):308-314. |
| 23. Akbariasbagh P, Shariat M, Akbariasbagh N, Ebrahim B. Cardiovascular Malformations in Infants of Diabetic Mothers: A Retrospective Case-Control Study. Acta Med Iran. 2017;55(2):103-108. |
| 24. Lasheen AE, Abdelbasit OB, Seidahmed MZ, Hussein KA, Miqdad AM, Al Zahrani MH, Farid GM, Badr HA. Infants of diabetic mothers. A cohort study. Saudi Med J. 2014;35(6):572-7. |
| 25. Tundidor D, García-Patterson A, María MA, et al. Perinatal maternal and neonatal outcomes in women with gestational diabetes mellitus according to fetal sex. Gend Med. 2012;9(6):411-7. |
| 26. Beyerlein A, von Kries R, et al. Improvement in pregnancy-related outcomes in the offspring of diabetic mothers in Bavaria, Germany, during 1987-2007. Diabet Med. 2010;27(12):1379-84. |
| 27. Abolfazl M , Hamidreza T S , Narges M Y , et al. Gestational diabetes and its association with unpleasant outcomes of pregnancy. Pakistan Journal of Medical Sciences Online. 2008;24(4):566-570. |
| 28. Jazmín Arteaga, Luna L, Mutchinick O M. Diabets, pregnancy and birth defects. Revista de investigacion clinica; organo del Hospital de Enfermedades de la Nutricion. 2008;60(2):107-114. |
| 29. Jaime L. Frías, Juan P. Frías, Patricio A. Frías, et al. Infrequently studied congenital anomalies as clues to the diagnosis of maternal diabetes mellitus. Am J Med Genet A. 2010, 143(24):2904-2909. |
| 30. Frías JL, Frías JP, Frías PA, Martínez-Frías ML. Infrequently studied congenital anomalies as clues to the diagnosis of maternal diabetes mellitus. Am J Med Genet A. 2007;143A(24):2904-9. |
| 31. Ray JG, Vermeulen MJ, Meier C, Wyatt PR. Risk of congenital anomalies detected during antenatal serum screening in women with pregestational diabetes. QJM. 2004;97(10):651-3. |
| 32. Farrell T, Neale L, Cundy T. Congenital anomalies in the offspring of women with type 1, type 2 and gestational diabetes. Diabet Med. 2002;19(4):322-6. |
| 33. Sheffield JS, Butler-Koster EL, Casey BM, McIntire DD, Leveno KJ. Maternal diabetes mellitus and infant malformations. Obstet Gynecol. 2002;100(5 Pt 1):925-30. |
| 34. Martínez-Frías ML. Heterotaxia as an outcome of maternal diabetes: an epidemiological study. Am J Med Genet. 2001;99(2):142-6. |
| 35. Moore LL, Singer MR, Bradlee ML, Rothman KJ, Milunsky A. A prospective study of the risk of congenital defects associated with maternal obesity and diabetes mellitus. Epidemiology. 2000;11(6):689-94. |
| **Enrolled mothers with congenital anomalies (n = 3)** |
| 36. Svenningsson A, Gunnarsdottir A, Wester T. Maternal risk factors and perinatal characteristics of anorectal malformations. J Pediatr Surg. 2018;53(11):2183-2188. |
| 37. Zwink N, Rissmann A, Pötzsch S, Reutter H, Jenetzky E, CURE-Net Consortium. Parental risk factors of anorectal malformations: Analysis with a regional population-based control group. Birth Defects Res A Clin Mol Teratol. 2016;106(2):133-41. |
| 38. Groen In 't Woud S, Renkema KY, Schreuder MF, Wijers CH, van der Zanden LF, Knoers NV, Feitz WF, Bongers EM, Roeleveld N, van Rooij IA. Maternal risk factors involved in specific congenital anomalies of the kidney and urinary tract: A case-control study. Birth Defects Res A Clin Mol Teratol. 2016;106(7):596-603. |
| **Data could not be extracted or calculated (n = 6)** |
| 39. Pylypjuk C, Sellers E, Wicklow B. Perinatal Outcomes in a Longitudinal Birth Cohort of First Nations Mothers With Pregestational Type 2 Diabetes and Their Offspring: The Next Generation Study. Can J Diabetes. 2021;45(1):27-32. |
| 40. Chen LJ, Chiou JY, Huang JY, Su PH, Chen JY. Birth defects in Taiwan: A 10-year nationwide population-based, cohort study. J Formos Med Assoc. 2020;119(1 Pt 3):553-559. |
| 41. Ferencz C, Rubin JD, McCarter RJ, Clark EB. Maternal diabetes and cardiovascular malformations: predominance of double outlet right ventricle and truncus arteriosus. Teratology. 1990;41(3):319-26. |
| 42. Orbain MM, Johnson J, Nance A, et al. Maternal diabetes-related malformations in Utah: A population study of birth prevalence 2001-2016. Birth Defects Res. 2020. |
| 1. Hautala J, Gissler M, Ritvanen A, et al. The implementation of a nationwide anomaly screening programme improves prenatal detection of major cardiac defects: an 11-year national population-based cohort study. BJOG. 2019;126(7):864-873. 2. Borsari L, Malagoli C, Werler MMs, et al. Joint Effect of Maternal Tobacco Smoking and Pregestational Diabetes on Preterm Births and Congenital Anomalies: A Population-Based Study in Northern Italy. J Diabetes Res. 2018;2018:2782741. |
| * Study duplicated the citation (Bell et al., 2012 [1]). † Studies duplicated the citation (Tinker et al., 2020 [2]). |

References

1. Bell R, Glinianaia SV, Tennant PWG, Bilous RW, Rankin J. Peri-conception hyperglycaemia and nephropathy are associated with risk of congenital anomaly in women with pre-existing diabetes: a population-based cohort study. Diabetologia. 2012;55:936–47.
2. Tinker SC, Gilboa SM, Moore CA, Waller DK, Simeone RM, Kim SY, et al. Specific birth defects in pregnancies of women with diabetes: National Birth Defects Prevention Study, 1997–2011. Am J Obstet Gynecol. 2020;222:176.e1–11.
