# Supplementary material for: Risks of specific congenital anomalies in offspring of women with diabetes: A systematic review and meta-analysis of population-based studies including over 80 million births
Source: PLoS Med. 2022 Feb 1;19(2):e1003900. doi: 10.1371/journal.pmed.1003900 (PMC8806075; doi:10.1371/journal.pmed.1003900)
Supplement: S3 Table — (DOCX) [file pmed.1003900.s005.docx]

**S3 Table**

This supporting information formed part of the original submission and has been peer reviewed.

We post it as supplied by the authors.

Supplement to: Tie-Ning Zhang, Xin-Mei Huang, Xin-Yi Zhao, Wei Wang, Ri Wen, Shan-Yan Gao.

Risks of specific congenital anomalies in offspring of women with diabetes: A systematic review and meta-analysis of population-based studies including over 80 million births

| **S3 Table. Ascertainment of maternal diabetes of the included studies in the systematic review and meta-analysis of population-based studies** | | |
| --- | --- | --- |
| **Author, year (Location)** | **Types of diabetes (n)** | **Exposure definition** |
| Arendt 2021 [1], Denmark, Sweden (European) | PGDM (2,518), T1D (2,020), T2D (498) | Women who had T1D if they were registered in the Danish National Patient Register with type 1 diabetes (ICD-8: 249; ICD-10: E10, O240) until two weeks post-partum, or women with two or more redeemed prescriptions for insulin (A10A) prior to delivery. Women who had T2D if they were registered in the Danish National Patient Register with this diagnosis (ICD-8: 250; ICD-10: E11, O24.1) until two weeks post-partum. |
| Bayoumi 2021 [2], Qatar (Asian-Pacifc) | PGDM (152), GDM (1,260) | GDM should be diagnosed at any time in pregnancy if one or more of the following criteria are met: fasting plasma glucose 5.1-6.9 mmol/l (92 -125 mg/dl), 1-hour plasma glucose ≥ 10.0 mmol/l (180 mg/dl) following a 75g oral glucose load, or 2-hour plasma glucose 8.5-11.0 mmol/l (153 -199 mg/dl) following a 75g oral glucose load. PGDM was defined by either T1D or T2D before the index pregnancy. |
| Schraw 2021 [3], United States (Americas) | PGDM (28,880), GDM (177,148) | Women were considered to have gestational diabetes if birth or fetal death certificates indicated they were diagnosed with GDM but not PGDM. |
| Allen 2020 [4], United Kingdom (European) | T1D (330) | Not reported |
| Dyck 2020 [5], Canada (Americas) | PGDM (3,306), GDM (10,289) | PGDM was defined as a pregnancy complicated by pre-existing diabetes mellitus. Women met the case definition for GDM if, within 150 days preceding or 90 days following a delivery date, they had one or more inpatient hospital separation records with a diagnostic code of ICD-9 250.x, 648.0 or 648.8 or ICD-10-CA E10-E14.xxx or O24.xxx, OR one or more physician service claims on different days with a diagnosis of ICD-9 250. |
| Lee 2020 [6], Canada (Americas) | PGDM (16,283), GDM (66,391) | Women who received a diagnosis of GDM but were not diagnosed with diabetes within 1 year postpartum were considered to have GDM alone. A woman was diagnosed with GDM if she had diagnostic codes from hospitalization records or ≥ 2 physician services claims. Women with PGDM were those who had a record in the Ontario Diabetes Database before the start of pregnancy, which was the baby’s birth date minus the gestational weeks. |
| Mowla 2020 [7], Finland (European) | PGDM (4,112), GDM (51,160) | PGDM, the primary exposure variable, were ascertained from MBR, and defined using ICD-10 codes O24.0–O24.3 including insulin dependent, non-insulin dependent, malnutrition-related, and unspecified type of diabetes prior to the index pregnancy. Maternal GDM using ICD-10 codes O24.4 and O24.9 relating to DM arising in pregnancy and diabetes in pregnancy that is unspecified. |
| Oliveira-Brancati 2020 [8], Brazil (Americas) | GDM (10) | Not reported |
| Raitio 2020 [9], Finland (European) | PGDM (20), GDM (93) | Maternal diabetes group contained both T1D and T2D diagnosed before conception. GDM group included all women with recorded diagnosis of gestational diabetes or abnormal oral glucose tolerance result. |
| Seghieri 2020 [10], Italy (European) | GDM (14,082) | The diagnosis of GDM was done by a validated algorithm according to the presence of at least one of following criteria, a): women with no previous anti-diabetic therapy who were prescribed insulin during pregnancy – the only antidiabetic drug currently used in pregnancy as from Italian guidelines – and stopped it after delivery, b): having received at least one specialistic visit by a diabetologist or having been included into an educational program for patients with diabetes before the index delivery c): having received an OGTT by six months after childbirth, d): having been discharged from hospital with ICD-9 code associated with GDM (648.8) in primary or secondary diagnosis. According to regional guidelines, the diagnosis of GDM, during the entire period considered, has currently been carried out following the IADPSG criteria after a single 75 g-2 h-OGTT, selectively targeting pregnant women with medium–high risk, according to Italian guidelines. |
| Tinker 2020 [11], United States (Americas) | PGDM (846), GDM (2,189) | PGDM was defined as reporting T1D or T2D diagnosed before the index pregnancy and GDM as having been diagnosed with GDM during the index pregnancy. Mothers who reported GDM in a previous pregnancy, those who reported diabetes diagnosed after the index pregnancy, or those with missing information on the type of diabetes or timing of diagnosis were excluded from the analysis. |
| Wu 2020 [12], United States (Americas) | PGDM (242,600), GDM (1,685,479) | Maternal PGDM was defined as having T1D or T2D diagnosed prior to the pregnancy, and maternal GDM was defined as having newly diagnosed diabetes during the pregnancy. |
| Chen 2019 [13], Canada (Americas) | PGDM (3,020), GDM (12,110) | In the ICD-9CM, PGDM was coded as ‘648.0’ or ‘250.*’, while GDM was coded as ‘648.8’. In the ICD-10, PGDM was coded as ‘O24.5’, ‘O24.6’, ‘O24.7’,‘E10’ and ‘E11’, while GDM was coded as ‘O24.8’. We did not make a distinction between different types of PGDM (type 1, type 2, other). During the study period (1996–2010), universal screening for GDM was in place in Quebec, following a two-step screening procedure. First, all pregnant women underwent a 50g 1hour oral glucose challenge test at 24–28 weeks of gestation. If blood glucose was ≥ 7.8 mmol/L, the 75g 2hours OGTT was then administered. GDM was diagnosed if two of three values equaled or exceeded the following cut-offs: fasting 5.3mmol/L, 1hour 10.0mmol/L, 2hours 8.6mmol/L. |
| Hildén 2019 [14], Sweden (European) | GDM (14,833) | Diagnosis of GDM was based on the 75-g OGTT. If elevated random, capillary blood glucose (> 9 mmol/l) was detected in the first trimester, an OGTT was performed with a repeat test during the second trimester. Otherwise the OGTT was performed in gestational week 28–32. During 1998–2012, there was a shift towards performing the OGTT in gestational week 24–28 which is in line with international guidelines.The Swedish MBR does not have the timing of OGTT documented. During the study period, the main diagnostic criteria for GDM were fasting, capillary, wholeblood glucose ≥ 6 mmol/l and/or 2-h blood glucose ≥ 9 mmol/l. In the middle of the study period, there was a switch from measuring whole-blood glucose to plasma glucose. The diagnostic criteria based on plasma glucose included cut-off levels for fasting glucose of ≥ 7.0 mmol/l and for 2-h glucose of ≥ 10.0 mmol/l. A small region in Sweden has offered a simplified 75-g OGTT (including only the 2-h blood glucose measurement) to all pregnant women since 1995. The diagnostic criteria for GDM for this simplified OGTT was 2-h blood glucose ≥ 10.0 mmol/l. In another region, during 1998–2010, only women with values corresponding to overt diabetes (fasting capillary plasma glucose ≥ 7 mmol/l or 2-h plasma glucose ≥ 12.2 mmol/l) were diagnosed with GDM. This region represents 20–25% of the pregnant population. GDM was identified as ICD code O24.4A or O24.4B. |
| Klungsøyr 2019 [15], Norway (European) | PGDM (11,698) | PGDM: type 1, 2 and unspecified. |
| Liu 2019 [16], Canada (Americas) | PGDM (23,300), T1D (9,257), T2D (14,043) | Not reported |
| Wei 2019 [17], China (Asian-Pacifc) | PGDM (76,297) | Not reported |
| Yang 2019 [18], United States (Americas) | PGDM (4,134), GDM (32,605) | PGDM was defined as having glucose intolerance requiring treatment before pregnancy diagnosed by a physician. GDM was defined as having glucose intolerance, diagnosed during this pregnancy by a physician. |
| Ludvigsson 2018 [19], Sweden (European) | T1D (2,458) | T1D , T2D, other diabetes, GDM (ICD-10: E10-E14, O24); Diabetes during pregnancy (includes T1D , T2D, other diabetes, GDM) (ICD-10: O24). |
| Arendt 2018 [20], Denmark, Sweden (European) | PGDM (13,612), GDM (21,321) | PGDM was defined as diagnosis of either T1D or T2D before birth. In Danish data, we had information on diagnoses recorded from 1978 to 2012. Using this, boys of mothers diagnosed with the unspecific diabetes mellitus code (ICD-8: 250, Denmark;1978–1986) before birth were reclassified as exposed to either T1D or T2D, depending on whether the mothers were diagnosed with a specific code for T1D or T2D later in time. Since we only had information on diagnoses until birth in the Swedish data, we could not reclassify individuals with an unspecific diabetes mellitus code based on later diagnose. GDM has been coded independently throughout the study period (ICD-8 (Denmark;1978–1993): 63474, Y6449, ICD-9 (Sweden;1986–1996): 648W and ICD-10 (Denmark;1994–2012 and Sweden;1997– 2012): O244, O249). |
| Kovalenko 2018 [21], Russia (Asian-Pacifc) | PGDM (98) | Not reported |
| Soliman 2018 [22], Qatar (Asian-Pacifc) | PGDM (233), GDM (3,018) | According to the criteria of the International Association of the Diabetes in Pregnancy Study Group (IADPSG). |
| Billionnet 2017 [23], France (European) | PGDM (3,198), T1D (1,291), T2D (1,907), GDM (57,629) | T1D: Insulin dispensed at least 3 times in the year before pregnancy and insulin dispensed at least once from 6 months to 1 year after delivery were included.  T2D: Oral glucose-lowering agents or insulin dispensed at least 3 times in the year before pregnancy and at least one of the following criteria: - an HbA1c assay performed or glucose strips dispensed in the year before pregnancy - long-term disease status for diabetes before pregnancy - oral glucose-lowering agents or insulin dispensed at least once during pregnancy or in the year after delivery GDM was classified as insulin-treated when insulin was dispensed at least once during pregnancy. At least one of the following criteria: - insulin dispensed at least once during pregnancy - at least 200 glucose strips dispensed during pregnancy on at least 2 different occasions - a diagnosis of diabetes recorded during the delivery admission (ICD-10 codes E10–E14, O240–O244, O249). |
| Hoang 2017 [24], United States (Americas) | PGDM (12,116), GDM (75,030) | Not reported |
| Darke 2016 [25], United Kingdom (European) | PGDM (54) | Not reported |
| Lai 2016 [26], Canada (Americas) | PGDM (2,536), GDM (18,554) | Woman was dentified as having PGDM if the diabetes was diagnosed prior to conception. Universal screening of GDM is recommended in Alberta for pregnant women between 24 and 28 weeks of gestation using a 50 g oral glucose challenge test. If the 1-hour plasma glucose value is 10.3 mmol/L and above, GDM is diagnosed. If the 1hPG value is between 7.8 and 10.2 mmol/L, a 2-hour 75 g OGTT is conducted, and GDM is diagnosed if two of these plasma glucose values are met (fasting ≥ 5.3 mmol/L, 1h ≥ 10.6 mmol/L, 2h ≥ 8.9 mmol/L). A case of GDM was identified if the woman had at least one hospital admission (including ambulatory care visit) or two outpatient physician visits for GDM (ICD-9 648.8, ICD-10 O24.4, O24.8) during the pregnancy time and up to 180 days post delivery or the conception date of the next pregnancy whichever came earlier. The algorithm also included a diagnosis code for diabetes (ICD-9 250, ICD-10 E10-E14) during the gestation period, as sometimes women with GDM are simply coded as having diabetes. Once a woman had PGDM, all her subsequent pregnancies would be classified as PGDM. However, if a woman had GDM, she was not assumed to have GDM for her future pregnancies unless the criteria for a diagnosis of GDM described above were met. |
| Leirgul 2016 [27], Norway (European) | PGDM (5,618), GDM (9,726) | The criteria for GDM were extended to include impaired glucose tolerance with plasma glucose 7.8 mmol/L or greater after a glucose load test or fasting glucose level 7.0 mmol/L or greater. Maternal diabetes has been notified in the maternal health text field using the ICD codes for T1D (ICD-10 codes E10.0–E10.9, O24.0; ICD-9 code 250.1), T2D [ICD-10: E11.0–E11.9, O24.1; ICD-9: 250.2], GDM (ICD-10: O24.4, O24.9; ICD-9: 648.8), and unspecified diabetes (ICD-10: E13.0–E13.9, E14.0–E14.9, O24.3; ICD-9: 250.0, 250.3–250.9). Any antidiabetic medication during pregnancy was registered by a yes–no variable. |
| Øyen 2016 [28], Denmark (European) | PGDM (7,926), GDM (6,716) | PGDM was defined as pregestational registration of ICD-8 codes 250 or 249, or ICD-10 codes E10.0 to E11.9. Women who developed GDM (ICD-8 code 634.74; ICD-10 code O24.4) were identifed in the second trimester (13–27 gestational weeks) or third trimester (≥ 28 gestational weeks). |
| Tain 2016 [29], China (Asian-Pacifc) | PGDM (10), GDM (35) | Not reported |
| Dart 2015 [30], Canada (Americas) | PGDM (150), GDM (197) | PGDM was identified using a modified National Diabetes Surveillance System definition: 2 or more outpatient or 1 or more inpatient ICD codes for DM over the 2-year period prior to index pregnancy and including the first 20 weeks of the index or previous pregnancy within this time frame (as not physiologically GDM). An ICD code for DM coded at 21 or more weeks’ gestational age was considered GDM for the index pregnancy. GDM in previous pregnancies was not considered an exposure for the current pregnancy. |
| Liu 2015 [31], Canada (Americas) | PGDM (531,020), T1D (266,078), T2D (264,942) | Not reported |
| Liu 2015 [32], China (Asian-Pacifc) | GDM (3,060) | Not reported |
| Mavrogenis 2015 [33], Hungary (European) | GDM (183) | Not reported |
| Csaky-Szunyogh 2014 [34], Hungary (European) | GDM (230) | Not reported |
| Feig 2014 [35], Canada (Americas) | PGDM (13,278), GDM (45,384) | Women with a diagnosis of diabetes in the 280 days prior to the index date were defined as having GDM based on their hospitalization records and outpatient data from physician services claims. Women with PGDM were those who were diagnosed with diabetes. |
| Persson 2014 [36], Sweden (European) | PGDM (4,504), GDM (8,602) | Women with T1D, T2D or GDM were identified based on the codes of the ICD-10 codes. All pregnant women are tested for gestational diabetes either by random capillary plasma glucose performed 4–6 times during pregnancy, or by a simplified 75-g oral glucose tolerance test. During the study period, women were selected for an OGTT based on random capillary plasma glucose tests ≥ 9 mmol/l, or if traditional risk factors are present. In the southern part of Sweden, all pregnant women have been offered a simplified version of the OGTT (omitting fasting blood glucose) since 1995 as a one-step screening and diagnostic test.The main diagnostic criteria for GDM applied in Sweden are based on the Diabetes Pregnancy Study Group recommendation from 1991; i.e. a fasting capillary whole blood glucose ≥ 6.1 mmol/l (fasting plasma glucose ≥ 7.0 mmol/l) and/or 2-h capillary whole blood glucose ≥ 9 mmol/l (plasma glucose ≥ 10 mmol/l) after a 75-g OGTT. |
| Vereczkey 2014 [37], Hungary (European) | PGDM (329) | Not reported |
| Vinceti 2014 [38], Italy (European) | PGDM (2,269) | All hospital discharge records in which a diagnostic code related to PGDM was present, by using the diagnostic codes of the ICD IX Edition no. 250.0 and 648.0. |
| Liu 2013 [39], Canada (Americas) | PGDM (1,367,302) | PGDM (T1D: E10, O245, O240; T2D: E11, O241, O246). |
| Parker 2013 [40], United States, Canada (Americas) | PGDM (50), GDM (410) | PGDM was defined as the onset of T1D or T2D before pregnancy. GDM was defined as the onset of DM after the first lunar month of the index pregnancy. |
| Bell 2012 [41], United Kingdom (European) | PGDM (1,677) | The Northern Diabetes in Pregnancy Survey (NorDIP) records details of all known pregnancies, irrespective of outcome, in women resident in the study area and diagnosed with diabetes at least 6 months prior to conception. Pregnancies in women with GDM (i.e. hyperglycaemia first diagnosed during pregnancy) are not included. |
| Garne 2012 [42], Norway, Denmark, Germany, Netherlands, Belgium, Wales, United Kingdom, Ireland, Switzerland, France, Italy, Spain, Portugal, Malta (European) | PGDM (699) | Not reported |
| Wu 2012 [43], Denmark (European) | PGDM (14,144), T1D (1,743), T2D (12,401), GDM (11,507) | All patients with T1D or T2D were recoded with the same code (ICD-8: 250) between 1977 and 1986 in the Danish version of ICD-8. From 1987 to 1993, the Danish version of ICD-8 differentiated between T1D (ICD-8: 249) and T2D (ICD-8:250). From 1994 to 2008, the ICD-10 differentiated between T1D (ICD-10: E10, O240), T2D (ICD-10: E11, O241), GDM(ICD-10: O24.4, O24.9), and unspecified diabetes (ICD-10: E12, E13, E14, O242, and O243). |
| Alverson 2011 [44], United States (Americas) | GDM (229) | Not reported |
| Bánhidy 2010 [45], Hungary (European) | PGDM (385), T1D (167), T2D (218), GDM (349) | The diagnosis of T1D was accepted on the basis of either its specified diagnosis (all pregnant women were treated with insulin) or unspecified DM with insulin treatment. The diagnosis of T2D was accepted on the basis of either its specified diagnosis or unspecified DM diagnosed before the conception of the study pregnancy without insulin treatment. The diagnosis of GDM was based on the recognition of DM during the study pregnancy. |
| Eidem 2010 [46], Norway (European) | T1D (1,583) | Not reported |
| Fadl 2010 [47], Sweden (European) | GDM (10,525) | Diagnosis of GDM ICD-9 and ICD-10 was based on the 75 g OGTT. During the study period, the main diagnostic criteria for GDM were fasting capillary whole blood glucose ≥ 6.1 mmol/l and /or 2 h blood glucose ≥ 9.0 mmol/l according to the Diabetic Pregnancy Study Group. In one small area, impaired glucose tolerance (IGT, fasting blood glucose < 6.1 mmol/l and 2 h blood glucose 9.0–11.1 mmol/l) was not included in GDM diagnoses during the last years of the study. |
| Peticca 2009 [48], Canada (Americas) | PGDM (1,420), T1D (904), T2D (516), GDM (3,188) | Not reported |
| Correa 2008 [49], United States (Americas) | PGDM (190), GDM (710) | PGDM was defined if the mother reported having been diagnosed with T1D or T2D before the birth of the index infant. GDM was defined if the mother reported having been diagnosed with GDM during the index pregnancy. |
| Macintosh 2006 [50], England, Wales, Northern Ireland (European) | PGDM (2,359) | PGDM was defined as either T1D or T2D that had been diagnosed at least one year before the woman’s estimated delivery date. We excluded the group, usually with T2D, who present during pregnancy but in whom the diagnosis cannot be confirmed until after pregnancy. |
| Yang 2006 [51], Canada (Americas) | PGDM (516) | Not reported |
| Anderson 2005 [52], United States (Americas) | PGDM (22), GDM (61) | Not reported |
| Nielsen 2005 [53], Hungary (European) | PGDM (113) | Not reported |
| Sharpe 2005 [54], Australia (Asian-Pacifc) | PGDM (946) | In 1999, increased knowledge of the predictive value of blood glucose levels led to the lowering of the fasting plasma glucose “cutoff” for diagnosing PGDM from ≥ 7.8 to ≥ 7.0 mmol/lite. In addition, the method of recording PGDM on the SBR changed in 1991. |
| Loffredo 2001 [55], Columbia (Asian-Pacifc) | PGDM (59) | Not reported |
| Croen 2000 [56], United States (Americas) | GDM (23) | Not reported |
| Von Kries 1997 [57], Germany (European) | PGDM (2,352) | Not reported |
| Janssen 1996 [58], United States (Americas) | PGDM (1,511), GDM (8,869) | Not reported |
| Becerra 1990 [59], United States (Americas) | T1D (28) | Not reported |
| Abbreviations: DM, diabetes mellitus; GDM, Gestational diabetes mellitus; ICD-8, International Classification of Diseases, Eighth Revision; ICD-9, International Classification of Diseases, Ninth Revision; ICD-9-CM, International Classification of Diseases, Ninth Revision, Clinical Modification; ICD-10, International Classification of Diseases, Tenth Revision; ICD-10-CA, International Classification of Diseases, Tenth Revision, in Canada; ICD-10-CM, International Classification of Diseases, Tenth Revision, Clinical Modification; OGTT, Oral glucose tolerance test; PGDM, Pre-gestational diabetes mellitus; T1D, Type 1 diabetes; T2D, Type 2 diabetes. | | |

References

1.Arendt LH, Pedersen LH, Pedersen L, Ovesen PG, Henriksen TB, Lindhard MS, et al. Glycemic Control in Pregnancies Complicated by Pre-Existing Diabetes Mellitus and Congenital Malformations: A Danish Population-Based Study. Clin Epidemiol. 2021;13:615–26.

2.Bayoumi MAA, Masri RM, Matani NYS, Hendaus MA, Masri MM, Chandra P, et al. Maternal and neonatal outcomes in mothers with diabetes mellitus in qatari population.BMC Pregnancy Childbirth. 2021;21(1):651

3.Schraw JM, Langlois PH, Lupo PJ. Comprehensive assessment of the associations between maternal diabetes and structural birth defects in offspring: a phenome-wide association study. Ann Epidemiol. 2021;53:14–20.e8.

4.Allen LA, Cannings-John RL, Evans A, Thayer DS, French R, Paranjothy S, et al. Pregnancy in teenagers diagnosed with type 1 diabetes mellitus in childhood: a national population-based e-cohort study. Diabetologia. 2020;63:799–810.

5.Dyck RF, Karunanayake C, Pahwa P, Stang M, Erickson RL, Osgood ND. Congenital Anomalies of the Kidney and Urinary Tract (CAKUT): An Emerging Relationship With Pregestational Diabetes Mellitus Among First Nations and Non-First Nations People in Saskatchewan-Results From the DIP: ORRIIGENSS Project. Can J Diabetes. 2020:S1499–2671.

6.Lee D, Booth GL, Ray JG, Ling V, Feig DS. Undiagnosed type 2 diabetes during pregnancy is associated with increased perinatal mortality: a large population-based cohort study in Ontario, Canada. Diabet Med. 2020;37:1696–1704.

7.Mowla S, Gissler M, Räisänen S, Kancherla V. Association between maternal pregestational diabetes mellitus and spina bifida: A population-based case-control study, Finland, 2000-2014. Birth Defects Res. 2020;112:186–95.

8.Oliveira-Brancati CIF, Ferrarese VCC, Costa AR, Fett-Conte AC. Birth defects in Brazil: Outcomes of a population-based study. Genet Mol Biol. 2020;43:e20180186.

9.Raitio A, Tauriainen A, Leinonen MK, Syvänen J, Kemppainen T, Löyttyniemi E, et al. Maternal risk factors for gastroschisis: A population-based case-control study. Birth Defects Res. 2020;112:989–95.

10.Seghieri G, Di Cianni G, Seghieri M, Lacaria E, Corsi E, Lencioni C, et al. Risk and adverse outcomes of gestational diabetes in migrants: A population cohort study. Diabetes Res Clin Pract. 2020;163:108128.

11.Tinker SC, Gilboa SM, Moore CA, Waller DK, Simeone RM, Kim SY, et al. Specific birth defects in pregnancies of women with diabetes: National Birth Defects Prevention Study, 1997–2011. Am J Obstet Gynecol. 2020;222:176.e1–11.

12.Wu Y, Liu B, Sun Y, Du Y, Santillan MK, Santillan DA, et al. Association of Maternal Prepregnancy Diabetes and Gestational Diabetes Mellitus With Congenital Anomalies of the Newborn. Diabetes Care. 2020;43:2983–90.

13.Chen L, Wang WJ, Auger N, Xiao L, Torrie J, McHugh NG, et al. Diabetes in pregnancy in associations with perinatal and postneonatal mortality in First Nations and non-Indigenous populations in Quebec, Canada: population-based linked birth cohort study. BMJ Open. 2019;9:e025084.

14.Hildén K, Hanson U, Persson M, Magnuson A, Simmons D, Fadl H. Gestational diabetes and adiposity are independent risk factors for perinatal outcomes: a population based cohort study in Sweden. Diabet Med. 2019;36:151–57.

15.Klungsøyr K, Nordtveit TI, Kaastad TS, Solberg S, Sletten IN, Vik AK. Epidemiology of limb reduction defects as registered in the Medical Birth Registry of Norway, 1970-2016: Population based study. PLoS One. 2019;14(7):e0219930.

16.Liu S, Evans J, MacFarlane AJ, Ananth CV, Little J, Kramer MS, et al. Association of maternal risk factors with the recent rise of neural tube defects in Canada. Paediatr Perinat Epidemiol. 2019;33:145–53.

17.Wei Y, Xu Q, Yang H, Yang Y, Wang L, Chen H, et al. Preconception diabetes mellitus and adverse pregnancy outcomes in over 6.4 million women: A population-based cohort study in China. PLoS Med. 2019;16:e1002926.

18.Yang G-R, Dye TD, Li D. Effects of pre-gestational diabetes mellitus and gestational diabetes mellitus on macrosomia and birth defects in Upstate New York. Diabetes Res Clin Pract. 2019;155:107811.

19.Ludvigsson JF, Neovius M, Söderling J, Gudbjörnsdottir S, Svensson AM, Franzén S, et al. Periconception glycaemic control in women with type 1 diabetes and risk of major birth defects: population based cohort study in Sweden. BMJ. 2018;362:k2638.

20.Arendt LH, Lindhard MS, Henriksen TB, Olsen J, Cnattingius S, Petersson G, et al. Maternal Diabetes Mellitus and Genital Anomalies in Male Offspring: A Nationwide Cohort Study in 2 Nordic Countries. Epidemiology. 2018;29:280–89.

21.Kovalenko AA, Anda EE, Odland JØ, Nieboer E, Brenn T, Krettek A. Risk Factors for Ventricular Septal Defects in Murmansk County, Russia: A Registry-Based Study. Int J Env Res Public Health. 2018;15:1320.

22.Soliman A, Salama H, Al Rifai H, De Sanctis V, Al-Obaidly S, Al Qubasi M, et al. The effect of different forms of dysglycemia during pregnancy on maternal and fetal outcomes in treated women and comparison with large cohort studies. Acta Biomed. 2018;89:11–21.

23.Billionnet C, Mitanchez D, Weill A, Nizard J, Alla F, Hartemann A, et al. Gestational diabetes and adverse perinatal outcomes from 716,152 births in France in 2012. Diabetologia. 2017;60:636–44.

24.Hoang TT, Marengo LK, Mitchell LE, Canfield MA, Agopian AJ. Original Findings and Updated Meta-Analysis for the Association Between Maternal Diabetes and Risk for Congenital Heart Disease Phenotypes. Am J Epidemiol. 2017;186:118–28.

25.Darke J, Glinianaia SV, Marsden P, Bell R. Pregestational diabetes is associated with adverse outcomes in twin pregnancies: a regional register-based study. Acta Obstet Gynecol Scand. 2016;95:339–46.

26.Lai FY, Johnson JA, Dover D, Kaul P. Outcomes of singleton and twin pregnancies complicated by pre-existing diabetes and gestational diabetes: A population-based study in Alberta, Canada, 2005–11. J Diabetes. 2016;8:45–55.

27.Leirgul E, Brodwall K, Greve G, Vollset SE, Holmstrøm H, Tell GS, et al. Maternal Diabetes, Birth Weight, and Neonatal Risk of Congenital Heart Defects in Norway, 1994–2009. Obstet Gynecol. 2016;128:1116–25.

28.Øyen N, Diaz LJ, Leirgul E, Boyd HA, Priest J, Mathiesen ER, et al. Prepregnancy Diabetes and Offspring Risk of Congenital Heart Disease: A Nationwide Cohort Study. Circulation. 2016;133:2243–53.

29.Tain Y-L, Luh H, Lin C-Y, Hsu C-N. Incidence and Risks of Congenital Anomalies of Kidney and Urinary Tract in Newborns: A Population-Based Case-Control Study in Taiwan. Medicine. 2016;95:e2659.

30.Dart AB, Ruth CA, Sellers EA, Au W, Dean HJ. Maternal diabetes mellitus and congenital anomalies of the kidney and urinary tract (CAKUT) in the child. Am J Kidney Dis. 2015;65:684–91.

31.Liu S, Rouleau J, León JA, Sauve R, Joseph KS, Ray JG. Impact of pre-pregnancy diabetes mellitus on congenital anomalies, Canada, 2002–2012. Health Promot Chronic Dis Prev Can. 2015;35:79–84.

32.Liu X, Liu G, Wang P, Huang Y, Liu E, Li D, et al. Prevalence of congenital heart disease and its related risk indicators among 90,796 Chinese infants aged less than 6 months in Tianjin. Int J Epidemiol. 2015;44:884–93.

33.Mavrogenis S, Urban R, Czeizel AE. Pregnancy complications in the mothers who delivered boys with isolated hypospadias - a population-based case-control study. J Matern Fetal Neona. 2015;28:489–93.

34.Csáky-Szunyogh M, Vereczkey A, Kósa Z, Gerencsér B, Czeizel AE. Risk factors in the origin of congenital left-ventricular outflow-tract obstruction defects of the heart: a population-based case-control study. Pediatr Cardiol. 2014;35:108–20.

35.Feig DS, Hwee J, Shah BR, Booth GL, Bierman AS, Lipscombe LL. Trends in incidence of diabetes in pregnancy and serious perinatal outcomes: a large, population-based study in Ontario, Canada, 1996–2010. Diabetes Care. 2014;37:1590–96.

36.Persson M, Fadl H. Perinatal outcome in relation to fetal sex in offspring to mothers with pre-gestational and gestational diabetes--a population-based study. Diabet Med. 2014;31:1047–54.

37.Vereczkey A, Gerencsér B, Czeizel AE, Szabó I. Association of certain chronic maternal diseases with the risk of specific congenital heart defects: a population-based study. Eur J Obstet Gynecol Reprod Biol. 2014;182:1–6.

38.Vinceti M, Malagoli C, Rothman KJ, Rodolfi R, Astolfi G, Calzolari E, et al. Risk of birth defects associated with maternal pregestational diabetes. Eur J Epidemiol. 2014; 29:411–18.

39.Liu S, Joseph KS, Lisonkova S, Rouleau J, Van den Hof M, Sauve R, et al. Association between maternal chronic conditions and congenital heart defects: a population-based cohort study. Circulation. 2013;128:583–89.

40.Parker SE, Yazdy MM, Tinker SC, Mitchell AA, Werler MM. The impact of folic acid intake on the association among diabetes mellitus, obesity, and spina bifida. Am J Obstet Gynecol. 2013;209:239.e231–38.

41.Bell R, Glinianaia SV, Tennant PWG, Bilous RW, Rankin J. Peri-conception hyperglycaemia and nephropathy are associated with risk of congenital anomaly in women with pre-existing diabetes: a population-based cohort study. Diabetologia. 2012;55:936–47.

42.Garne E, Loane M, Dolk H, Barisic I, Addor MC, Arriola L, et al. Spectrum of congenital anomalies in pregnancies with pregestational diabetes. Birth Defects Res A Clin Mol Teratol. 2012;94:134–40.

43.Wu CS, Nohr EA, Bech BH, Vestergaard M, Olsen J. Long-term health outcomes in children born to mothers with diabetes: a population-based cohort study. PLoS One. 2012;7:e36727.

44.Alverson CJ, Strickland MJ, Gilboa SM, Correa A. Maternal smoking and congenital heart defects in the Baltimore-Washington Infant Study. Pediatrics. 2011;127:e647–53.

45.Bánhidy F, Acs N, Puhó EH, Czeizel AE. Congenital abnormalities in the offspring of pregnant women with type 1, type 2 and gestational diabetes mellitus: a population-based case-control study. Congenit Anom (Kyoto). 2010;50:115–21.

46.Eidem I, Stene LC, Henriksen T, Hanssen KF, Vangen S, Vollset SE, et al. Congenital anomalies in newborns of women with type 1 diabetes: nationwide population-based study in Norway, 1999-2004. Acta Obstet Gynecol Scand. 2010;89:1403–11.

47.Fadl HE, Ostlund IKM, Magnuson AFK, Hanson USB. Maternal and neonatal outcomes and time trends of gestational diabetes mellitus in Sweden from 1991 to 2003. Diabet Med. 2010;27:436–41.

48.Peticca P, Keely EJ, Walker MC, Yang Q, Bottomley J. Pregnancy outcomes in diabetes subtypes: how do they compare? A province-based study of Ontario, 2005-2006. J Obstet Gynaecol Can. 2009;31:487–96.

49.Correa A, Gilboa SM, Besser LM, Botto LD, Moore CA, Hobbs CA, et al. Diabetes mellitus and birth defects. Am J Obstet Gynecol. 2008;199:237.e1–9.

50.Macintosh MC, Fleming KM, Bailey JA, Doyle P, Modder J, Acolet D, et al. Perinatal mortality and congenital anomalies in babies of women with type 1 or type 2 diabetes in England, Wales, and Northern Ireland: population based study. BMJ. 2006;333:177.

51.Yang J, Cummings EA, O'Connell C, Jangaard K. Fetal and neonatal outcomes of diabetic pregnancies. Obstet Gynecol. 2006;108:644–50.

52.Anderson JL, Waller DK, Canfield MA, Shaw GM, Watkins ML, Werler MM. Maternal obesity, gestational diabetes, and central nervous system birth defects. Epidemiology. 2005;16:87–92.

53.Nielsen GL, Nørgard B, Puho E, Rothman KJ, Sørensen HT, Czeizel AE. Risk of specific congenital abnormalities in offspring of women with diabetes. Diabet Med. 2005;22:693–96.

54.Sharpe PB, Chan A, Haan EA, Hiller JE. Maternal diabetes and congenital anomalies in South Australia 1986-2000: a population-based cohort study. Birth Defects Res A Clin Mol Teratol. 2005;73:605–11.

55.Loffredo CA, Wilson PD, Ferencz C. Maternal diabetes: an independent risk factor for major cardiovascular malformations with increased mortality of affected infants. Teratology. 2001;64:98–106.

56.Croen LA, Shaw GM, Lammer EJ. Risk factors for cytogenetically normal holoprosencephaly in California: a population-based case-control study. Am J Med Genet. 2000;90:320–25.

57.von Kries R, Kimmerle R, Schmidt JE, Hachmeister A, Böhm O, Wolf HG. Pregnancy outcomes in mothers with pregestational diabetes: a population-based study in North Rhine (Germany) from 1988 to 1993. Eur J Pediatr. 1997;156:963–67.

58.Janssen PA, Rothman I, Schwartz SM. Congenital malformations in newborns of women with established and gestational diabetes in Washington State, 1984-91. Paediatr Perinat Epidemiol. 1996;10:52–63.

59.Becerra JE, Khoury MJ, Cordero JF, Erickson JD. Diabetes mellitus during pregnancy and the risks for specific birth defects: a population-based case-control study. Pediatrics. 1990;85:1–9.
