# Supplementary material for: Risks of specific congenital anomalies in offspring of women with diabetes: A systematic review and meta-analysis of population-based studies including over 80 million births
Source: PLoS Med. 2022 Feb 1;19(2):e1003900. doi: 10.1371/journal.pmed.1003900 (PMC8806075; doi:10.1371/journal.pmed.1003900)
Supplement: S4 Table — (DOCX) [file pmed.1003900.s006.docx]

**S4 Table**

This supporting information formed part of the original submission and has been peer reviewed.

We post it as supplied by the authors.

Supplement to: Tie-Ning Zhang, Xin-Mei Huang, Xin-Yi Zhao, Wei Wang, Ri Wen, Shan-Yan Gao.

Risks of specific congenital anomalies in offspring of women with diabetes: A systematic review and meta-analysis of population-based studies including over 80 million births

| **S4 Table. Characteristics of population-based studies of maternal diabetes and congenital anomalies** | | | | | | | |
| --- | --- | --- | --- | --- | --- | --- | --- |
| **Author, year (Location)** | **Study period** | **Data sources** | **Types of diabetes** | **Outcomes (No of events),**  **No of participants** | **Outcome definition** | **Types of birth** | **Adjusted factors** |
| Arendt 2021 [1], Denmark, Sweden (European) | 2000-2015 | Danish health registers and  the LABKA database | PGDM (T1D, T2D) | Major congenital maformations (8,292), Nervous system (416), Eye (380), Orofacial clefts (529), Congenital heart diseases (3,164), Digestive system (548), Genital organs (888), Urinary system (958), Musculoskeletal system (1,016), 314,245 | ICD-10 codes | Livebirths | Maternal age, cigarette smoking, parity, and year of birth. |
| Bayoumi 2021 [2], Qatar (Asian-Pacifc) | 2017 | The Neonatal Intensive Care Unit (NICU) of Women’s Wellness and Research Center (WWRC) | PGDM, GDM | Major congenital malformations (41), 5,195 | Not reported | Livebirths | None |
| Schraw 2021 [3], United States (Americas) | 1999–2015 | The Texas Birth Defects Registry (TBDR) | PGDM, GDM | Any birth defect (521,364), Nervous system (31,583), Encephalocele (514), Hydrocephaly (7,786), Eye, ear, face, and neck (35,036), Cardiovascular (225,979), Transposition of great vessels (3,318), Tetralogy of Fallot (2,441), Single ventricle (566), VSD (37,907), ASD (76,769), AVSD (2,761), Coarctation of aorta (3,313), Pulmonary artery anomalies (16,255), Cleft lip (6,702), Alimentary & digestive (6,078), Genitourinary (102,690), Renal a/dysgenesis (3,695), Musculoskeletal (111,712), polydactyly (13,447), Upper limb reduction defects (2,546), Other lower limb anomalies (12,046), Multiple congenital anomalies, 6,543,385 | ICD BPA codes | Livebirths | Maternal age, race/ethnicity, education, and number of previous livebirths. |
| Allen 2020 [4], United Kingdom (European) | 1995–2013 | The Brecon Register, the SAIL Databank, the Wales Electronic Cohort for Children (WECC), the Congenital Anomaly Register and Information Service (CARIS), the Patient Episode Database for Wales (PEDW) | T1D | Congenital malformation (8,509), 197,796 | EUROCAT | Live or stillbirths beyond 24 weeks’ gestation | Maternal age, maternal deprivation quintile, parity, maternal smoking, and sex of baby. |
| Dyck 2020 [5], Canada (Americas) | 1980–2009 | ORRIIGENSS project (Diabetes In Pregnancy: Outcomes and Ramifications from a Retrospective longitudinal Intra- Inter-GENerational Study in Saskatchewan) | PGDM, GDM | CAKUT (2,540), 411,055 | ICD-9, ICD-10-CA codes | Live births, stillbirths | Ethnicity. |
| Lee 2020 [6], Canada (Americas) | 2002–2015 | The Canadian Institute for Health Information Discharge Abstract Database,the Mother-Baby database,the Ontario Diabetes Database, the Better Outcomes Registry and Network Database | PGDM, GDM | Congenital anomalies (3,110), 995,990 | ICD-9, ICD-10 codes | Not reported | Age, income, having a primary care physician, previous parity, ethnicity, and recent immigration. |
| Mowla 2020 [7], Finland (European) | 2000–2014 | Finland's Register of Congenital Malformations (RCM), the Finnish Hospital Discharge Register (FHDR), the Finnish Medical Birth Register (MBR), Census data from Statistics Finland | PGDM, GDM | Spina bifida (181), 876,853 | EUROCAT | Live births, stillbirths | Age at delivery, education, income level, BMI. |
| Oliveira-Brancati 2020 [8], Brazil (Americas) | 2011–2012 | Not reported | GDM | Birth defects (167), 336 | Not reported | Live births, stillbirths | Control Group: Matched for gender and born in the same hospital immediately after each study case. |
| Raitio 2020 [9], Finland (European) | 2004–2014 | The Finnish Register of Congenital Malformations, the Medical Birth Register, the Register on the Induced Abortions, the Care Register for Health Care | PGDM, GDM | Gastroschisis (154), 1,064 | ICD codes | Live births, stillbirths, elective terminations of pregnancy | Control Group: Matched for maternal age (± 1 year), residency, and time of conception (± 1 month). |
| Seghieri 2020 [10], Italy (European) | 2012–2017 | The Regional Health System (RHS) | GDM | Malformations (357), 122,652 | Not reported | Live births | Age, parity, calendar year of delivery, gestational week at delivery, pre-gestational overweight/obesity, education, employment and smoking. |
| Tinker 2020 [11], United States (Americas) | 1997–2011 | The National Birth Defects Prevention Study (NBDPS) | PGDM, GDM | Anencephaly (627), Spina bifida (1,236), Encephalocele (221), Holoprosencephaly (163), Hydrocephaly (505), Cleft palate alone (1,570), Cleft lip with or without cleft palate (3,046), Hypospadias (2,542), Renal agenesis /hypoplasia (180), Longitudinal limb deficiency (469), Transverse limb deficiency (703), Diaphragmatic hernia (851), Omphalocele (431), Gastroschisis (1,429), Heterotaxy with cardiac defects (343), Truncus arteriosus (134), Tetralogy of Fallot (1,181), D-transposition of the great arteries (753), Atrioventricular septal defect (359), Total APVR (291), Partial APVR (77), Hypoplastic left heart syndrome (640), Coarctation of the aorta (1,149), Pulmonary atresia (255), PVS (1,533), Perimembranous VSD (1,651), ASD (3,009), Single ventricle complex (313), VSD and ASD (747), 42,454 | Not reported | Live births, fetal deaths, and terminations | Maternal BMI, age, education, race/ethnicity, and study site. |
| Wu 2020 [12], United States (Americas) | 2011–2018 | National Vital Statistics System (NVSS) | PGDM, GDM | Congenital anomalies (90,061), Cyanotic congenital heart disease (20,175), Hypospadias (16,168), Cleft lip with or without cleft palate (17,468), Gastroschisis (7,430), Cleft palate alone (6,848), Meningomyelocele/spina bifida (4,306), Congenital diaphragmatic hernia (3,869), Limb reduction defect (3,850), Anencephaly (2,993), Omphalocele (2,977), 29,211,974 | Not reported | Live births | Maternal age, race/ethnicity, education levels, marital status, parity, smoking before pregnancy, smoking during pregnancy, timing of initiation of prenatal care, prepregnancy BMI, infant sex, prepregnancy hypertension. |
| Chen 2019 [13], Canada (Americas) | 1996–2010 | Birth and infant death database of the Institut de la Statistique du Québec (ISQ) | PGDM, GDM | Birth defects, 234,850 | ICD-9 CM, ICD-10 codes | Not reported | Cluster effects (multiple infants of the same mother), maternal age, education, marital status, parity and rural residence, chronic hypertension, heart disease, renal disease, genitourinary infection, anaemia and gestational hypertension and pre-eclampsia. |
| Hildén 2019 [14], Sweden (European) | 1998–2012 | The Medical Birth Register (MBR) | GDM | Malformations (50,549), 1,455,667 | ICD-10 codes | Not reported | Maternal age, BMI, non-Nordic origin, parity, smoking and chronic hypertension. |
| Klungsøyr 2019 [15], Norway (European) | 1970–2016 | The Medical Birth Registry of Norway (MBRN) | PGDM | Limb reduction defects (1,369), 2,740,900 | ICD-10 codes | Live births | Maternal age, smoking, and folate/multivitamin supplements. |
| Liu 2019 [16], Canada (Americas) | 2004–2015 | The Discharge Abstract Database (DAD) | PGDM (T1D, T2D) | Spina bifida (858), 3,439,330 | ICD-10 CM codes | Live births, stillbirths (including late pregnancy terminations) | Infant's birth year, problematic substance use, chronic illness, epilepsy, obesity, multiple pregnancy, and rural residence. |
| Wei 2019 [17], China (Asian-Pacifc) | 2010–2016 | National Free Pre-Pregnancy Checkups Project (NFPCP) | PGDM | Birth defects (2,908), 6,447,339 | Not reported | Not reported | Maternal age at baseline, higher education, area of residence, smoking status, alcohol consumption, BMI, history of adverse pregnancy outcomes, hypertension, and region of GDP per capita. |
| Yang 2019 [18], United States (Americas) | 2006–2014 | The Perinatal Data System (PDS) in Upstate New York | PGDM, GDM | Any birth defect (5,316), Cyanotic heart disease (755), Cleft lip and palate (446), Cleft palate alone (210), Hypospadias (747), Limb reduction defect (189), 650,914 | Not reported | Live births | Age, smoking during pregnancy, drinking during pregnancy, race, education, employed during pregnancy, pre-pregnancy BMI, depression, and hypertension. |
| Ludvigsson 2018 [19], Sweden (European) | 2003–2015 | The Swedish national diabetes register | T1D | Cardiac defects (17,120), 1,162,323 | ICD-10 codes | Live births | Calendar year, maternal age, country of birth, living with partner, education, parity, body mass index, smoking status, and other autoimmune disease. |
| Arendt 2018 [20], Denmark, Sweden (European) | 1978–2012 | The Danish and Swedish Medical Birth Registers | PGDM, GDM | Hypospadias (12,946), 2,416,248 | ICD-8, ICD-9, ICD-10 codes | Live births | Maternal age at birth in years, parity, maternal years of education, maternal nationality, and calendar year at birth. |
| Kovalenko 2018 [21], Russia (Asian-Pacifc) | 2006–2011 | The Murmansk County Birth Registry (MCBR), the Murmansk Regional Congenital Defects Registry | PGDM | VSD (233), 52,253 | ICD-10 codes | Not reported | Maternal BMI, smoking during pregnancy, aalcohol abuse during pregnancy, drugs abuse during pregnancy, folic acid intake during pregnancy, multivitamins intake during pregnancy, and sex of the baby. |
| Soliman 2018 [22], Qatar (Asian-Pacifc) | 2016–2017 | The PEARL-Peristat Maternal and newborn registry | PGDM, GDM | Congenital anomalies (115), 12,255 | Not reported | Live births | None |
| Billionnet 2017 [23], France (European) | 2012 | The French hospital discharge database (PMSI), the French National Health Insurance database (SNIIRAM) | PGDM (T1D, T2D), GDM | Cardiac malformations (5,768), nervous system malformations (1,185), 796,346 | ICD-10 codes | Not reported | Maternal age. |
| Hoang 2017 [24], United States (Americas) | 1999–2009 | The Texas Birth Defects Registry (TBDR), statewide vital records | PGDM, GDM | Truncus arteriosus (159), LVOT (2,733), Hypoplastic left heart syndrome (731), RVOT (3,805), Pulmonary valve atresia (597), Pulmonary valve stenosis (2,762), Total AVPR (632), 4,207,896 | ICD BPA codes | Live births | Maternal age, race/ethnicity, any hypertension, previous live births, smoking, and BMI. |
| Darke 2016 [25], United Kingdom (European)^*^ | 1998–2010 | The Northern Diabetes in Pregnancy Survey (NorDIP), the Northern Survey of Twin and Multiple Pregnancy (NorSTAMP), the Northern Congenital Abnormality Survey (NorCAS), the Perinatal Morbidity and Mortality Survey (PMMS) | PGDM | Congenital anomaly (479), 12,868 | EUROCAT | Live births, stillbirths | Maternal age at delivery, BMI at first antenatal visit, gestation at first antenatal visit, smoking during pregnancy, parity, ethnicity, index of multiple deprivation tertile and chorionicity. |
| Lai 2016 [26], Canada (Americas)^*^ | 2005–2011 | The Alberta Vital Statistics Birth File, Alberta Congenital Anomalies Surveillance System (ACASS) Database | PGDM, GDM | Congenital anomalies (7,125), 332,864 | ICD-10 codes | Live births, stillbirths | Mother age, First Nations status, parity, and preexisting hypertension, and for stillborn, Apgar score and neonatal death further adjusted for mode of delivery. |
| Leirgul 2016 [27], Norway (European) | 1994–2009 | The Medical Birth Registry of Norway, Oslo University Hospital’s clinical database, the multipurpose research project Cardiovascular Disease in Norway, the Cause of Death Registry | PGDM, GDM | Any CHD (10,575), Heterotaxia (127), Conotruncal defect (895), AVSD (215), APVR (97), LVOT (691), RVOT (466), Septal defect (5,276), 914,427 | ICD-8, ICD-9, ICD-10 codes | Live births, stillbirths, termination of pregnancy for fetal anomaly | Year of birth, mother’s age, and parity. |
| Øyen 2016 [28], Denmark (European) | 1978–2011 | The National Patient Register (NPR), the Medical Birth Register, the Abortion Register, the Causes of Death Register, the Register of Medicinal Product Statistics | PGDM, GDM | Any type of CHD (16,325), Heterotaxia (198), Conotruncal defects (1,626), Truncus arteriosus (142), Transposition of the great arteries (665),Tetralogy of Fallot (611), AVSD (485), APVR (142), LVOT (1,524), Coarctation of aorta (783), RVOT (1,000), valvular pulmonary stenosis (802), VSD (4,523), ASD (2,127), VSD+ASD (342), Septal defects (7,092), 2,025,727 | ICD-8, ICD-10 codes | Live births | Birth year, maternal age, and birth order. |
| Tain 2016 [29], China (Asian-Pacifc) | 2004–2014 | The Birth Certificate Application (BCA) | PGDM, GDM | CAKUT (668), 1,603,794 | Not reported | Live births | Maternal age, birth order, polyhydramnios/oligohydramnios, thalassemia/ hemochromatosis, others illness, gender, and gestational age. |
| Dart 2015 [30], Canada (Americas) | 1996/1997– 2009/2010 | The Manitoba Centre for Health Policy (MCHP), the Diabetes Education Resource for Children and Adolescents (DER-CA) | PGDM, GDM | CAKUT (935), 5,670 | ICD-9 CM, ICD-10 CM codes | Not reported | Maternal age, lowest income quintile, alcohol, illicit drug and RAAS inhibitor use, North (Norman, Burntwood, and Churchill) versus other regional health authorities, and size for gestational age. |
| Liu 2015 [31], Canada (Americas) | 2002–2013 | The Discharge Abstract Database (DAD) | PGDM (T1D, T2D) | Central nervous system, Cardiovascular, Orofacial clefts, Digestive system, Genitourinary, Musculoskeletal, Any congenital anomaly, 2,839,680 | ICD-10 codes | Live births | Maternal age, number of previous births, and year of birth. |
| Liu 2015 [32], China (Asian-Pacifc) | 2009–2011 | Pregnant Women Health Records, Children Health Records | GDM | CHD (1,817), 90,796 | Not reported | Live births | Infant age, sex, birthweight, gestational age, maternal age, pre-pregnancy BMI, pregnant infection, contact with toxic substance, history of using medicines, health history of parents, pregnancy-induced hypertension, pregnant anaemia, history of parent with CHD, genetic history of parents, education of parent, smoking, family income, drinking of parents and decoration during pregnancy. |
| Mavrogenis 2015 [33], Hungary (European) | 1980–1996 | The population-based Hungarian Case–Control Surveillance of Congenital Abnormalities (HCCSCA) | GDM | Hypospadias (3,038), 27,852 | Not reported | Live births | Maternal age and birth order. |
| Csaky-Szunyogh 2014 [34], Hungary (European) | 1980–1996 | The population-based Hungarian Case–Control Surveillance of Congenital Abnormalities (HCCSCA) | GDM | LVOT (302), 38,453 | Not reported | Live births | None |
| Feig 2014 [35], Canada (Americas) | 1996–2010 | The Canadian Institute for Health Information Discharge Abstract Database | PGDM, GDM | Congenital anomalies, 1,109,605 | ICD-9, ICD-10 codes | Not reported | Maternal age. |
| Persson 2014 [36], Sweden (European) | 1998–2007 | The Medical Birth Registry | PGDM, GDM | Major malformation (16,428), 918,671 | Not reported | Live births | Maternal ethnicity, maternal age, BMI, smoking prior to pregnancy, parity, mode of delivery, pre-eclampsia and pregnancy-induced hypertension, infants birthweight, length and gestational age. |
| Vereczkey 2014 [37], Hungary (European) | 1980–1996 | The population-based Hungarian Case–Control Surveillance of Congenital Abnormalities (HCCSCA) | PGDM | VSD (1,659), Single ventricle (75), ASD (471), Transposition of great arteries (307), Conotruncal defects (597), Congenital abnormalities of pulmonary artery (108), Right-sided obstructive defects (200), Hypoplastic left heart (76), Coarctation of aorta (113), Left-sided obstructive defects (302), 41,713 | Not reported | Live births | None |
| Vinceti 2014 [38], Italy (European) | 1997–2010 | The Emilia-Romagna Region Birth Defects Registry (IMER) | PGDM | All anomalies (278), Eye, ear, face and neck (11), Cardiovascular (79), Cleft palate and/or cleft lip (17), Digestive system (22), Genito-urinary (44), Muscolo-skeletal (54), Multiple congenital anomalies (28), Transposition of great vessels (4), Tetralogy of Fallot (12), VSD (39), ASD (13), Hypospadias and ot. penile anom. (19), Renal agenesis and dysplasia (4), Limb defects (total) (20), Polydactyly (9), Syndactyly (7), Oral cleft (17), 12,917 | Not reported | Not reported | Maternal age, province of residence, year and hospital of delivery. |
| Liu 2013 [39], Canada (Americas) | 2002/2003– 2010/2011 | The Discharge Abstract Database (DAD) | PGDM | Heterotaxia (430), Conotruncal defects (2,377), AVSD (634), LVOT (547), RVOT (243), VSD (3,691), ASD (2,888), Multiple defects (622), 2,278,838 | ICD-10 CM codes | Live births | Maternal age, infant sex, parity, rural residence, multifetal pregnancy, tobacco use, alcohol or substance use, obesity, preexisting hypertension, thyroid disorders, congenital heart disease, atherosclerotic heart disease, anemia and related disorders, connective tissue disorders, epilepsy and mood disorders, and region and year of birth. |
| Parker 2013 [40], United States, Canada (Americas) | 1976–2011 | The Slone Epidemiology Center Birth Defects Study | PGDM, GDM | Spina bifida (1,154), 10,593 | Not reported | Live births, fetal deaths, elective terminations | Maternal age, education, race/ethnicity, folic acid intake, and study center. |
| Bell 2012 [41], United Kingdom (European) | 1996–2008 | The Northern Diabetes in Pregnancy Survey (NorDIP), the Northern Congenital Abnormality Survey (NorCAS) | PGDM | Nervous system (785), Neural tube defects (453), Cardiovascular system (2,963), Transposition of great vessels (133), VSD (1,306), Tetralogy of Fallot (99), Pulmonary valve stenosis (247), Digestive system (431), Urinary (986), Musculoskeletal (58), Multiple anomalies (449), Any birth defect (7,733), 401,149 | ICD-10 codes, EUROCAT | Live birth, fetal death at ≥20 weeks' gestation or termination of pregnancy | None |
| Garne 2012 [42], Norway, Denmark, Germany, Netherlands, Belgium, Wales, United Kingdom, Ireland, Switzerland, France, Italy, Spain, Portugal, Malta (European) | 1990–2005 | The population-based surveillance of congenital anomalies | PGDM | Nervous system (7,805), Neural tube defects (3,526), Encephalocele (373), Spina Bifida (1,828), Hydrocephaly (1,758), Eye (1,945), Ear, face and neck (1,471), CHD (26,551), Transposition of great vessels (1,145), Single ventricle (274), VSD (12,751), ASD (6,406), AVSD (672), Tetralogy of Fallot (1,016), Pulmonary valve stenosis (1,929), Hypoplastic left heart (872), Coarctation of aorta (1,248), Oro-facial clefts (5,587), Cleft lip with or without palate (3,411), Cleft palate (2,176), Digestive system (7,207), Diaphragmatic hernia (918), Abdominal wall defects (1,528), Omphalocele (755), Urinary (12,260), Renal dysplasia (1,170), Genital (6,337), Hypospadias (4,885), Limb reduction (2,061), Polydactyly (2,943), Syndactyly (2,178), Musculo-skeletal (4,103), 93,465 | ICD-9, ICD-10 codes, EUROCAT | Live births | Maternal age, year, and registry. |
| Wu 2012 [43], Denmark (European) | 1977–2008 | The Danish Medical Birth Register, the Danish Civil Registration System, the Danish National Hospital Register, the Danish National Diabetes Register | PGDM (T1D, T2D), GDM | Congenital malformation (123,793), 1,781,576 | ICD-8, ICD-10 codes | Not reported | Maternal age, sex, maternal education, maternal marital status, and calendar year. |
| Alverson 2011 [44], United States (Americas) | 1981–1989 | The Baltimore-Washington Infant Study | GDM | CHD (2,525), 5,960 | Not reported | Live births | None |
| Bánhidy 2010 [45], Hungary (European) | 1980–1996 | The population-based Hungarian Case-Control Surveillance System of Congenital Abnormalities | PGDM (T1D, T2D), GDM | Neural tube defects (1,202), Cleft lip ± palate (1,375), Cleft palate (601), Hypospadias (3,038), Exomphalos/gastroschisis (255), Hydrocephaly (314), Ear (354), Cardiovascular (4,480), Limb deficiencies (548), Poly/syndactyly (1,744), Musculoskeletal system (585), Diaphragmatic hernia (244), Multiple congenital anomalies (1,349), Congenital anomalies (22,843), 60,994 | Not reported | Live brith, stillbirth, electively terminated | Maternal age, employment status, birth order, and maternal hypertension. |
| Eidem 2010 [46], Norway (European) | 1999–2004 | The Medical Birth Registry of Norway | T1D | Congenital anomaly (10,126), CHD (3,330), 350,961 | ICD-10 codes, EUROCAT | Not reported | Maternal age, parity, gender, maternal smoking in pregnancy, maternal education, European origin, and year of birth. |
| Fadl 2010 [47], Sweden (European) | 1991–2003 | The Swedish Medical Birth Register (MBR) | GDM | Major malformations (22,738), 1,260,297 | Not reported | Not reported | Maternal age, BMI, parity, chronic hypertensive disorder, smoking habits, and ethnicity. |
| Peticca 2009 [48], Canada (Americas) | 2005–2006 | The Ontario Niday Perinatal Database | PGDM (T1D, T2D), GDM | Congenital anomalies (2,324), 120,604 | Not reported | Not reported | Maternal age, multiple birth, cigarette smoking in pregnancy, parity, use of assisted reproductive technology, first trimester visit and antenatal care provider. |
| Correa 2008 [49], United States (Americas) | 1997-2003 | National Birth Defects Prevention Study (NBDPS) | PGDM, GDM | All cardiac defects (3,519), LVOT (148), RVOT (118), 17,925 | Not reported | Live births, stillbirths, terminations | Maternal age, race/ethnicity, entry into prenatal care, BMI, study center, and household income. |
| Macintosh 2006 [50], England, Wales, Northern Ireland (European) | 2002–2003 | Maternal and Child Health (CEMACH) | PGDM | Major anomalies, Nervous system, Neural tube defects, Eye, CHD, Cleft palate, Digestive system, Internal urogenital system, External genital system, Limb, musculoskeletal,and connective tissue, 620,841 | ICD-10 codes, EUROCAT | Live births, stillbirths | None |
| Yang 2006 [51], Canada (Americas) | 1988–2002 | The NS Atlee Perinatal Database | PGDM | Major congenital anomalies (4,672), Cardiac (1,172), VSD (521), Musculoskeletal (1,623), Central nervous system (282), Spina bifida (18), Ear, nose, and throat (373), Genitourinary (662), Hypospadias complex (632), 151,105 | Not reported | Live births, stillbirths | Maternal smoking and age. |
| Anderson 2005 [52], United States (Americas) | 1997–2001 | The population-based Texas Birth Defects Monitoring Program | PGDM, GDM | Anencephaly (239), Spina bifida (367), Holoprosencephaly (90), Hydrocephaly (239), 974 | ICD-9 codes | Live births, late fetal deaths, elective terminations | Ethnicity, BMI, age, maternal education, and periconceptional folic acid use. |
| Nielsen 2005 [53], Hungary (European) | 1980–1996 | The populationbased Hungarian Congenital Abnormality Registry | PGDM | Renal a- or dysgenesis (104), 60,994 | Not reported | Live brith, stillbirth, electively terminated | Maternal age, birth order, and use of antipsychotic drugs during pregnancy. |
| Sharpe 2005 [54], Australia (Asian-Pacifc) | 1986–2000 | The South Australian Birth Defects Register (SABDR) | PGDM | Congenital Anomalies (14,353), Musculoskeletal (4,102), Limb reduction defects (162), Abdominal wall anomalies (163), Urogenital (3,832), Hypospadias (948), Renal agenesis/dysgenesis (86), Cardiovascular (2,418), Transposition of great vessels (164), Oro-facial clefts (469), Nervous system (283), Neural tube defects (77), Hydrocephalus (131), 282,260 | ICD-9 codes | Live births, stillbirths | Mother’s age, ethnicity, place of birth, and year of birth. |
| Loffredo 2001 [55], Columbia (Asian-Pacifc) | 1981–1989 | The Baltimore-Washington Infant Study | PGDM | Cardiovascular malformations (1,250), Transposed great arteries (211), VSD (563), LVOT (425), RVOT (331), 6,949 | ICD codes | Live births | Maternal age, adiposity index, subfertility, hypertension, months of prenatal care, previous miscarriage(s), and previous birth(s). |
| Croen 2000 [56], United States (Americas) | 1993–1996 | The California Birth Defects Monitoring Program (CBDMP) | GDM | Holoprosencephaly (48), 155 | Not reported | Live births, fetal deaths (≥20 weeks of gestation), terminations | None |
| Von Kries 1997 [57], Germany (European) | 1988–1993 | Perinatalerhebung Nordrhine | PGDM | Malformations (7,235), 590,560 | Not reported | Not reported | None |
| Janssen 1996 [58], United States (Americas) | 1984–1991 | The Washington State certificate | PGDM, GDM | Skeletal (112), Cleft lip ± palate (30), Neural-tube defects (7), Heart malformations (86), congenital malformation (411), 19,366 | Not reported | Live births | Mother's age, race, smoking status, and year of child's birth. |
| Becerra 1990 [59], United States (Americas) | 1968–1980 | The Atlanta Birth Defects Case-Control Study | T1D | Cardiovascular malformations, 7,133 | Not reported | Live births, stillbirths | Race, hospital and year of birth, maternal education, maternal age, and maternal history of other chronic illnesses, and alcohol intake during the first trimester of pregnancy. |
| Abbreviations: ASD, Atrial septal defect; AVSD, Atrioventricular septal defect; APVR, Anomalous pulmonary venous return; BPA, British Pediatric Association; BMI, Body mass index; CHD, Congenital heart defects; CAKUT, Congenital anomalies of the Kidney and Urinary Tract; EUBOCAT, European Surveillance of Congenital Anomalies; GDM, Gestational diabetes mellitus; ICD-8, International Classification of Diseases, Eighth Revision; ICD-9, International Classification of Diseases, Ninth Revision; ICD-9-CM, International Classification of Diseases, Ninth Revision, Clinical Modification; ICD-10, International Classification of Diseases, Tenth Revision; ICD-10-CA, International Classification of Diseases, Tenth Revision, in Canada; ICD-10-CM, International Classification of Diseases, Tenth Revision, Clinical Modification; LVOT, Left ventricular outflow tract; PGDM, Pre-gestational diabetes mellitus; RVOT, Right ventricular outflow tract; T1D, Type 1 diabetes; T2D, Type 2 diabetes; VSD, Ventricular septal defect; VPS, Valvular pulmonary stenosis.  *Studies included twin pregnancies as participants. | | | | | | | |

References

1.Arendt LH, Pedersen LH, Pedersen L, Ovesen PG, Henriksen TB, Lindhard MS, et al. Glycemic Control in Pregnancies Complicated by Pre-Existing Diabetes Mellitus and Congenital Malformations: A Danish Population-Based Study. Clin Epidemiol. 2021;13:615–26.

2.Bayoumi MAA, Masri RM, Matani NYS, Hendaus MA, Masri MM, Chandra P, et al. Maternal and neonatal outcomes in mothers with diabetes mellitus in qatari population.BMC Pregnancy Childbirth. 2021;21(1):651

3.Schraw JM, Langlois PH, Lupo PJ. Comprehensive assessment of the associations between maternal diabetes and structural birth defects in offspring: a phenome-wide association study. Ann Epidemiol. 2021;53:14–20.e8.

4.Allen LA, Cannings-John RL, Evans A, Thayer DS, French R, Paranjothy S, et al. Pregnancy in teenagers diagnosed with type 1 diabetes mellitus in childhood: a national population-based e-cohort study. Diabetologia. 2020;63:799–810.

5.Dyck RF, Karunanayake C, Pahwa P, Stang M, Erickson RL, Osgood ND. Congenital Anomalies of the Kidney and Urinary Tract (CAKUT): An Emerging Relationship With Pregestational Diabetes Mellitus Among First Nations and Non-First Nations People in Saskatchewan-Results From the DIP: ORRIIGENSS Project. Can J Diabetes. 2020:S1499–2671.

6.Lee D, Booth GL, Ray JG, Ling V, Feig DS. Undiagnosed type 2 diabetes during pregnancy is associated with increased perinatal mortality: a large population-based cohort study in Ontario, Canada. Diabet Med. 2020;37:1696–1704.

7.Mowla S, Gissler M, Räisänen S, Kancherla V. Association between maternal pregestational diabetes mellitus and spina bifida: A population-based case-control study, Finland, 2000-2014. Birth Defects Res. 2020;112:186–95.

8.Oliveira-Brancati CIF, Ferrarese VCC, Costa AR, Fett-Conte AC. Birth defects in Brazil: Outcomes of a population-based study. Genet Mol Biol. 2020;43:e20180186.

9.Raitio A, Tauriainen A, Leinonen MK, Syvänen J, Kemppainen T, Löyttyniemi E, et al. Maternal risk factors for gastroschisis: A population-based case-control study. Birth Defects Res. 2020;112:989–95.

10.Seghieri G, Di Cianni G, Seghieri M, Lacaria E, Corsi E, Lencioni C, et al. Risk and adverse outcomes of gestational diabetes in migrants: A population cohort study. Diabetes Res Clin Pract. 2020;163:108128.

11.Tinker SC, Gilboa SM, Moore CA, Waller DK, Simeone RM, Kim SY, et al. Specific birth defects in pregnancies of women with diabetes: National Birth Defects Prevention Study, 1997–2011. Am J Obstet Gynecol. 2020;222:176.e1–11.

12.Wu Y, Liu B, Sun Y, Du Y, Santillan MK, Santillan DA, et al. Association of Maternal Prepregnancy Diabetes and Gestational Diabetes Mellitus With Congenital Anomalies of the Newborn. Diabetes Care. 2020;43:2983–90.

13.Chen L, Wang WJ, Auger N, Xiao L, Torrie J, McHugh NG, et al. Diabetes in pregnancy in associations with perinatal and postneonatal mortality in First Nations and non-Indigenous populations in Quebec, Canada: population-based linked birth cohort study. BMJ Open. 2019;9:e025084.

14.Hildén K, Hanson U, Persson M, Magnuson A, Simmons D, Fadl H. Gestational diabetes and adiposity are independent risk factors for perinatal outcomes: a population based cohort study in Sweden. Diabet Med. 2019;36:151–57.

15.Klungsøyr K, Nordtveit TI, Kaastad TS, Solberg S, Sletten IN, Vik AK. Epidemiology of limb reduction defects as registered in the Medical Birth Registry of Norway, 1970-2016: Population based study. PLoS One. 2019;14(7):e0219930.

16.Liu S, Evans J, MacFarlane AJ, Ananth CV, Little J, Kramer MS, et al. Association of maternal risk factors with the recent rise of neural tube defects in Canada. Paediatr Perinat Epidemiol. 2019;33:145–53.

17.Wei Y, Xu Q, Yang H, Yang Y, Wang L, Chen H, et al. Preconception diabetes mellitus and adverse pregnancy outcomes in over 6.4 million women: A population-based cohort study in China. PLoS Med. 2019;16:e1002926.

18.Yang G-R, Dye TD, Li D. Effects of pre-gestational diabetes mellitus and gestational diabetes mellitus on macrosomia and birth defects in Upstate New York. Diabetes Res Clin Pract. 2019;155:107811.

19.Ludvigsson JF, Neovius M, Söderling J, Gudbjörnsdottir S, Svensson AM, Franzén S, et al. Periconception glycaemic control in women with type 1 diabetes and risk of major birth defects: population based cohort study in Sweden. BMJ. 2018;362:k2638.

20.Arendt LH, Lindhard MS, Henriksen TB, Olsen J, Cnattingius S, Petersson G, et al. Maternal Diabetes Mellitus and Genital Anomalies in Male Offspring: A Nationwide Cohort Study in 2 Nordic Countries. Epidemiology. 2018;29:280–89.

21.Kovalenko AA, Anda EE, Odland JØ, Nieboer E, Brenn T, Krettek A. Risk Factors for Ventricular Septal Defects in Murmansk County, Russia: A Registry-Based Study. Int J Env Res Public Health. 2018;15:1320.

22.Soliman A, Salama H, Al Rifai H, De Sanctis V, Al-Obaidly S, Al Qubasi M, et al. The effect of different forms of dysglycemia during pregnancy on maternal and fetal outcomes in treated women and comparison with large cohort studies. Acta Biomed. 2018;89:11–21.

23.Billionnet C, Mitanchez D, Weill A, Nizard J, Alla F, Hartemann A, et al. Gestational diabetes and adverse perinatal outcomes from 716,152 births in France in 2012. Diabetologia. 2017;60:636–44.

24.Hoang TT, Marengo LK, Mitchell LE, Canfield MA, Agopian AJ. Original Findings and Updated Meta-Analysis for the Association Between Maternal Diabetes and Risk for Congenital Heart Disease Phenotypes. Am J Epidemiol. 2017;186:118–28.

25.Darke J, Glinianaia SV, Marsden P, Bell R. Pregestational diabetes is associated with adverse outcomes in twin pregnancies: a regional register-based study. Acta Obstet Gynecol Scand. 2016;95:339–46.

26.Lai FY, Johnson JA, Dover D, Kaul P. Outcomes of singleton and twin pregnancies complicated by pre-existing diabetes and gestational diabetes: A population-based study in Alberta, Canada, 2005–11. J Diabetes. 2016;8:45–55.

27.Leirgul E, Brodwall K, Greve G, Vollset SE, Holmstrøm H, Tell GS, et al. Maternal Diabetes, Birth Weight, and Neonatal Risk of Congenital Heart Defects in Norway, 1994–2009. Obstet Gynecol. 2016;128:1116–25.

28.Øyen N, Diaz LJ, Leirgul E, Boyd HA, Priest J, Mathiesen ER, et al. Prepregnancy Diabetes and Offspring Risk of Congenital Heart Disease: A Nationwide Cohort Study. Circulation. 2016;133:2243–53.

29.Tain Y-L, Luh H, Lin C-Y, Hsu C-N. Incidence and Risks of Congenital Anomalies of Kidney and Urinary Tract in Newborns: A Population-Based Case-Control Study in Taiwan. Medicine. 2016;95:e2659.

30.Dart AB, Ruth CA, Sellers EA, Au W, Dean HJ. Maternal diabetes mellitus and congenital anomalies of the kidney and urinary tract (CAKUT) in the child. Am J Kidney Dis. 2015;65:684–91.

31.Liu S, Rouleau J, León JA, Sauve R, Joseph KS, Ray JG. Impact of pre-pregnancy diabetes mellitus on congenital anomalies, Canada, 2002–2012. Health Promot Chronic Dis Prev Can. 2015;35:79–84.

32.Liu X, Liu G, Wang P, Huang Y, Liu E, Li D, et al. Prevalence of congenital heart disease and its related risk indicators among 90,796 Chinese infants aged less than 6 months in Tianjin. Int J Epidemiol. 2015;44:884–93.

33.Mavrogenis S, Urban R, Czeizel AE. Pregnancy complications in the mothers who delivered boys with isolated hypospadias - a population-based case-control study. J Matern Fetal Neona. 2015;28:489–93.

34.Csáky-Szunyogh M, Vereczkey A, Kósa Z, Gerencsér B, Czeizel AE. Risk factors in the origin of congenital left-ventricular outflow-tract obstruction defects of the heart: a population-based case-control study. Pediatr Cardiol. 2014;35:108–20.

35.Feig DS, Hwee J, Shah BR, Booth GL, Bierman AS, Lipscombe LL. Trends in incidence of diabetes in pregnancy and serious perinatal outcomes: a large, population-based study in Ontario, Canada, 1996–2010. Diabetes Care. 2014;37:1590–96.

36.Persson M, Fadl H. Perinatal outcome in relation to fetal sex in offspring to mothers with pre-gestational and gestational diabetes--a population-based study. Diabet Med. 2014;31:1047–54.

37.Vereczkey A, Gerencsér B, Czeizel AE, Szabó I. Association of certain chronic maternal diseases with the risk of specific congenital heart defects: a population-based study. Eur J Obstet Gynecol Reprod Biol. 2014;182:1–6.

38.Vinceti M, Malagoli C, Rothman KJ, Rodolfi R, Astolfi G, Calzolari E, et al. Risk of birth defects associated with maternal pregestational diabetes. Eur J Epidemiol. 2014; 29:411–18.

39.Liu S, Joseph KS, Lisonkova S, Rouleau J, Van den Hof M, Sauve R, et al. Association between maternal chronic conditions and congenital heart defects: a population-based cohort study. Circulation. 2013;128:583–89.

40.Parker SE, Yazdy MM, Tinker SC, Mitchell AA, Werler MM. The impact of folic acid intake on the association among diabetes mellitus, obesity, and spina bifida. Am J Obstet Gynecol. 2013;209:239.e231–38.

41.Bell R, Glinianaia SV, Tennant PWG, Bilous RW, Rankin J. Peri-conception hyperglycaemia and nephropathy are associated with risk of congenital anomaly in women with pre-existing diabetes: a population-based cohort study. Diabetologia. 2012;55:936–47.

42.Garne E, Loane M, Dolk H, Barisic I, Addor MC, Arriola L, et al. Spectrum of congenital anomalies in pregnancies with pregestational diabetes. Birth Defects Res A Clin Mol Teratol. 2012;94:134–40.

43.Wu CS, Nohr EA, Bech BH, Vestergaard M, Olsen J. Long-term health outcomes in children born to mothers with diabetes: a population-based cohort study. PLoS One. 2012;7:e36727.

44.Alverson CJ, Strickland MJ, Gilboa SM, Correa A. Maternal smoking and congenital heart defects in the Baltimore-Washington Infant Study. Pediatrics. 2011;127:e647–53.

45.Bánhidy F, Acs N, Puhó EH, Czeizel AE. Congenital abnormalities in the offspring of pregnant women with type 1, type 2 and gestational diabetes mellitus: a population-based case-control study. Congenit Anom (Kyoto). 2010;50:115–21.

46.Eidem I, Stene LC, Henriksen T, Hanssen KF, Vangen S, Vollset SE, et al. Congenital anomalies in newborns of women with type 1 diabetes: nationwide population-based study in Norway, 1999-2004. Acta Obstet Gynecol Scand. 2010;89:1403–11.

47.Fadl HE, Ostlund IKM, Magnuson AFK, Hanson USB. Maternal and neonatal outcomes and time trends of gestational diabetes mellitus in Sweden from 1991 to 2003. Diabet Med. 2010;27:436–41.

48.Peticca P, Keely EJ, Walker MC, Yang Q, Bottomley J. Pregnancy outcomes in diabetes subtypes: how do they compare? A province-based study of Ontario, 2005-2006. J Obstet Gynaecol Can. 2009;31:487–96.

49.Correa A, Gilboa SM, Besser LM, Botto LD, Moore CA, Hobbs CA, et al. Diabetes mellitus and birth defects. Am J Obstet Gynecol. 2008;199:237.e1–9.

50.Macintosh MC, Fleming KM, Bailey JA, Doyle P, Modder J, Acolet D, et al. Perinatal mortality and congenital anomalies in babies of women with type 1 or type 2 diabetes in England, Wales, and Northern Ireland: population based study. BMJ. 2006;333:177.

51.Yang J, Cummings EA, O'Connell C, Jangaard K. Fetal and neonatal outcomes of diabetic pregnancies. Obstet Gynecol. 2006;108:644–50.

52.Anderson JL, Waller DK, Canfield MA, Shaw GM, Watkins ML, Werler MM. Maternal obesity, gestational diabetes, and central nervous system birth defects. Epidemiology. 2005;16:87–92.

53.Nielsen GL, Nørgard B, Puho E, Rothman KJ, Sørensen HT, Czeizel AE. Risk of specific congenital abnormalities in offspring of women with diabetes. Diabet Med. 2005;22:693–96.

54.Sharpe PB, Chan A, Haan EA, Hiller JE. Maternal diabetes and congenital anomalies in South Australia 1986-2000: a population-based cohort study. Birth Defects Res A Clin Mol Teratol. 2005;73:605–11.

55.Loffredo CA, Wilson PD, Ferencz C. Maternal diabetes: an independent risk factor for major cardiovascular malformations with increased mortality of affected infants. Teratology. 2001;64:98–106.

56.Croen LA, Shaw GM, Lammer EJ. Risk factors for cytogenetically normal holoprosencephaly in California: a population-based case-control study. Am J Med Genet. 2000;90:320–25.

57.von Kries R, Kimmerle R, Schmidt JE, Hachmeister A, Böhm O, Wolf HG. Pregnancy outcomes in mothers with pregestational diabetes: a population-based study in North Rhine (Germany) from 1988 to 1993. Eur J Pediatr. 1997;156:963–67.

58.Janssen PA, Rothman I, Schwartz SM. Congenital malformations in newborns of women with established and gestational diabetes in Washington State, 1984-91. Paediatr Perinat Epidemiol. 1996;10:52–63.

59.Becerra JE, Khoury MJ, Cordero JF, Erickson JD. Diabetes mellitus during pregnancy and the risks for specific birth defects: a population-based case-control study. Pediatrics. 1990;85:1–9.
