# Supplementary material for: Risks of specific congenital anomalies in offspring of women with diabetes: A systematic review and meta-analysis of population-based studies including over 80 million births
Source: PLoS Med. 2022 Feb 1;19(2):e1003900. doi: 10.1371/journal.pmed.1003900 (PMC8806075; doi:10.1371/journal.pmed.1003900)
Supplement: S1 Text — (DOCX) [file pmed.1003900.s007.docx]

**S1 Text**

This supporting information formed part of the original submission and has been peer reviewed.

We post it as supplied by the authors.

Supplement to: Tie-Ning Zhang, Xin-Mei Huang, Xin-Yi Zhao, Wei Wang, Ri Wen, Shan-Yan Gao. Risks of specific congenital anomalies in offspring of women with diabetes: A systematic review and meta-analysis of population-based studies including over 80 million births

**S1 Text PubMed/Embase search strategy**

Date of search 2021/10/15 PubMed:

14,019 records located.

1. **Search for outcomes (#20)**
2. Congenital Abnormalities[mesh]
3. Abnormality, Congenital
4. Congenital Abnormality
5. Deformities
6. Deformity
7. Congenital Defects
8. Congenital Defect
9. Defect, Congenital
10. Defects, Congenital
11. Abnormalities, Congenital
12. Birth Defects
13. Birth Defect
14. Defect, Birth
15. Fetal Malformations
16. Fetal Malformation
17. Malformation, Fetal
18. Fetal Anomalies
19. Anomaly, Fetal
20. Fetal Anomaly
21. #1 OR #2 OR #3 OR #4 OR #5 OR #6 OR #7 OR #8 OR #9 OR #10 OR #11 OR #12 OR #13 OR #14 OR #15 OR #16OR #17 OR #18 OR #19

**AND**

1. **Search for diabetes (b1 OR b2 OR b3)**

**b1. Search for gestational diabetes mellitus (#28)**

1. Diabetes, Gestational[mesh]
2. Diabetes, Pregnancy-Induced
3. Diabetes, Pregnancy Induced
4. Pregnancy-Induced Diabetes
5. Gestational Diabetes
6. Diabetes Mellitus, Gestational
7. Gestational Diabetes Mellitus
8. #21 OR #22 OR #23 OR #24 OR #25 OR #26 OR #27

**b2. Search for type 2 diabetes (#61)**

1. Diabetes Mellitus, Type 2[mesh]
2. Diabetes Mellitus, Noninsulin-Dependent
3. Diabetes Mellitus, Ketosis-Resistant
4. Diabetes Mellitus, Ketosis Resistant
5. Ketosis-Resistant Diabetes Mellitus
6. Diabetes Mellitus, Non Insulin Dependent
7. Diabetes Mellitus, Non-Insulin-Dependent
8. Non-Insulin-Dependent Diabetes Mellitus
9. Diabetes Mellitus, Stable
10. Stable Diabetes Mellitus
11. Diabetes Mellitus, Type II
12. NIDDM
13. Diabetes Mellitus, Noninsulin Dependent
14. Diabetes Mellitus, Maturity-Onset
15. Diabetes Mellitus, Maturity Onset
16. Maturity-Onset Diabetes Mellitus
17. Maturity Onset Diabetes Mellitus
18. MODY
19. Diabetes Mellitus, Slow-Onset
20. Diabetes Mellitus, Slow Onset
21. Slow-Onset Diabetes Mellitus
22. Type 2 Diabetes Mellitus
23. Noninsulin-Dependent Diabetes Mellitus
24. Noninsulin Dependent Diabetes Mellitus
25. Maturity-Onset Diabetes
26. Diabetes, Maturity-Onset
27. Maturity Onset Diabetes
28. Type 2 Diabetes
29. Diabetes, Type 2
30. Diabetes Mellitus, Adult-Onset
31. Adult-Onset Diabetes Mellitus
32. Diabetes Mellitus, Adult Onset
33. #29 OR #30 OR #31 OR #32 OR #33 OR #34 OR #35 OR #36 OR #37 OR #38 OR #39 OR #40 OR #41 OR #42 OR #43 OR #44 OR #45 OR #46 OR #47 OR #48 OR #49 OR #50 OR #51 OR #52 OR #53 OR #54 OR #55 OR #56 OR #57 OR #58 OR #59 OR #60

**b3. Search for type 1 diabetes (#89)**

1. Diabetes Mellitus, Type 1[mesh]
2. Diabetes Mellitus, Insulin-Dependent
3. Diabetes Mellitus, Insulin Dependent
4. Insulin-Dependent Diabetes Mellitus
5. Diabetes Mellitus, Juvenile-Onset
6. Diabetes Mellitus, Juvenile Onset
7. Juvenile-Onset Diabetes Mellitus
8. IDDM
9. Juvenile-Onset Diabetes
10. Diabetes, Juvenile-Onset
11. Juvenile Onset Diabetes
12. Diabetes Mellitus, Sudden-Onset
13. Diabetes Mellitus, Sudden Onset
14. Sudden-Onset Diabetes Mellitus
15. Type 1 Diabetes Mellitus
16. Diabetes Mellitus, Insulin-Dependent, 1
17. Insulin-Dependent Diabetes Mellitus 1
18. Insulin Dependent Diabetes Mellitus 1
19. Type 1 Diabetes
20. Diabetes, Type 1
21. Diabetes Mellitus, Type I
22. Diabetes, Autoimmune
23. Autoimmune Diabetes
24. Diabetes Mellitus, Brittle
25. Brittle Diabetes Mellitus
26. Diabetes Mellitus, Ketosis Prone
27. Ketosis-Prone Diabetes Mellitus
28. #62 OR #63 OR #64 OR #65 OR #66 OR #67 OR #68 OR #69 OR #70 OR #71 OR #72 OR #73 OR #74 OR #75 OR #76 OR #77 OR #78 OR #79 OR #80 OR #81 OR #82 OR #83 OR #84 OR #85 OR #86 OR #87 OR #88

Date of search 2021/10/15 Embase:

10,970 records located.

1. **Search for outcomes (#21)**
2. 'congenital malformation'/exp
3. 'Congenital abnormalities'
4. 'abnormality, congenital'
5. 'congenital abnormality'
6. 'deformities'
7. 'deformity'
8. 'congenital defects'
9. 'congenital defect'
10. 'defect, congenital'
11. 'Ddefects, congenital'
12. 'abnormalities, congenital'
13. 'birth defects'
14. 'birth defect'
15. 'defect, birth'
16. 'fetal malformations'
17. 'fetal malformation'
18. 'malformation, fetal'
19. 'fetal anomalies'
20. 'anomaly, fetal'
21. 'fetal anomaly'
22. #1 OR #2 OR #3 OR #4 OR #5 OR #6 OR #7 OR #8 OR #9 OR #10 OR #11 OR #12 OR #13 OR #14 OR #15 OR #16OR #17 OR #18 OR #19 #20

**AND**

1. **Search for diabetes (b1 OR b2 OR b3)**

**b1. Search for gestational diabetes mellitus (#30)**

1. 'pregnancy diabetes mellitus'/exp
2. 'diabetes, gestational'
3. 'diabetes, pregnancy-induced'
4. 'diabetes, pregnancy induced'
5. 'pregnancy-induced diabetes'
6. 'gestational diabetes'
7. 'diabetes mellitus, gestational'
8. 'gestational diabetes mellitus'
9. #22 OR #23 OR #24 OR #25 OR #26 OR #27 OR #28 OR #29

**b2. Search for type 2 diabetes (#64)**

1. 'non insulin dependent diabetes mellitus'/exp
2. 'diabetes mellitus, type 2'
3. 'diabetes mellitus, noninsulin-dependent'
4. 'diabetes mellitus, ketosis-resistant'
5. 'diabetes mellitus, ketosis resistant'
6. 'ketosis-resistant diabetes mellitus'
7. 'diabetes mellitus, non insulin dependent'
8. 'diabetes mellitus, non-insulin-dependent'
9. 'non-insulin-dependent diabetes mellitus'
10. 'diabetes mellitus, stable'
11. 'stable diabetes mellitus'
12. 'diabetes mellitus, type ii'
13. 'niddm'
14. 'diabetes mellitus, noninsulin dependent'
15. 'diabetes mellitus, maturity-onset'
16. 'diabetes mellitus, maturity onset'
17. 'maturity-onset diabetes mellitus'
18. 'maturity onset diabetes mellitus'
19. 'mody'
20. 'diabetes mellitus, slow-onset'
21. 'diabetes mellitus, slow onset'
22. 'slow-onset diabetes mellitus'
23. 'type 2 diabetes mellitus'
24. 'noninsulin-dependent diabetes mellitus'
25. 'noninsulin dependent diabetes mellitus'
26. 'maturity-onset diabetes'
27. 'diabetes, maturity-onset'
28. 'maturity onset diabetes'
29. 'type 2 diabetes'
30. 'diabetes, type 2'
31. 'diabetes mellitus, adult-onset'
32. 'adult-onset dabetes mellitus'
33. 'diabetes mellitus, adult onset'
34. #31 OR #32 OR #33 OR #34 OR #35 OR #36 OR #37 OR #38 OR #39 OR #40 OR #41 OR #42 OR #43 OR #44 OR #45 OR #46 OR #47 OR #48 OR #49 OR #50 OR #51 OR #52 OR #53 OR #54 OR #55 OR #56 OR #57 OR #58 OR #59 OR #60 #61 #62 #63

**b3. Search for type 1 diabetes (#93)**

1. 'non insulin dependent diabetes mellitus'/exp
2. 'diabetes mellitus, type 1'
3. 'diabetes mellitus, insulin-dependent'
4. 'diabetes mellitus, insulin dependent'
5. 'insulin-dependent diabetes mellitus'
6. 'diabetes mellitus, juvenile-onset'
7. 'diabetes mellitus, juvenile onset'
8. 'juvenile-onset diabetes mellitus'
9. 'iddm'
10. 'juvenile-onset diabetes'
11. 'diabetes, juvenile-onset'
12. 'juvenile onset diabetes'
13. 'diabetes mellitus, sudden-onset'
14. 'diabetes mellitus, sudden onset'
15. 'sudden-onset diabetes mellitus'
16. 'type 1 diabetes mellitus'
17. 'diabetes mellitus, insulin-dependent, 1'
18. 'insulin-dependent diabetes mellitus 1'
19. 'insulin dependent diabetes mellitus 1'
20. 'type 1 diabetes'
21. 'diabetes, type 1'
22. 'diabetes mellitus, type i'
23. 'diabetes, autoimmune'
24. 'autoimmune diabetes'
25. 'diabetes mellitus, brittle'
26. 'brittle diabetes mellitus'
27. 'diabetes mellitus, ketosis prone'
28. 'ketosis-prone diabetes mellitus'

#65 OR #66 OR #67 OR #68 OR #69 OR #70 OR #71 OR #72 OR #73 OR #74 OR #75 OR #76 OR #77 OR #78 OR #79 OR #80 OR #81 OR #82 OR #83 OR #84 OR #85 OR #86 OR #87 OR #88 OR #89 OR #90 OR #91 OR #92
