# Supplementary material for: Risks of specific congenital anomalies in offspring of women with diabetes: A systematic review and meta-analysis of population-based studies including over 80 million births
Source: PLoS Med. 2022 Feb 1;19(2):e1003900. doi: 10.1371/journal.pmed.1003900 (PMC8806075; doi:10.1371/journal.pmed.1003900)
Supplement: S2 Text — (DOCX) [file pmed.1003900.s008.docx]

**S2 Text**

This supporting information formed part of the original submission and has been peer reviewed.

We post it as supplied by the authors.

Supplement to: Tie-Ning Zhang, Xin-Mei Huang, Xin-Yi Zhao, Wei Wang, Ri Wen, Shan-Yan Gao. Risks of specific congenital anomalies in offspring of women with diabetes: A systematic review and meta-analysis of population-based studies including over 80 million births

**S2 Text Moose Checklist**

| **Item No** | **Recommendation** | **Reported on Page No** |
| --- | --- | --- |
| Reporting of background should include | | |
| 1 | Problem definition | Introduction, paragraph 2 |
| 2 | Hypothesis statement | Introduction, paragraph 2 |
| 3 | Description of study outcome(s) | Introduction, paragraph 3 |
| 4 | Type of exposure or intervention used | Introduction, paragraph 2-3 |
| 5 | Type of study designs used | Introduction, paragraph 4 |
| 6 | Study population | Introduction, paragraph 4 |
| Reporting of search strategy should include | | |
| 7 | Qualifications of searchers (eg, librarians and investigators) | Methods, Search strategy and inclusion criteria, paragraph 3 |
| 8 | Search strategy, including time period included in the synthesis and key words | S1 Text |
| 9 | Effort to include all available studies, including contact with authors | Methods, Data extraction, paragraph 3 |
| 10 | Databases and registries searched | Methods, Search strategy and inclusion criteria, paragraph 1 |
| 11 | Search software used, name and version, including special features used (eg, explosion) | Methods, Search strategy and inclusion criteria, paragraph 1 |
| 12 | Use of hand searching (eg, reference lists of obtained articles) | Methods, Search strategy and inclusion criteria, paragraph 1 |
| 13 | List of citations located and those excluded, including justification | S2 Table and S4 Table |
| 14 | Method of addressing articles published in languages other than English | Methods, Search strategy and inclusion criteria, paragraph 1 |
| 15 | Method of handling abstracts and unpublished studies | Methods, Search strategy and inclusion criteria, paragraph 1 |
| 16 | Description of any contact with authors | Methods, Data extraction, paragraph 3 |
| Reporting of methods should include | | |
| 17 | Description of relevance or appropriateness of studies assembled for assessing the hypothesis to be tested | Methods, Statistical analysis |
| 18 | Rationale for the selection and coding of data (eg, sound clinical principles or convenience) | Methods, Search strategy and inclusion criteria, paragraph 2 |
| 19 | Documentation of how data were classified and coded (eg, multiple raters, blinding and interrater reliability) | Methods, Data extraction, paragraph 1-3 |
| 20 | Assessment of confounding (eg, comparability of cases and controls in studies where appropriate) | Methods, Statistical analysis |
| 21 | Assessment of study quality, including blinding of quality assessors, stratification or regression on possible predictors of study results | Methods, Risk of bias and study quality |
| 22 | Assessment of heterogeneity | Methods, Statistical analysis |
| 23 | Description of statistical methods (eg, complete description of fixed or random effects models, justification of whether the chosen models account for predictors of study results, dose-response models, or cumulative meta-analysis) in sufficient detail to be replicated | Methods, Statistical analysis |
| 24 | Provision of appropriate tables and graphics | None |
| Reporting of results should include | | |
| 25 | Graphic summarizing individual study estimates and overall estimate | Results, Search results and study characteristics and Table 2-3 |
| 26 | Table giving descriptive information for each study included | S4 Table |
| 27 | Results of sensitivity testing (eg, subgroup analysis) | Results, Subgroup, meta-regression, and sensitivity analyses |
| 28 | Indication of statistical uncertainty of findings | None |
| Reporting of discussion should include | | |
| 29 | Quantitative assessment of bias (eg, publication bias) | None |
| 30 | Justification for exclusion (eg, exclusion of non-English language citations) | None |
| 31 | Assessment of quality of included studies | Discussion, Strengths and limitations, paragraph 2 |
| Reporting of conclusions should include | | |
| 32 | Consideration of alternative explanations for observed results | Conclusion section |
| 33 | Generalization of the conclusions (ie, appropriate for the data presented and within the domain of the literature review) | Conclusion section |
| 34 | Guidelines for future research | Conclusion section |
| 35 | Disclosure of funding source | Funding section |

*From*: Stroup DF, Berlin JA, Morton SC, et al, for the Meta-analysis Of Observational Studies in Epidemiology (MOOSE) Group. Meta-analysis of Observational Studies in Epidemiology. A Proposal for Reporting. *JAMA*. 2000;283(15):2008-2012. doi: 10.1001/jama.283.15.2008.
