# Supplementary material for: Risks of specific congenital anomalies in offspring of women with diabetes: A systematic review and meta-analysis of population-based studies including over 80 million births
Source: PLoS Med. 2022 Feb 1;19(2):e1003900. doi: 10.1371/journal.pmed.1003900 (PMC8806075; doi:10.1371/journal.pmed.1003900)
Supplement: Attachment — Submitted filename: Comments Plos Medicine response_2021_10_28.docx [file pmed.1003900.s010.docx]

***Department of Clinical Epidemiology***

***Clinical Research Center***

***Shengjing Hospital of China Medical University***

***No. 36, San Hao Street, Shenyang***

***Liaoning 110004***

***P. R. China***

10/28/21

**Re:** **Risks of specific congenital anomalies in offspring of women with diabetes: a systematic review and meta-analysis of population-based studies of over 80 million births (PMEDICINE-D-21-01554R1)**

Dear editor,

Thank you for decision letter, informing us that our manuscript is potentially acceptable for publication in *PLoS Medicine* after a revision. We would like to thank reviewers and editor for their helpful comments and suggestions. We have carefully addressed their concerns as outlined in the attached file. Point-by-point responses are followed by this cover letter. We hope that we have adequately addressed all reviewers’ and editor’s concerns and have made this revised manuscript acceptable for publication in *PLoS Medicine*.

Sincerely,

Shan-yan Gao, M.D., Ph.D.

Department of Clinical Epidemiology

Clinical Research Center

Shengjing Hospital of China Medical University

**Authors’ Responses to Reviewers’ Comments**

**Requests from the editors:**
1. Data availability statement: PLOS Medicine requires that the de-identified data underlying the specific results in a published article be made available, without restrictions on access, in a public repository or as Supporting Information at the time of article publication, provided it is legal and ethical to do so. Please see the policy at
<http://journals.plos.org/plosmedicine/s/data-availability> and FAQs at http://journals.plos.org/plosmedicine/s/data-availability#loc-faqs-for-data-policy

It seems as if the data underlying the analyses are provided in the metadata files provided, however, there do not seem to be any descriptions of these files.
**Reply:** Thank you for your comment. We have uploaded our metadata files, which are available at: <https://zenodo.org/record/5595068#.YXVsj55BzD4.> Additionally, we have also added the Data availability statement in our revised manuscript.

2. Author Summary: Please move the Author Summary to follow the Abstract.
**Reply:** Thank you for your suggestion. We have moved the Author Summary to follow the Abstract in the updated manuscript. Please see Page 5-6.

3. Author Summary (and throughout): Please temper statements of primacy with “To the best of our knowledge, this is the first comprehensive systematic review…” or similar.
**Reply:** Thank you for your useful suggestion. We have added the statements of primacy with "To the best of our knowledge, this is the first comprehensive systematic review…" in the updated manuscript. Please see Page 5 Line 79-80.

4. Author Summary (and throughout): Please define all abbreviations at first use in the text (RR).
**Reply:** Thank you for your suggestion. We have defined all abbreviations at first use in the text, including relative risk (RR).

5. Abstract: Please note the eligibility criteria, including language restrictions, of included studies.
**Reply:** Thank you for your suggestion. We have carefully revised our Abstract section, and described eligibility criteria, including language restrictions, of included studies. Please see Page 3 Line 40-41.

6. Abstract: Methods and Findings: Please quantify the main results (with 95% CIs and p values).
**Reply:** Thank you for your suggestion. We have quantified the main results (with 95% CIs and p values). Please see the revised Abstract.

7. Abstract: Methods and Findings: In the last sentence of the Abstract Methods and Findings section, please describe the main limitation(s) of the study's methodology.
**Reply:** Thank you for your suggestion. We have carefully revised our Abstract section, and described the main limitation(s) of the study's methodology in the last sentence of the Abstract Methods and Findings section. Please see Page 4 Line 60-62.

8. Abstract: Conclusion: Please address the study implications without overreaching what can be concluded from the data; the phrase "In this study, we observed ..." may be useful.
**Reply:** Thank you very much for your useful suggestion. We have carefully revised our Conclusion section and used the phrase "In this study, we observed ..." in the updated manuscript. Please see Page 4 Line 64-69.

9. Introduction: Lines 121-124: Here and throughout, please revise to avoid causal language and please refer to associations instead: “...examined the extent to which types of maternal diabetes (i.e. pregestational or gestational) are associated with increased risk of CAs in offspring.” or similar.
**Reply:** Thank you for your suggestion. We have revised to avoid causal language and referred to associations instead: "...examined the extent to which types of maternal diabetes (i.e. pre-gestational or gestational) are associated with increased risk of CAs in offspring" in the revised manuscript. Please see Page 8 Line 134-136.

10. Methods: Please update your search to the present time (currently search has an end date of December 2020).
**Reply:** Thank you for your suggestion. We have updated our search from inception to October 15, 2021. Please see the updated Figure 1 and the search strategy as well as inclusion criteria section in the part of Method. Details of the full search strategy are updated and available in S1 Text.

11. Methods: Line 154: Please reference the PRISMA flowchart.
**Reply:** Thank you for your suggestion. We have revised the sentence as follows:

"The literature review and study selection process referenced the PRISMA flowchart." Please see Page 10 Line 163-164.

12. Methods: Line 208-209: Please provide more detail on how adequacy of controlling for confounders was determined.
**Reply:** Thank you so much for your comments. Actually, we think there was no rationale for the category. In our revised manuscript, we performed subgroup analyses to explore causes of heterogeneity by adjustment for confounders [i.e., maternal age (yes, no), race/ethnicity (yes, no), body mass index (yes, no), education (yes, no), smoking/alcohol drinking (yes, no), parity (yes, no), and pregnancy complications (yes, no)] instead of controlling for confounders (adequate, not adequate). Please see Page 12 Line 214-217 and Table E-F in S1 Table.

13. Results: For the main results presented in the tables and in the text, please report both 95% CIs and p values.
**Reply:** Thank you for your suggestion. For the main results, we have presented in the tables (Please see Table 2-3) and in the text, reporting both 95% CIs and p values.

14. Results: Line 285-288: Please avoid language implying causality, and please refer to associations here: “...statistically significant effect of maternal type 1 and type 2 diabetes on CHD…” and “Notably, we found that maternal PGDM increased the risk of all available specific types of CHD…”
**Reply:** Thank you for your suggestion. We have revised the sentences as follows:

"...maternal type 1 and type 2 diabetes were associated with increased risks of CHD in offspring." Please see Page 16 Line 283-284.

"Notably, we found that maternal PGDM was associated with increased risk of all available specific types of CHD" in our revised manuscript according to your suggestion." Please see Page 16 Line 286-288.

15. Discussion: Please present and organize the Discussion as follows: a short, clear summary of the article's findings; what the study adds to existing research and where and why the results may differ from previous research; strengths and limitations of the study; implications and next steps for research, clinical practice, and/or public policy; one-paragraph conclusion.
**Reply:** Thank you for your suggestion. We have re-presented and re-organized the Discussion. Please see the updated version of manuscript.

16. Discussion: Line 450-452: Here and throughout the discussion, please avoid the use of language implying causality: “Finally, the current study examined the extent to which types of maternal diabetes (i.e., pregestational or gestational) increase risk of CAs in their offspring.”
**Reply:** Thank you for your suggestion. We have revised the sentence here and throughout the discussion as follows:

"Finally, the current study examined the extent to which types of maternal diabetes (i.e., pre-gestational or gestational) are associated with increased risk of CAs in their offspring." Please see Page 27 Line 435-437.

17. S1 Text: Thank you for including the PRISMA and MOOSE checklists. When completing the checklists, please use section and paragraph numbers rather than page numbers to refer to locations within the text.
**Reply:** Thank you for your suggestion. We have updated the PRISMA and MOOSE checklists and used section and paragraph numbers in S1 Text.

**Comments from the reviewers:**
**Reviewer #1:** This systematic review and meta-analysis examines the association between pre-pregnancy diabetes mellitus, gestational diabetes and congenital anomalies. This is a thorough review, well written and comprehensively presented. I have only relatively minor comments.
Comments:
1. Table 1: I suppose type 1 and type 2 diabetes mellitus would always be 'pre-gestational diabetes'. In the category 'pre-gestational diabetes', did the results of all 42 studies relate to a combined any type 1 or type 2 diabetes? Perhaps, instead of 'pre-gestational diabetes', you can use, for instance, 'pre-gestational diabetes (combined type 1 or 2)'. Just a suggestion.
**Reply:** Thank you so much for your comments. We agree with your opinion. Type 1 and type 2 diabetes mellitus would always be 'pre-gestational diabetes'. For instance, pre-gestational diabetes in the included study was defined as reporting type 1 diabetes or type 2 diabetes diagnosed before the index pregnancy (PMID: 31454511). The detail definition of 'Pre-gestational diabetes' or 'pre-existing diabetes mellitus' in each included studies were shown in Table C in S1 Table.

Actually, 'Pre-gestational diabetes' category presented in Table 1were all extracted from 46 studies (according to the suggestion from editor, we updated the search period to the present time, thus the number of included studies has been updated in the revised Table 1), which reported 'pre-gestational diabetes' or 'pre-existing diabetes mellitus' as the exposure of interest. Therefore, in the category 'pre-gestational diabetes', the results of all 46 studies were relate to a combination of any type 1 or type 2 diabetes. We have revised and used 'pre-gestational diabetes (combined type 1 or 2)' at first use in the Abstract and Methods section. Please see Page 3 and Page 9.

2. Similar issue in Table 2: The indentation of subcategories in the table suggests these sub-categories are included in the total above. Can you, please, clarify, for instance, 'LVOT' category includes all LVOT anomalies, perhaps instead of 'LVOT' you can use 'LVOT combined' or 'All LVOT). The same applies to 'Conotruncal defect' - you can use 'Conotruncal defects (any)', etc. Similar issue is in Table 3, most rows look like subcategories. Just a suggestion.

**Reply:** Thank you so much for your comments. Actually, the subcategories in the Table 2-3 are not included in the total above. For instance, the RRs and 95%CI of 'LVOT' category were all extracted from the included studies which reported the outcome named 'LVOT', rather than combined 'coarctation of aorta' or 'hypoplastic left heart'.

3. Page 11, Line 196: Do you mean effect size (instead of effective size)?

**Reply:** Thank you so much for your comments. In previous Page 11, Line 196, we mean effect size as you realize. We have used effect size instead of effective size. Please see Page 12 Line 204.

4. Page 11, line 209: Can you specify adequate and not adequate control for confounding? What set of confounders would you consider adequate adjustment in the studies that adjusted for confounders? They are listed in Supplemental tables (Table D) but what was considered an 'adequate control' is not stated.

**Reply:** Thank you so much for your comments. Actually, we think there was no rationale for the category. In our revised manuscript, we performed subgroup analyses to explore causes of heterogeneity by adjustment for confounders [i.e., maternal age (yes, no), race/ethnicity (yes, no), body mass index (yes, no), education (yes, no), smoking/alcohol drinking (yes, no), parity (yes, no), and pregnancy complications (yes, no)] instead of controlling for confounders (adequate, not adequate). Please see Page 12 Line 214-217 and Table E-F in S1 Table.

5. Fig 1 suggests that after identification of 23,748 records in PubMed and Embase and 1 record extra, the majority was excluded due to duplicates ('records after duplicates removed n=2,096'). You could perhaps place this box with the right side arrow and use the text 'Duplicates removed (n=2,096)'

**Reply:** Thank you for your suggestion. We have placed the box you mentioned with the right side arrow and used the text 'Duplicates removed (n=2,331)'. Please see the updated Figure 1.

6. In sensitivity analyses, strata categories are: before year 2000, between 2000 and 2010, and after 2010. It is not wrong, I am just wondering if there was any rationale for these categories.

**Reply:** Thank you so much for your comments. Regarding this suggestion, we think there was no rationale for above categories. In our revised manuscript, new strata categories are: before 1997, in or after 1997, categorized according to the median as a cutoff value. Please see Page 12 Line 212-213 and Tables E-F in S1 Table.

7. Discussion: In study limitations, it would be nice to address the lack of information on how well the diabetes was controlled (e.g., mothers with well controlled diabetes would be expected to have lower rates of CA). Similarly, information on treatment (e.g., insulin use) was not available in most studies included in the review.

**Reply:** Thank you so much for your comments. We have mentioned the lack of information on how well the diabetes was controlled and on treatment in the limitations section. In addition, we also suggest further investigations should focus on the above questions. Please see Page 28 Line 459-462.

8. In Table 3, a relative risk of gastroschisis is below 1 (i.e., a 'protective effect' of maternal gestational diabetes). Is this consistent across the studies? Why would there be a negative association? It would be nice to mention in the discussion.

**Reply:** Thank you so much for your comments. Actually, we think there should be a positive association between maternal GDM and gastroschisis in offspring. The reasons for which maternal GDM was inversely associated with the risk of gastroschisis in the present study are currently unknown, which warrants confirmation and further investigation in future studies. Residual confounding may contribute to the current inverse association, but still needs further confirmation. We have mentioned it in the limitations section. Please see Page 28 Line 462-465.

9. You mentioned ascertainment of maternal diabetes in the limitations. Besides varying criteria for GDM, pre-pregnancy diabetes is sometimes unrecognized and discovered only during pregnancy as GDM, which could lead to overestimation of relative risks associated with GDM.

**Reply:** Thank you for your suggestion. We have mentioned it in the limitations section. Please see Page 27 Line 443-447.

10. In addition to the limitations, the ascertainment of some congenital anomalies may vary substantially between studies. Some CAs are easy to ascertain (e.g., anencephaly), while some may not be recognized immediately after birth and be discovered only later in infancy (e.g., milder atrial septal defects). This also contributes to the heterogeneity of the results.

**Reply:** Thank you for your suggestion. We have mentioned it in the limitations section. Please see Page 27-28 Line 447-450.

11. The manuscript needs some editing.

**Reply:** Thank you for your suggestion. We have revised and edited our manuscript carefully according to the comments from all reviewers and the editor. Additionally, our manuscript has been also edited by a native speaker for proofreading.

Minor comments:
Page 16 Line 287: "Notably, we found that maternal PGDM increased the risk of all available specific types of CHD in the present study". I suggest "Notably, we found that maternal PGDM increased the risk of all specific types of CHD available to examine in the present study".
**Reply:** Thank you for your suggestion. We have revised the sentence. Please see Page 16 Line 286-288.

**Reviewer #2:** This is a well-conducted systematic review and meta-analysis on the risks of specific congenital anomalies in offspring of diabetic mothers using population-based studies. The study design, datasets, statistical methods and analyses, and presentation (tables and figures) and interpretation of the results are mostly adequate and of a good standard. Only a few minor issues needing attention.

1) In table 1, For Type of maternal diabetes, the number of studies doesn't add to the 56 total by whatever combination of Pregestational diabetes, Type 1 diabetes, Type 2 diabetes, and Gestational diabetes. There must be overalps. Can authors make it clearer? especially what is the exact overlap and in which subtype?
**Reply:** Thank you for your comments. Actually, there are overlaps of different kinds of diabetes as you realize. In the revised manuscript, 'Pregestational diabetes' category were all extracted from 46 studies which reported 'Pregestational diabetes' as the exposure of interest. Similarly, 'Gestational diabetes' category were all extracted from 37 studies which reported 'Gestational diabetes' as the exposure of interest. Considering 59 studies we included in final (according to the suggestion from editor, we updated the search period to the present time, and thus we included 3 more studies in the revised manuscript), there are some overalps, which means some studies we included focus on both 'Pregestational diabetes' and 'Gestational diabetes'. Of 46 studies which reported 'Pregestational diabetes' as the exposure of interest, a total of 28 studies also evaluated the associations between maternal 'Gestational diabetes' and risk of any congenital anomalies in offspring; 7 studies also evaluated the associations between maternal 'Type 1 diabetes' as well as 'Type 2 diabetes' and risk of any congenital anomalies in offspring. We have mentioned the overalps studied in the footnote in the updated Table 1. More details of 'Type of diabetes' and the exact number are provided in Table C in S1 Table.

2) Meta-regression analyses were nicely done to show significant differences in RRs of CAs/CHD in PGDM versus GDM. However, as the selected studies are over about 50 years' period, could the meta-analysis be done over time to show whether any difference in RRs of CAs/CHD over time?
**Reply:** Thank you for your suggestion. We have performed meta-regression analyses to show whether any difference in RRs of CAs/CHD over time by year of enrollment (before 1997, in or after 1997) categorized according to the median as a cutoff value. However, results of the meta-regression analyses showed that there was no difference in RRs of CAs/CHD over time (all *P* _meta-regression_ ＞0.05). Please see Table E-F in S1 Table.

3) On page 15, High I-squared of 90% was shown on the results for overall CAs in offspring of mothers with PGDM. Similar high I-squared were also found in the results with T1D (82.5%) and GDM (76%). This happened even after using the random effect model. Can authors explain this high heterogeneity and potential impact on the results?
**Reply:** Thank you very much for your suggestion. This also happened even after using the fix effect model. To further explore the source of heterogeneity, we performed subgroup and meta-regression analyses in the predefined subgroups, including study location, year of enrollment, study sample size, and adjustment for confounders (Table E in S1 Table). We observed that the high I^2^ of 76% shown on the results for overall CAs in offspring of mothers with GDM reduced to moderate (I^2^ of 59.7%) heterogeneity after adjustment for smoking/alcohol drinking (Table E in S1 Table). But the heterogeneity of RRs remained high for the outcomes of overall CAs in offspring of mothers with PGDM. We have mentioned the high levels of statistical heterogeneity and discussed four causes of high heterogeneity in the limitation section. Please see Page 27-28 Line 442-456.

**Reviewer #3:** Overview
This is a very extensive systematic review of the risks of specific congenital anomalies and GDM and PGDM. The authors have done an excellent job in collating all this information.

Major Comments
1. Table 3 - what is the bottom line based on 4 and 2 studies ? How does it relate to data in Figure E
**Reply:** Thank you so much for your comment. We think the results on Table 3 were not related to data in Figure E. We explained it as follows:

The bottom line based on 6 and 3 studies (according to the suggestion from editor, we updated the search period to the present time, and thus we included 3 more studies in the revised manuscript) in Table 3 showed the results on the associations between maternal diabetes (pre-gestational or gestational) and the risk of major congenital anomalies. Major congenital anomalies were identified and defined: ICD-10 codes Q00-Q89 excluding minor congenital malformations (Q10, Q162, Q17-Q182, Q184-Q189, Q250, Q270, Q381, Q515, Q516, Q520-Q527, Q53, Q664-Q666, Q69, Q70, Q81-Q84, Q950-Q952, Q954, Q955, Q959 (PMID: 28082367). Actually, the effect size of 'Major congenital anomalies' category was all extracted from the reported RRs and 95%CI of the included studies which reported the outcome named 'Major congenital anomalies'.

Figure E showed the results on the associations between maternal diabetes and the risk of overall congenital anomalies. Similarly, the effect size of 'Overall congenital anomalies' category was all extracted from the reported RRs and 95%CI of the included studies which reported 'any birth defect', 'all anomalies', or 'congenital malformations' as outcome of interest, rather than combined any other type-specific congenital anomalies.

2. Lines 327 - I do not understand what is being done here in the sensitivity analyses- the authors say that they reduced the I^2^ by excluding individual studies, with one range going from 55% to 82.9%. Does this not imply that one single study was responsible for a large amount of the variation and in which case more information should be given about that individual study and why it was so discrepant from the other studies.
**Reply:** Thank you so much for your comment. The sensitivity analyses evaluated the effect of omitting one study at a time from each analysis. In sensitivity analysis, we observed that high I^2^ of 81.2% shown on the results for CHD in offspring of mothers with GDM reduced to moderate I^2^ of 55.0% after excluding the study by Billionnet et al (PMID: 28197657). We agree with your opinion. The one single study (Billionnet et al) was responsible for a large amount of the variation. In this regard, we think there were some causes of heterogeneity. First, Billionnet et al. defined GDM at least one of the following criteria: insulin dispensed at least once during pregnancy; at least 200 glucose strips dispensed during pregnancy on at least 2 different occasions; a diagnosis of diabetes recorded during the delivery admission (ICD-10 codes E10–E14, O240–O244, O249). Since guidelines for screening and diagnosis of women with GDM have been changed in a number of countries over the past decade, ascertainment of maternal GDM was inconsistent with other included study (Details please see Table C in S1 Table). Second, Billionnet et al. did not reported the information about types of birth. The lack of information on live births, stillbirths and terminations of pregnancy for fetal anomaly could introduce selection bias and underestimate the strength of the associations between maternal diabetes and risk of CAs in offspring. Third, Billionnet et al. only adjusted for maternal age in their adjusted models. We considered that the study with not adjustment also caused heterogeneity. We have mentioned the causes of heterogeneity in the limitation section. Please see Page 27-28 Line 442-456.

3. Lines 389 - The authors state "Therefore, blood glucose during the first trimester in a mother with GDM could be normal or just slightly elevated, leading to minimal influence on organogenesis." And yet they present results showing that there is an increased risk of anomalies in mothers with GDM. This needs more clarification as to why you would expect an increased risk in GDM

**Reply:** Thank you so much for your comment. Although blood glucose during the first trimester in a mother with GDM could be normal or just slightly elevated, the previous studies have showed that women who develop GDM during pregnancy usually have evidence of metabolic dysfunction before pregnancy, such as pancreatic β-cell defects and increased insulin resistance. These reasons could partly explain the positive association between GDM and congenital anomalies, but still need further studies to elucidate the potential mechanisms. We have added these sentence in the update discussion. Please see Page 25 Line 389-393.

4. The paper concludes that "timely screening for diabetes in women who are pregnant or planning to be pregnant provides a window of opportunity to prevent CAs in offspring.". However it is unclear from this analysis how such prevention would occur. Was there any data in the studies about how well controlled the blood glucose levels were in the mothers - comparing studies with well controlled vs less well controlled would be extremely useful to determine the possibility of prevention. As the authors make statements about the possibility of prevention they need to investigate this in their data. If it is not possible it should be flagged up as an area that needs further work.
**Reply:** Thank you so much for your comments. We agree with your opinion. It is unclear from this analysis how such prevention would occur. Information on treatment (e.g., insulin use) or how well controlled the blood glucose levels were in the mothers - comparing studies with well controlled *vs* less well controlled were not available in most studies included in the current study. It would be nice to address the lack of information in further work. We have mentioned it in the limitation section. Please see Page 28 Line 459-462. In addition, we have revised the Conclusion section. Please see Page 28-29 Line 469-475.

5. I think it is not correct to include all categories of exposed women in the same analysis eg in Fig E. GDM,PGDM,T1 Diabetes and T2 Diabetes are all included and an overall estimate is produced. There is in expectation differences between PGDM and GDM so it is not relevant to combine them. 4 separate plots will make it easier to see how each individual study varies from the combined estimate for all the studies with the same exposure. I think analysing the studies separately may also slightly alter the estimates as the distribution of variances will be altered. The x-axis scale should also be more informative than just 0.1,0 and 10. Consider how to display study by "Oliveria-Brancali" as it is clearly a small study and is causing all other studies to appear very compact.
**Reply:** Thank you for your comment. According to your suggestion, we have deleted Figure E-G in S1 Fig, and provided 4 separate plots (for overall CAs in offspring of mothers with PGDM, GDM, T1 Diabetes, and T2 Diabetes) and 4 separate plots (for CHD in offspring of mothers with PGDM, GDM, T1 Diabetes, and T2 Diabetes) to make it easier to see how each individual study varies from the combined estimate for all the studies with the same exposure. Of these forest plots, 2 separate plots (for overall CAs in offspring of mothers with PGDM and GDM) and 2 separate plots (for CHD in offspring of mothers with PGDM and GDM) are included in the main manuscript (Figure 2-5). The remaining separate plots are shown in Supporting information files. Please see Fig K1, K2, J1 and J2 in S1 Fig.

6. Forest plots are an important part of systematic reviews and I therefore suggest that revised Figure E is included in the main manuscript.
**Reply:** Thank you for your suggestion. The revised Figure E and Figure G is included in the main manuscript (Figure 2-5). The remaining separate plots were shown in Supporting information. Please see Fig K1, K2, J1 and J2 in S1 Fig.

7. I would prefer to see the forest plots for the different anomalies rather than the funnel plots in figure F
**Reply:** Thank you for your suggestion. We have provided forest plots for the different anomalies in Supporting information file. Please see Fig 2-5 in the main manuscript and Fig G1-G18, H1-H25, I1-I15, J1-J22, K1-K2, J1 and J2 in S1 Fig.

Minor Comments
Table 1 : Years should be categorised Before 2000, 2000-2009 and In or after 2010

**Reply:** Thank you so much for your comments. Actually, we think there was no rationale for these categories. In our revised manuscript, new strata categories are: before 1997, in or after 1997, categorized according to the median as a cutoff value. Please see Page 12 Line 212-213 and Table E-F in S1 Table.

Table 2 and Table 3: would it be possible to give the numbers of each specific defect in order to have a feeling for how large the samples are.
**Reply:** Thank you so much for your comments. We have given the total numbers of each specific defect in Table 2 and Table 3.

**Reviewer #4:** Briefly, my comments are as follows: This is a meta-analysis on a very large population of pregnant women with PGDM and GDM, assessing the rate of major congenital malformations (congenital anomalies) and cardiac malformations among the offspring of these mothers. The findings are important, but not new. However, since the studied population is very large and the study is well designed, I think that it merits publication in the journal. I have several comments related to the description of embryologic and teratologic issues. First, the authors use "time post conception" yet they call that "time of pregnancy". The usual definition of pregnancy is from the last menstrual period while fertilization generally occurs about two weeks later. Hence, they should state that their definition of pregnancy is from the time of fertilization. In addition, when they describe the heart development (i.e. lines 394-399) they should state the weeks when the cardiac septum develops (weeks 4-7 post fertilization) not just "first 2 months" that is simply incorrect. It is expected to be more careful with embryological and teratological descriptions

**Reply:** Thank you so much for your useful suggestions. First, we have use "time post conception" instead of "time of pregnancy". Please see the updated discussion. Additionally, we have added our definition of pregnancy according to your suggestion. Besides, we also revised our description regarding heart developments. We use "weeks 4-7 post fertilization" instead of "first 2 months". Please see the revised discussion.

The authors make no attempt to explain the increased rate of anomalies in GDM and the differences between PGDM and GDM. This is perhaps not mandatory, but mentioning several times the need for screening for diabetes in pregnant women as a means to prevent the increased rate of malformations need explanations. They should explain that this will enable better glycemic control which might reduce the rate of malformations, especially during organogenesis, and discuss several studies in this direction. The tables and figures are OK. Thanks. Asher Ornoy, MD.

**Reply:** Thank you so much for your suggestions. We have added the sentence "screening for diabetes in pregnant women will enable better glycemic control which might reduce the rate of malformations, especially during organogenesis". Please see the revised discussion in Page 25 Line 399-401.

Furthermore, we discussed more regarding the association between GDM and congenital anomalies. Please see the revised discussion in Page 25.
