# Supplementary material for: Risks of specific congenital anomalies in offspring of women with diabetes: A systematic review and meta-analysis of population-based studies including over 80 million births
Source: PLoS Med. 2022 Feb 1;19(2):e1003900. doi: 10.1371/journal.pmed.1003900 (PMC8806075; doi:10.1371/journal.pmed.1003900)
Supplement: Attachment — Submitted filename: Response Letter_2021_12_15.docx [file pmed.1003900.s011.docx]

***Department of Clinical Epidemiology***

***Clinical Research Center***

***Shengjing Hospital of China Medical University***

***No. 36, San Hao Street, Shenyang***

***Liaoning 110004***

***P. R. China***

12/15/21

**Re:** **Risks of specific congenital anomalies in offspring of women with diabetes: A systematic review and meta-analysis of population-based studies including over 80 million birth (PMEDICINE-D-21-01554R2)**

Dear editor,

Thank you for decision letter, informing us that our manuscript is potentially acceptable for publication in *PLoS Medicine* after a revision. We would like to thank reviewers and editor for their helpful comments and suggestions. We have carefully addressed their concerns as outlined in the attached file. Point-by-point responses are followed by this cover letter. We hope that we have adequately addressed all reviewers’ and editor’s concerns and have made this revised manuscript acceptable for publication in *PLoS Medicine*.

Sincerely,

Shan-yan Gao, M.D., Ph.D.

Department of Clinical Epidemiology

Clinical Research Center

Shengjing Hospital of China Medical University

**Authors’ Responses to Reviewers’ Comments**

**Requests from the editors:**
1. Title: Please revise the title slightly, and please capitalize the first word of the subtitle, after the colon. We suggest: “Risks of specific congenital anomalies in offspring of women with diabetes: A systematic review and meta-analysis of population-based studies including over 80 million birth”
**Reply:** Thank you for your suggestion. We have capitalized the first word of the subtitle, after the colon. Additionally, we have revised the title slightly according to your suggestion: “Risks of specific congenital anomalies in offspring of women with diabetes: A systematic review and meta-analysis of population-based studies including over 80 million birth”.

2. Financial disclosure: Please state whether any sponsors or funders (other than the named authors) played any role in: Study design, Data collection and analysis, Decision to publish, Preparation of the manuscript. If sponsors or funders had no role in the research, please include this sentence: “The funders had no role in study design, data collection and analysis, decision to publish, or preparation of the manuscript.”
**Reply:** Thank you for your suggestion. Considering the 26th comment that the reviewer this time asked us to remove the Author Contributions, Competing Interests, Data Availability Statement, and Funding from the main text, we have chosen the sentence in Financial Disclosure section in submission system: “The funders had no role in study design, data collection and analysis, decision to publish, or preparation of the manuscript.”

3. Data availability statement: Please revise the data availability statement entered into the manuscript submission system, as this is the statement that will be used in the event of publication. This currently reads “All relevant data will be made available upon request to the corresponding author via e-mail.” Please change this to:“The metadata underlying the reported analyses have been deposited in Zenodo (DOI: 10.5281/zenodo.5595068).” or similar, and please check that the DOI is correct.
**Reply:** Thank you for your suggestion. We have revised the data availability statement entered into the manuscript submission system as follows: “The metadata underlying the reported analyses have been deposited in Zenodo (DOI: 10.5281/zenodo.5783967).” Considering we uploaded the file “readme”, thus the DOI number has been changed.

4. Metadata: In the online repository of excel files, it would be helpful to include a “readme” type of file in the repository, explaining the meaning of the column headers in each spreadsheet.
**Reply:** Thank you for your suggestion. We have uploaded “readme” type of file in the repository. Please see the file at: <https://zenodo.org/record/5783967#>

5. Abstract: Lines 53-57: “Of the 23 type-specific CAs of other systems in offspring, maternal PGDM was associated with a significantly increased risk of CAs in 21 categories; the corresponding RRs ranged from 1.57 (for hypospadias, 95% CI: 1.22 to 2.02; P < 0.001) to 18.18 (for holoprosencephaly, 95% CI: 4.03 to 82.06; P = 0.085).” It should be noted that it does not seem to be the case that the RR for holoprosencephaly reached statistical significance, though the sentence is worded as if it does.
The same wording inconsistency is present at Lines 57-60. “Maternal GDM was associated with a small but significant increase in the risk of CAs in nine categories; the corresponding RRs ranged from 1.14 (for limb reduction, 95% CI: 1.06 to 1.23; P = 0.866) to 5.70 (for heterotaxia, 95% CI: 1.09 to 29.92; P = 0.008).” We suggest revising to make it clear that you are reporting 1) the number of significant associations between either PGDM or GDM and type specific CAs out of the total type-specific CAs, and then 2) reporting the highest and lowest corresponding RRs out of the total of 23 type-specific CAs.

**Reply:** Thank you for your suggestion. Actually, the P values here represented P values for heterogeneity within each subgroup, rather than the P values for the significance of relative risks. In order to eliminate the misunderstanding in our manuscript, we have deleted the P values and made the expression format in the Abstract section uniformly. Please see the revised Abstract.

6. Abstract: Lines 53-57: Please revise the description of numbers of type specific CAs and CHD examined, as the Author Summary and Discussion mention 42 categories assessed.
**Reply:** Thank you for your suggestion. We have checked throughout the manuscript and found that the number “45” was correct. We have revised the whole paper and made them consistently.

7. Abstract: Line 67; We suggest removing “with no overlap in the 95% CIs”
**Reply:** Thank you for your suggestion. We have removed “with no overlap in the 95% CIs”. Please see the revised Abstract.

8. Abstract: Line 67-69: Your findings do not directly inform on the causality of the relationship. We suggest revising to temper this conclusion. It may be helpful to focus the sentence more on how screening may inform clinical care and identifying risk.
**Reply:** Thank you for your suggestion. We have carefully revised the sentence in the Abstract section. Please see Page 4 Line 65-66.

9. Author summary: Please avoid directly copying text from the abstract for the author summary (for example, at Lines 72-75). Please revise to make this section distinct. Please see our author guidelines for more information: https://journals.plos.org/plosmedicine/s/revising-your-manuscript#loc-author-summary
**Reply:** Thank you for your suggestion. We have carefully revised the sentence in Author summary section. Please see Page 5 Line 69-70.

10. Author summary: Lines 83-85: The text here implies there are 42 categories of CAs, while the abstract describes 23 type-specific CAs. Please revise to resolve the discrepancy.
**Reply:** Thank you for your suggestion. We have checked throughout the manuscript and found that the number “45” was correct. We have revised the whole paper and made them consistently.

11. Author summary: Lines 84-85: Please revise to: “while maternal gestational diabetes is associated with a small but significant increase in the risk of CAs…”
**Reply:** Thank you for your suggestion. We have revised the sentence. Please see Page 5 Line 79-80.

12. Author summary: Lines 97-98: We suggest removing this point, as the study does not examine insulin treatment for blood glucose control during pregnancy.
**Reply:** Thank you for your suggestion. We have removed this point.

13. Introduction: Line 116: As mentioned by a reviewer, please clarify if the meaning is “third to eighth week of gestation” in this sentence.
**Reply:** Thank you for your suggestion. It means “third to eighth week of gestation”. We have revised the sentence. Please see Page 7 Line 109-110.

14. Methods: Line 142: Please include a copy of the registered protocol as supporting information, and please refer to it here (e.g. S1_Protocol).
**Reply:** Thank you for your suggestion. We have uploaded a copy of the registered protocol as supporting information (S1 Protocol).

15. Methods: Line 164: Please reference the PRISMA flowchart (e.g. Fig 1, PRISMA flowchart).
**Reply:** Thank you for your suggestion. We have referenced the PRISMA flowchart (Fig 1, PRISMA flowchart). Please see Page 10 Line 159-160.

16. Methods: Line 175: Please revise to clarify what specifically is meant by “overall CAs and CHD” and type-specific CAs for primary and secondary outcomes (e.g. rates).
**Reply:** Thank you for your suggestion. We have revised the definition of primary and secondary outcomes in our updated manuscript. Primary outcomes were the rates of overall CAs and type-specific CHD (e.g., heterotaxia; conotruncal defects; atrioventricular septal defect; anomalous pulmonary venous return; left ventricular outflow tract; right ventricular outflow tract; septal defects; and single ventricle). Secondary outcomes were the rates of other type-specific CAs (e.g., nervous system; eye, ear, face, and neck; orofacial clefts; digestive system; abdominal wall defects; genitourinary system; and musculoskeletal system). Please see Page 10 Line 171-176.

17. Results: Line 274: Please clarify the p values reported for results, for example, whether or not the risk of overall CAs in offspring of women with type 2 diabetes was statistically significant or not. Please check if the reference to Fig J1 here should be Fig L1.
**Reply:** Thank you for your suggestion. Actually, the P values here represented P values for heterogeneity within each subgroup, rather than the P values for the significance of relative risks. Therefore, in some cases there seems to be discrepancy between the 95% CIs and P values given. We have instead Fig L1 of Fig J1.

18. Results: Please clarify the p values reported for tests of significance of relative risks throughout this section, as in some cases there seems to be discrepancy between the 95% CIs and p values given.
**Reply:** Thank you for your comment. Actually, the P values here represented P values for heterogeneity within each subgroup, rather than the P values for the significance of relative risks. Therefore, in some cases there seems to be discrepancy between the 95% CIs and P values given.

19. Results: Please check all references to the figures and tables located in S1 Fig, to ensure accuracy.
**Reply:** Thank you for your suggestion. We have checked all references to the figures and tables located in S1 Fig.

20. Discussion: Line 354: Please revise to “...while maternal GDM was associated with a small but significant increase in risk of…”.
**Reply:** Thank you for your suggestion. We have revised the sentence to “...while maternal GDM was associated with a small but significant increase in risk of…”. Please see Page 28 Line 358.

21. Discussion: Line 395-397: As mentioned by a reviewer, please use consistent language when referring to stages of pregnancy. In this paragraph, it is not clear if “22 days” is in reference to fertilization or LMP, for example. At line 397, please clarify if this should be “4-7 weeks gestation” or similar.
**Reply:** Thank you for your suggestion. We have carefully revised the paragraph use consistent language based on your suggestion. Please see Page 30 Line 401-402.

22. Discussion: Line 417: Please fully define “CAKUT” in this sentence.
**Reply:** Thank you for your suggestion. We have added the full definition of CAKUT. Please see Page 31 Line 422-423.

23. Discussion: Line 432: Please check here and throughout that the number “45” is consistently the number of type-specific CAs examined (elsewhere, the number 42 is mentioned).
**Reply:** Thank you for your suggestion. We have checked throughout the manuscript and found that the number “45” was correct. We have revised the whole paper and made them consistently.

24. Discussion: Line 473: We suggest tempering this statement slightly, such as: “screening for diabetes in pregnant women may enable better glycemic control, and may enable identification of women and offspring at risk for CAs.” or similar.
**Reply:** Thank you for your suggestion. We have revised this statement slightly to “screening for diabetes in pregnant women may enable better glycemic control, and may enable identification of women and offspring at risk for CAs.” Please see Page 33 Line 480-482.

25. Table 1: Please indicate in the legend that “No” refers to “Number” within the table.
**Reply:** Thank you for your suggestion. We have indicated in the footnote that “No” refers to “Number” within the table.

26. Line 542: Please remove the Author Contributions, Competing Interests, Data Availability Statement, and Funding from the main text and please ensure all information is completely and accurately entered in the relevant fields of the manuscript submission system.
**Reply:** Thank you for your suggestion. We have removed the Author Contributions, Competing Interests, Data Availability Statement, and Funding from the main text and please ensured all information was completely and accurately entered in the relevant fields of the manuscript submission system.

27. Figure 6: Please clarify “All *P* meta-regression ˂ 0.001 for pre-gestational diabetes versus gestational diabetes.” to indicate that this is true for both the comparison within congenital anomalies and congenital heart defects between gestational diabetes and pre-gestational diabetes. Please indicate in the legend if there are any other comparisons to report.
**Reply:** Thank you for your suggestion. We have indicated in the legend “*P* meta-regression were ˂ 0.001 for both the comparison within congenital anomalies and congenital heart defects between gestational diabetes and pre-gestational diabetes.”

28. Table E and F within S1 Table file: Please move these Tables to the main text of the manuscript.
**Reply:** Thank you for your suggestion. We have moved Table E and F to the main text of the manuscript as Table 4 and Table 5.

29. Supporting Information files: The supporting information name and number are required in a caption, and we highly recommend including a one-line title as well. You may also include a legend in your caption, but it is not required. Format your supporting information captions as follows: S1 Text. Title is strongly recommended. Legend is optional.
**Reply:** Thank you for your suggestion. We have re-named and re-numbered supporting information in a one-line title as your recommendation.

30. S1 Text: We suggest the search strategy and each checklist be provided as its own supporting information file.
**Reply:** Thank you for your suggestion. We have provided search strategy in S1 Text, MOOSE checklist in S2 Text, and PRISMA 2020 checklist in S3 Text.

31. PRISMA Checklist: Please revise items 1 and 2 of the PRISMA checklist, referring to Title and Abstract rather than page numbers. We note that there is no assessment of certainty of evidence. We suggest reporting on certainty of each outcome described in the results, in light of the bias assessment reported at line 255.

**Reply:** Thank you for your suggestion. We have revised the PRISMA Checklist according to your comment. Please see the updated PRISMA Checklist in S3 Text.

**Comments from Reviewers:**

Reviewer #2: Many thanks authors for their great effort to improve the manuscript. I am satisfied with the response and revision. No further issues needing attention.
**Reply:** We would like to thank you for your helpful comments and suggestions.

Reviewer #3: The authors have addressed all my earlier comments
**Reply:** We would like to thank you for your helpful comments and suggestions.
