# Supplementary material for: Risks of specific congenital anomalies in offspring of women with diabetes: A systematic review and meta-analysis of population-based studies including over 80 million births
Source: PLoS Med. 2022 Feb 1;19(2):e1003900. doi: 10.1371/journal.pmed.1003900 (PMC8806075; doi:10.1371/journal.pmed.1003900)
Supplement: Attachment — Submitted filename: Response Letter 2021_12_22.docx [file pmed.1003900.s012.docx]

***Department of Clinical Epidemiology***

***Clinical Research Center***

***Shengjing Hospital of China Medical University***

***No. 36, San Hao Street, Shenyang***

***Liaoning 110004***

***P. R. China***

12/22/21

**Re:** **Risks of specific congenital anomalies in offspring of women with diabetes: A systematic review and meta-analysis of population-based studies including over 80 million births (PMEDICINE-D-21-01554R3)**

Dear editor,

Thank you for decision letter, informing us that our manuscript is potentially acceptable for publication in *PLoS Medicine* after a revision. We would like to thank the editor for their helpful comments and suggestions. We have carefully addressed their concerns as outlined in the attached file. Point-by-point responses are followed by this cover letter. We hope that we have adequately addressed all editor’s concerns and have made this revised manuscript acceptable for publication in *PLoS Medicine*.

Sincerely,

Shan-yan Gao, M.D., Ph.D.

Department of Clinical Epidemiology

Clinical Research Center

Shengjing Hospital of China Medical University

**Authors’ Responses to Editors’ Comments**

**Requests from the editors:**
1. Data Availability Statement: Please include the complete link to the study data in the Data Availability Statement: https://doi.org/10.5281/zenodo.5783967
**Reply:** Thank you for your suggestion. We have included the complete link to the study data in the Data Availability Statement: https://doi.org/10.5281/zenodo.5783967

2. Title: Please change the title to: “Risks of specific congenital anomalies in offspring of women with diabetes: A systematic review and meta-analysis of population-based studies including over 80 million births”
**Reply:** Thank you for your suggestion. We have changed the title to: “Risks of specific congenital anomalies in offspring of women with diabetes: A systematic review and meta-analysis of population-based studies including over 80 million births”

3. Abstract and Results: Thank you for clarifying the reporting of the p values from the tests for heterogeneity. Please report the relative risks together with both 95% CIs and p values for results described in the text. Reporting of the results for the tests of heterogeneity in the text provides additional useful information.
**Reply:** Thank you for your comment. We use STATA software to calculate pooled relative risk (RR) and 95% CI for each analysis, and there is no P value for RR and 95% CI (all figures we displayed in our manuscript are from STATA software and there is only P value for heterogeneity), which means if 95% CI for each pooled RR does NOT contain the number 1, this result has statistical significance. So according to the results from STATA software, we only reported the P values for heterogeneity.

4. Throughout: CA risk categories. In the abstract at line 52, the total number of type specific CA categories is indicated as 23. However, in the author summary and the discussion, 45 categories are mentioned. Please clarify in the Abstract that the 23 includes the CA categories that exclude the CHD-related categories.
**Reply:** Thank you for your suggestion. We have clarified the “23” CA categories throughout the revised manuscript. Please see Page 4, Line 52-54 and Page 31, Line 426.

5. Abstract: Lines 47-50: Please include the p values associated with the increase in risk for overall CA/CHD in women with PGDM or GDM.
**Reply:** Thank you for your comment. We have added P values for heterogeneity in the abstract.

6. Results: Lines 265-274: Please include the p values for RR for CAs associations with PGDM, type 1 diabetes, type 2 diabetes (p value associated with heterogeneity test may be reported separately).
**Reply:** Thank you for your comment. Similar with Comment No. 3, because of using STATA software to calculate pooled relative risk (RR) and 95% CI for each analysis, there is no P value for RR and 95% CI (all figures we displayed in our manuscript are from STATA software and there is only P value for heterogeneity), which means if 95% CI for each pooled RR does NOT contain the number 1, this result has statistical significance. So according to the results from STATA software, we only reported the P values for heterogeneity.

7. Results: Lines 278-288: Please include the p values for RR for CHD associations with PGDM, type 1 and type 2 diabetes (p value associated with heterogeneity test may be reported separately).
**Reply:** Thank you for your comment. Similar with Comment No. 3, because of using STATA software to calculate pooled relative risk (RR) and 95% CI for each analysis, there is no P value for RR and 95% CI (all figures we displayed in our manuscript are from STATA software and there is only P value for heterogeneity), which means if 95% CI for each pooled RR does NOT contain the number 1, this result has statistical significance. So according to the results from STATA software, we only reported the P values for heterogeneity.

8. Results: Lines 290-300: Please include p values for tests for RR for GDM associations with CHD (p value associated with heterogeneity test may be reported separately).
**Reply:** Thank you for your comment. Similar with Comment No. 3, because of using STATA software to calculate pooled relative risk (RR) and 95% CI for each analysis, there is no P value for RR and 95% CI (all figures we displayed in our manuscript are from STATA software and there is only P value for heterogeneity), which means if 95% CI for each pooled RR does NOT contain the number 1, this result has statistical significance. So according to the results from STATA software, we only reported the P values for heterogeneity.

9. Results: Lines 303-314: Please include the p values for the RR reported for GDM and PGDM associated with type-specific CAs (p value associated with heterogeneity test may be reported separately).
**Reply:** Thank you for your comment. Similar with Comment No. 3, because of using STATA software to calculate pooled relative risk (RR) and 95% CI for each analysis, there is no P value for RR and 95% CI (all figures we displayed in our manuscript are from STATA software and there is only P value for heterogeneity), which means if 95% CI for each pooled RR does NOT contain the number 1, this result has statistical significance. So according to the results from STATA software, we only reported the P values for heterogeneity.

10. Lines 553-555: Please remove the Financial Disclosure section from the main text, and please make sure all information is accurately entered in the Funding section of the manuscript submission system. You note here that the funders had no role in the study. However, the Funding section of the manuscript notes that “The authors received no specific funding for this work.” Please clarify.
**Reply:** Thank you for your suggestion. We have removed the Financial Disclosure section from the main text and made sure all information is accurately entered in the Funding section of the manuscript submission system.

11. Figures 2, 3, 4, and 5: Please report the overall RR with 95% CIs and p values. Please define “DL” in the legends.
**Reply:** Thank you for your comment. We have reported the overall RR with 95% CIs, and defined “DL” in the legends in Figures 2, 3, 4, and 5.

12. Figure 6: Please report the p value in addition to the RR and 95% CIs.
**Reply:** Thank you for your comment. Similar with Comment No. 3, because of using STATA software to calculate pooled relative risk (RR) and 95% CI for each analysis, there is no P value for RR and 95% CI (all figures we displayed in our manuscript are from STATA software and there is only P value for heterogeneity), which means if 95% CI for each pooled RR does NOT contain the number 1, this result has statistical significance. So according to the results from STATA software, we only reported the P values for heterogeneity.

13. Tables 2, 3, 4, and 5: Please report the p values in addition to the 95% CIs for the pooled RRs.
**Reply:** Thank you for your comment. Similar with Comment No. 3, because of using STATA software to calculate pooled relative risk (RR) and 95% CI for each analysis, there is no P value for RR and 95% CI (all figures we displayed in our manuscript are from STATA software and there is only P value for heterogeneity), which means if 95% CI for each pooled RR does NOT contain the number 1, this result has statistical significance. So according to the results from STATA software, we only reported the P values for heterogeneity.

14. Figures G - L in S1 Fig: Please note the overall RR with 95% CIs and p values. Please define the abbreviation “DL” in the legends.
**Reply:** Thank you for your suggestion. We have noted the overall RR with 95% CIs, and defined the abbreviation “DL” in the legends in Figures G - L. Please see the update S1 Fig.

15. Line 58-60, Line 330-332, Line 435-437 and Line 442-444: There seems to be some overlap in text with https://doi.org/10.1136/bmj.m3222. Please revise to avoid this.
**Reply:** Thank you for your suggestion. We have revised the sentences according to your suggestion. Please see Page 4, Line 58-59, Page 22, Line 332-334, Page 31, Line 439-440.
